# Effect of ultra-violet light radiation on *Scenedesmus vacuolatus* growth kinetics, metabolic performance, and preliminary biodegradation study

**DOI:** 10.1007/s10532-023-10029-2

**Published:** 2023-04-13

**Authors:** Stella B. Eregie, Isaac A. Sanusi, Gueguim E. B. Kana, Ademola O. Olaniran

**Affiliations:** 1 School of Life Sciences, University of KwaZulu-Natal, Private Bag, X01, Scottsville 3209, Pietermaritzburg, South Africa; 2 Fort Hare Institute of Technology, University of Fort Hare, Private Bag X1314, Alice, 5700 South Africa; 3 School of Life Sciences, University of KwaZulu-Natal, Westville Campus, Private Bag X54001, Pietermaritzburg, South Africa

**Keywords:** UV radiation, *Scenedesmus vacuolatus*, Growth kinetics, Substrate affinity, Biodegradation

## Abstract

This study presents the effect of ultra-violet (UV) light radiation on the process kinetics, metabolic performance, and biodegradation capability of *Scenedesmus vacuolatus*. The impact of the UV radiation on *S. vacuolatus* morphology, chlorophyll, carotenoid, carbohydrates, proteins, lipid accumulation, growth rate, substrate affinity and substrate versatility were evaluated. Thereafter, a preliminary biodegradative potential of UV-exposed *S. vacuolatus* on spent coolant waste (SCW) was carried out based on dehydrogenase activity (DHA) and total petroleum hydrocarbon degradation (TPH). Pronounced structural changes were observed in *S. vacuolatus* exposed to UV radiation for 24 h compared to the 2, 4, 6, 12 and 48 h UV exposure. Exposure of *S. vacuolatus* to UV radiation improved cellular chlorophyll (chla = 1.89-fold, chlb = 2.02-fold), carotenoid (1.24-fold), carbohydrates (4.62-fold), proteins (1.44-fold) and lipid accumulations (1.40-fold). In addition, the 24 h UV exposed *S. vacuolatus* showed a significant increase in substrate affinity (1/Ks) (0.959), specific growth rate (µ) (0.024 h^−1^) and biomass accumulation (0.513 g/L) by 1.50, 2 and 1.9-fold respectively. Moreover, enhanced DHA (55%) and TPH (100%) degradation efficiency were observed in UV-exposed *S. vacuolatus*. These findings provided major insights into the use of UV radiation to enhance *S. vacuolatus* biodegradative performance towards sustainable green environment negating the use of expensive chemicals and other unfriendly environmental practices.

**Graphical abstract:**

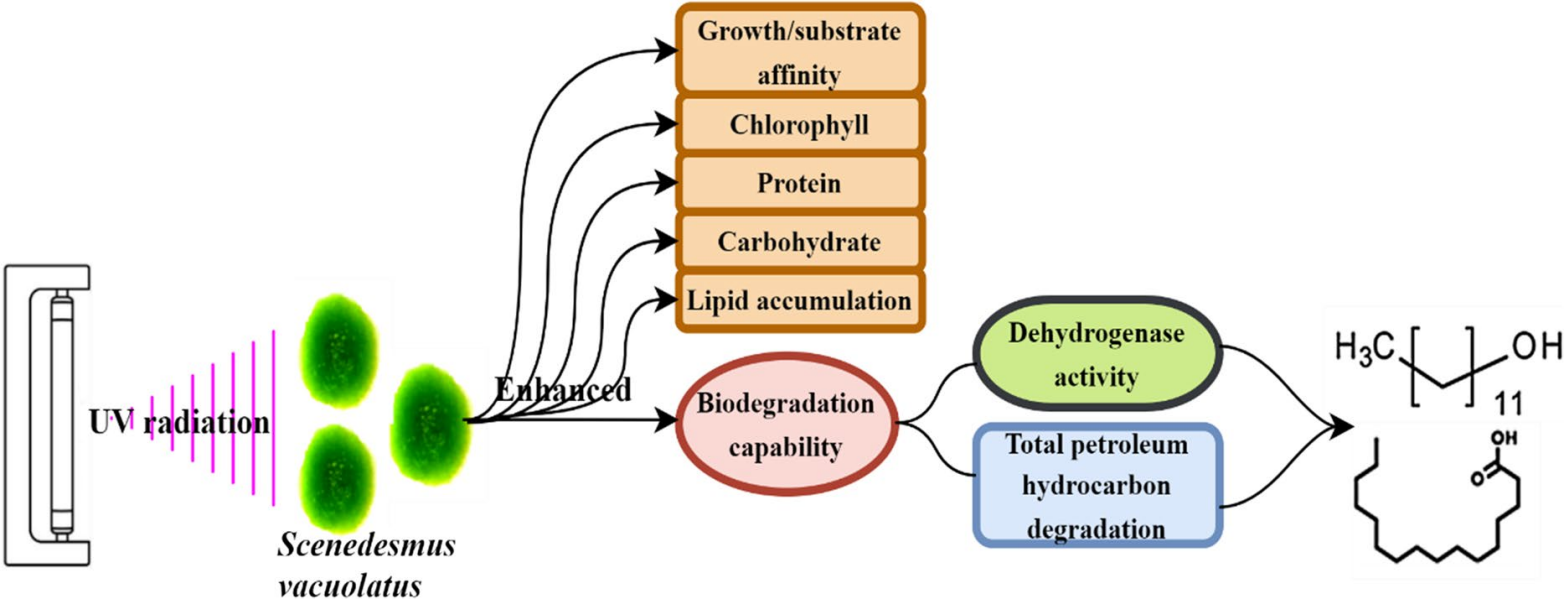

**Supplementary Information:**

The online version contains supplementary material available at 10.1007/s10532-023-10029-2.

## Introduction

The illegal disposal of spent oil waste into the environment has sparked a lot of concern worldwide due to its impact on human health, plants, animals, and microorganisms (Rathi and Yadav 2019; Saha et al. 2019). The urgent need and search for a cost-effective and environmentally friendly clean-up approach that can mitigate the harmful effect of these oil waste pollutants is of great importance to the future sustainability of the environment. Various strategies such as landfilling, high-temperature incineration, chemical decomposition and recycling have been used to treat spent oil waste (Kajdas 2014). These methods are not only too expensive to provide a satisfactory solution but create another environmental concern, such as generating more greenhouse gases (dioxins, carbon monoxide, nitrogen dioxide, sulphur dioxide). These gases contaminate the atmosphere and destroy the ozone layer resulting in severe damage to the environment (Asgari et al. 2017; Sihag and Pathak 2016). The use of microbes such as microalgae to degrade lubricant oil waste has gained global attention due to its sustainability and environmental benefits. The use of microalgae to degrade lubricant oil waste has numerous advantages over existing traditional and chemical treatment methods. These include the ability to thrive in a variety of polluted environments with extreme conditions, production of enzymes for quick metabolism of pollutants, conversion of organic pollutants into useful environmentally friendly compounds, cost-effectiveness, relatively safe and ecologically sustainable. Microalgae treatment's investment and economic cost are 5–20 times less than traditional and chemical treatment (Touliabah et al. 2022). Studies using different green microalgae such as the species (sp) of *Chlamydomonas*, *Selenastrum, Scenedesmus Cyanobacteria* and *Chlorella* to treat lubricant oil waste have been reported (Dell' Anno et al. 2021; Radziff et al. 2021; Touliabah et al. 2022). *Scenedesmus* species (sp) has been recognized as one of the most effective microalgae in oil waste biodegradation due to their strong photosynthetic activity and cell surface area (Gonçalves 2021; Ismail et al. 2020).

*Scenedesmus* belong to the class of *Chlorophyceae* (Akgül et al. 2017). They are among the few photosynthetic microalgae that have been mass-produced for food, feed and high-value natural products globally (Sarwa and Verma 2017). It is a microalgae with unique characteristics: (a) rapid growth rate, (b) high accumulation of biomass and easy to harvest, (c) high source of natural products such as oils, pigments, protein, fatty acids and lipids, (d) it can be cultivated in low-cost medium and the presence of recalcitrant organic pollutants (e) high carbon fixation ability, (f) its ability to grow at extreme conditions such as high temperature and pH (Baghour 2019; El-Sheekh et al. 2013). Even though microorganisms can resist a wide range of recalcitrant organic pollutants under environmental pollution stress, this approach becomes inefficient in the presence of some recalcitrant organic pollutants such as spent lubricant oil waste. With the recent advancement in molecular biology and genetic engineering, novel approaches to solving the degradation of xenobiotic pollutants have been made possible (Fajardo et al. 2020; Kumar et al. 2020). Despite the depth of research on *Scenedesmus* for environmental pollutant degradation, there is a dearth of knowledge on improving their biodegradative potential by improving cellular metabolic performance. A study on strain improvement of *Scenedesmus vacuolatus* for improving spent lubricant oil waste degradation is desirable, cheaper and sustainable. Strain improvement through mutagenesis has been reported to be an effective method to enhance microalgae metabolic performance and degradative capabilities (Arora et al. 2020). Mutagenesis in microalgae is commonly carried out by physical or chemical mutagens. Physical mutagens involve the exposure of microalgae to radiation such as ultraviolet (UV) radiation, gamma-ray, and X-rays, whereas the chemical mutagens involve the use of chemicals including ethyl methanesulfonate, hydroxylamine, nitrous acid and methylnitronitrosoguanidine (Hlavova et al. 2015; Thurakit et al. 2018). In this study, mutagenesis by UV light radiation is of significant interest because it is considered efficient, simple, rapid, relatively safe and does not involve any specialized training for its implementation (Lim and Schenk 2017). Furthermore, microalgae strains obtained by mutagenesis are not subject to the same stringent regulatory constraints as genetically modified microorganisms, necessitating no legal or regulatory approval before commercial use. The UV radiation induces random mutation in the microorganism deoxyribonucleic acid via base pair deletion, addition and substitution resulting in new sets of pyrimidines dimer and thymine dimers (Choi et al. 2017; Fu et al. 2016; Moha-León et al. 2019; Sydney et al. 2018). Owing to this UV mutagenesis method's simplicity, it has been employed to obtain microalgae strains with improved metabolic and physiological processes (Arora et al. 2020). For instance, an increase in growth, biomass concentration, carbohydrates, protein, lipids and chlorophyll contents were observed with *C. vulgaris* after UV radiation exposure (Sarayloo et al. 2018). Likewise, the exposure of *Nannochloropsis oculata* to UV radiation resulted in a higher growth rate and production of several lipids and fatty acids (Moha-León et al. 2019). The authors also reported on the release of defense biomolecules by *Nannochloropsis oculate* to protect itself from the effects of the UV radiation. Many microalgae species have been reported to have a variety of protective mechanisms against UV radiation, including DNA repair, production of antioxidants and UV-absorbing compounds such as mycosporine-like amino acids (MAAs) and scytonemin (Pessoa 2012; Rastogi et al. 2020). However, with the abundance of literature on the effect of UV radiation on microalgae, no research on the impact of UV radiation on *Scenedesmus vacuolatus* has been reported. A study on UV mutagenesis of *S. vacuolatus* for improved spent oil degradation could provide insight into *S. vacuolatus* with improved metabolic and degradative performance. Therefore, this study investigates the effect of the physical mutagen UV radiation on *S. vacuolatus* cellular structure, metabolic performances and growth for improved spent oil degradation. Thereafter, the UV exposed *S. vacuolatus* was assessed for biodegradation of waste lubricant oil.

## Materials and methods

### Culture medium composition

Blue-green 11(BG11) medium containing three different solutions (solution A, solution B and solution C) was used in this study. Solution (A) contains the following in (g/L) 2.86 g H_3_BO_3_, 1.81 g MnCl_2_·4H_2_O, 0.22 g ZnSO_4_·7H_2_O, 0.39 g Na_2_MoO_4_·2H_2_O, 0.08 g CuSO_4_·5H_2_O and 0.05 g CO(NO_3_)_2_·6H_2_O. Solution (B) in (500 mL) contains 2.0 g K_2_HPO_4_, 3.75 g MgSO_4_·7H_2_O, 1.80 g CaCl_2_.2H_2_O, 0.30 g Citric acid, 0.30 g Ammonium Ferric Citrate green, 0.05 g EDTANa_2_, and 1.0 g NaCO_2_. Solution (C) in (g/L) contains 15.0 g NaNO_3_. To make 1L BG11 broth medium, 1 mL of solution (A), 10 mL of solution (B) and 100 mL of solution (C) were taken from each sock solution. For the solid BG11 agar medium, 15.0 g of bacteriological agar was added to the 1L BG11 medium. The pH was adjusted to 7.0 using 1 M NaOH or HCl prior to sterilization by autoclave at 121 °C for 15 min.

### *S. vacuolatus* UV exposure

*Scenedesmus vacuolatus* isolated from spent lubricant oil waste (Eregie and Jamal 2019) was used for the UV mutagenesis in this study. Kultschar et al. (2019) method was modified for the UV mutagenesis experiment. Colonies of *S. vacuolatus* were inoculated on BG11 agar plate and allowed to grow for 14 days under standard conditions. The obtained *Scenedesmus vacuolatus* colonies were exposed to UV light (UV-C) radiation at a distance of 15 cm and at a wavelength of 254 nm with an intensity of 1.4 mW/cm^2^ for different time duration of 2, 4, 6, 12, 24 and 48 h. After the UV radiation exposure, colonies of the exposed cells were re-incubated under a 24 h darkness at 25 °C. The re-incubated cells were further subcultured onto a fresh BG11 agar plate under a 12 h daylight/darkness cycle at 25 °C for 2 weeks. The UV-exposed colonies at different time intervals were randomly selected based on the colony's visibility for characterization and preliminary biodegradation study.

### Microscopy morphological characterization

Morphological characterization of both wild-type and UV-exposed microalgae was carried out using stereo microscopy (SM) (LEICA MZ16, with camera LEICA DFC450C), bright field microscopy (BFM) (OLYMPUS A × 70) and scanning electron microscopy (SEM) (ZEISS EVO L515ss). The SM at different magnification was used to view directly the microalgae colonies grown on BG11 agar to capture the morphological features. While for BFM, a drop of microalgae aliquot grown in BG11 medium was pipetted on a clean glass slide, then one drop of formaldehyde to fix the microalgal cells for five minutes before the BFM analysis. The SEM was used for further morphological characterization of the microalgal cells. Samples were prepared by pipetting a drop of the microalgal sample securely onto an aluminium SEM stub with double-sided carbon tape. This was allowed to dry completely. The samples were sputter-coated with a gold coat using Quorum Q150RES before the SEM viewing.

### Growth condition

The wild-type and UV-exposed *S. vacuolatus* (10% v/v) separately were inoculated into 100 mL BG11 medium in 250 mL Erlenmeyer flasks. The detailed BG11 growth medium constituent is described in section "Culture medium composition". The culture flasks were incubated at 25 °C for 21 days and sampling were carried out at regular interval (Eregie and Jamal-Ally 2019, 2021). Samples were thereafter analyzed for biomass concentration, cellular biomolecules (chlorophyll, carotenoid, protein, carbohydrate and lipid contents), growth rate, substrate versatility and substrate affinity.

### Substrate versatility study

Substrate versatility of the UV-exposed *S. vacuolatus* using different substrate types was determined by replacing the BG11 medium as the only carbon source with sodium carbonate Na_2_CO_3_, glucose and glycerol differently. Growth was monitored at 25 °C for 21 days and sample for analysis was taken every 3 days.

### Preliminary biodegradation of spent coolant waste setup

To evaluate the degradation efficiency of UV exposed *S. vacuolatus* using spent coolant waste (SCW) as substrate, biodegradation analysis was carried out. The experimental setup was carried out in BG 11 medium with a working volume of 100 mL containing 10 mL of the wild or UV exposed *S. vacuolatus* separately and 10% (v/v) of SCW as the only carbon source. The flasks were agitated on a shaker incubator at 25 °C for five weeks under white fluorescent light (12 h daylight followed by 12 h of darkness cycle). The control experiment was done with BG11 medium having SCW without inoculum. Samples were taken each week from different flasks and analyzed for dehydrogenase activity and total petroleum hydrocarbon. Total petroleum hydrocarbon and dehydrogenase activity were determined using previously established protocols by Irawan et al. (2018) and Ding et al. (2013), respectively.

### Analytical methods

Microalgal biomass concentration (cell dry weight) was obtained by measuring the optical density at 680 nm using SpectroVis plus Spectrophotometer (USA). The cell dry weight was then determined using a standard calibration curve, a correlation dependence of biomass dry weight as a function of optical density (Savvidou et al. 2021).

The chlorophyll composition was estimated using the method described by Tomar and Jajoo (2021). 5 mL of microalgae culture was taken and centrifuged at 5000 rpm for 5 min. The supernatant was discarded, and 5 mL of 99.9% methanol was added to the microalgae pellet, mixed properly and incubated at 90 °C for 5 min. Then the sample was centrifuged at 10,000 rpm for 5 min. The supernatant was collected, and the optical density was measured at 666, 653, and 470 nm for chlorophyll A (Chl *a*), chlorophyll B (Chl *b*) and carotenoid (Cx), respectively. The actual chlorophyll estimation was afterward calculated as follows:

1 $$\begin{gathered} {\text{Chl}}a\left( {\mu {\text{g}}/{\text{mL}}} \right) - {15}:{\text{65A}}_{{{666}}} - {7}:{34}0{\text{A}}_{{{653}}} \hfill \\ {\text{Chl}}b\left( {\mu {\text{g}}/{\text{mL}}} \right) = {27}:0{\text{5A}}_{{{653}}} - {11}:{\text{21A}}_{{{666}}} \hfill \\ {\text{Cx}} + {\text{c }}\left( {\mu {\text{g}}/{\text{mL}}} \right) = {{\left( {{1}000{\text{A}}_{{{47}0}} - {2}:{\text{86 Chl}}a - {129}:{\text{2 Chl}}b} \right)} \mathord{\left/ {\vphantom {{\left( {{1}000{\text{A}}_{{{47}0}} - {2}:{\text{86 Chl}}a - {129}:{\text{2 Chl}}b} \right)} {{245}}}} \right. \kern-0pt} {{245}}} \hfill \\ \end{gathered}$$

The total protein content was obtained using the previously described protocol by Ganapathy (2017) and Sanusi et al. (2020). The protein concentration was then extrapolated by a predetermined standard calibration curve using bovine serum albumin as the standard (Bradford method 1976).

Carbohydrate content was obtained by transferring 2 mL of microalgae samples into centrifuge tubes containing saturated mercuric chloride (0.1 mL). Mercuric chloride was used to inhibit any further substrate utilization by the microalgae cells. The mixed samples were centrifuged at 5000 rpm for 10 min. Afterward, 1 mL aliquot of the solution was extracted for the total carbohydrate determination using the Anthrone-sulphuric method. Total carbohydrate was then deduced from a standard curve using Anthrone reagent with glucose as standard at 620 nm (Sanusi et al. 2020).

The residual glucose concentration in the supernatant (sample centrifuge at 10,000 rpm for 5 min) was determined using the dinitrosalicylic acid (DNS) method (Miller 1959). Sugar utilization was then determined using Eq. (2).

2 $$Sugar\,utilization\, = \,\frac{Initial\, sugar\, concentration\, - \,final \,sugar \,concentration}{{Initial\, sugar\, concentration}} \, \times \,100$$

The microalgae lipid content was obtained by solvent extraction method (Bligh and Dyer 1959). One millilitre of aliquot was centrifuged and the pellet with 80 mL distilled water was homogenized and heated at 2450 MHz for 5 min using a 1000 W capacity Samsung microwave oven (Model: ME9114S1, Malaysia). Thereafter, 10 mL and 20 mL of chloroform and methanol were added to the disrupted cells and vortexed for 10 min. Chloroform (10 mL) was further added to the mixture with 10 min homogenization. The mixture was vortexed for an additional 10 min after adding 10 ml of distilled water. The final mixture was filtered using acrodisc syringe filters to obtain a two-layer filtrate (lipid + chloroform) and the chloroform was evaporated from the bilayer. Then the lipid content was determined gravimetrically.

### Kinetic calculations

Then the specific growth rate values (μ) and the initial substrate concentration value was used to estimate the maximum specific growth rate (μ_max_) and Monod constant (Ks).

3 $$Specific\,growth\, rate\, \left( \mu \right) = \frac{{InX_{2 } - InX_{1} }}{{\left( {t_{2 } - t_{1} } \right)}}$$

where µ is the specific microalgae growth rate, X_2_ and X_1_ were the biomass concentration at time T_2_ and T_1,_ respectively. The linear representation of this equation is expressed as follows:

4 $$\frac{1}{\mu } = \frac{1}{{\mu_{max} }} + \frac{{K_{s} }}{{\mu_{max} }} \left( \frac{1}{S} \right)$$

where S is the substrate concentration.

Moreover, the logistic model (Eq. 4) was employed to depict the correlation of *S. vacuolatus* biomass (X), at particular time (t) during the log and the stationary growth phase of *S. vacuolatus* to initial biomass concentration (X_0_), maximum biomass concentration (X_max_) and maximum specific growth rate (µ_max_).

5 $$X = \frac{{X_{0} \cdot exp\left( {\mu_{max} \cdot t} \right)}}{{1 - \left( { \frac{{X_{0} }}{{X_{max} }}} \right) \cdot \left( {1 - exp\left( {\mu_{max} \cdot t} \right)} \right)}}$$

The kinetic growth constant was estimated by fitting a linear function to exponential phase. This was calculated based on the following equation.

6 $$ln\left( \frac{X}{Xo} \right) = K_{g} t$$

where X is the growth of microalgae as function of time, X_o_ is the initial growth of microalgae, k_g_ is the kinetic growth constant.

### Statistical analysis

The data obtained from each experiment was analyzed with a one-way analysis of variance (ANOVA), using the Statistical Package for the Social Sciences (SPSS) version 28. The computed data are means of 3 replicates (n = 3) and all results are expressed as mean ± standard error for each strain. The significant difference was performed at a 5% level of significance.

## Results and discussion

### Effect of UV radiation on *S. vacuolatus* structural morphology

**Fig. 1:**
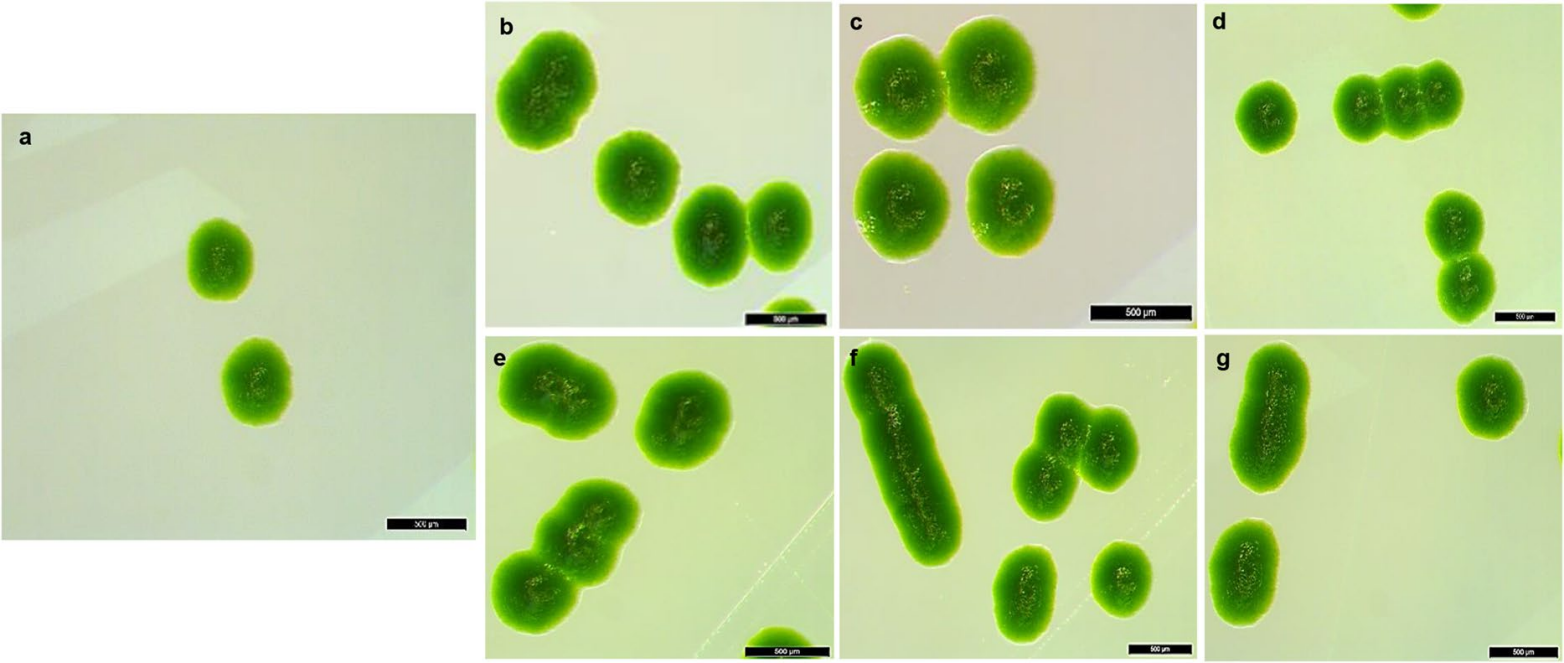
Colonies of microalgae strains as observed under stereo microscope (500 μm). **a** Wild *S. vacuolatus* microalgae, and **b**, **c**, **d**, **e**, **f**, and **g** are UV exposed *S. vacuolatus* for 2, 4, 6, 12, 24 and 48 h, respectively

**Fig. 2:**
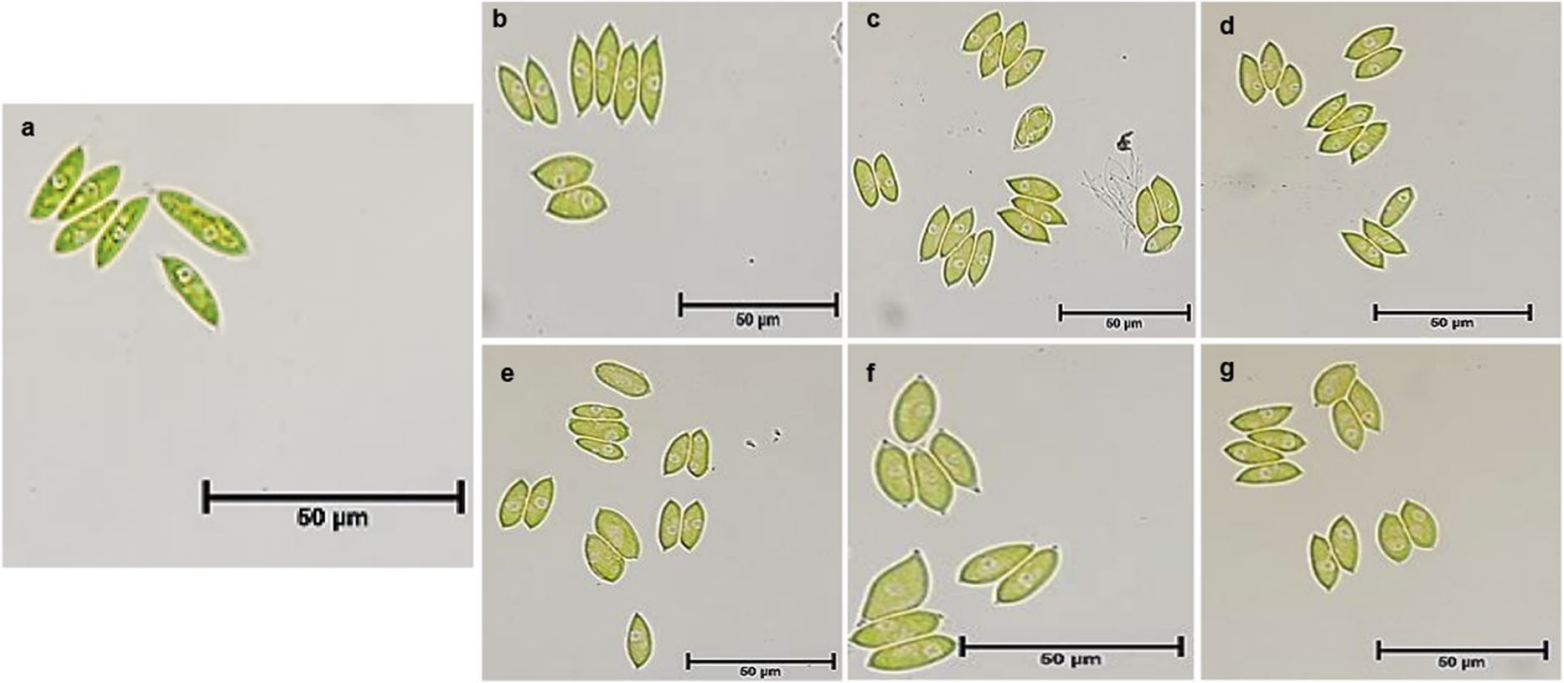
Brightfield microscopy (BFM) micrograph of microalgae cells under × 40 magnification. **a** Wild-type microalgae (unexposed); **b**, **c**, **d**, **e**, **f**, and **g** *S. vacuolatus* exposed to UV light at different time intervals (2, 4, 6, 12, 24, and 48 h) respectively

**Fig. 3:**
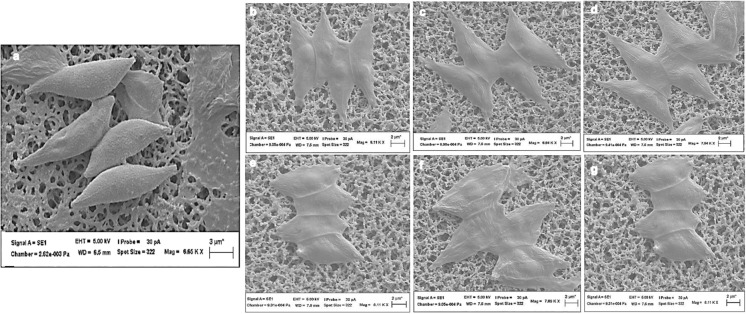
Scanning electron microscopy (SEM) micrograph of microalgae cells. **a** Wild S. vacuolatus, **b**, **c**, **d**, **e**, **f**, and **g** are UV exposed S. vacuolatus for 2, 4, 6, 12, 24 and 48 h respectively

The UV-exposed *S. vacuolatus* showed structural cellular modifications and arrangement (Figs. 1, 2, 3). Exposed cells underwent cellular fusion, elongation and cell-to-cell pairing, SM (Fig. 1b–g), BFM (Fig. 2b–g) and SEM (Fig. 3b–g) microscopy in comparison with the wild-type (Figs. 1a, 2a and 3a) respectively. Other studies have observed similar cellular morphological alterations in *Scenedesmus* sp after exposure to mutagen (Akgül et al. 2017; Beherepatil and Deore 2013; Sarwa and Verma 2017). The cell elongation, cell fusion and pairing of cells could be ascribed to the effect of the UV radiation on *S. vacuolatus* during cellular development (Choi et al. 2017; Colina et al. 2020). The elongation of the cells and cell fusion increases the cell surface area, which has the potential to improved *S. vacuolatus* photosynthetic activities and cellular metabolic performance. Microalgae with higher surface area to volume ratio and large cell size are good bioremediators (Subashchandrabose et al. 2013). Moreover, the cell's morphological modification, especially the concave shape, could also improve the photosynthetic activities through improved angulation of the light ray on the cell wall. Similarly, the non-damage to the green chlorophyll pigment is an indication of the cell integrity not been damaged, this desirable since chlorophyll is a photosynthetic apparatus (Choi et al. 2017; Sarwa and Verma 2017). The findings in this study were consistent with earlier studies on using UV light radiation for microalgae mutagenesis (Carino et al. 2022; Colina et al. 2020). The differences in cellular modifications observed in this study compared to other studies can be attributed to the differences in the choice of microalgae species, the length of UV exposure and the operational condition implemented (Carino et al. 2022; Choi et al. 2017; Colina et al. 2020).

### Effect of UV radiation exposure on *S. vacuolatus* growth

**Fig. 4:**
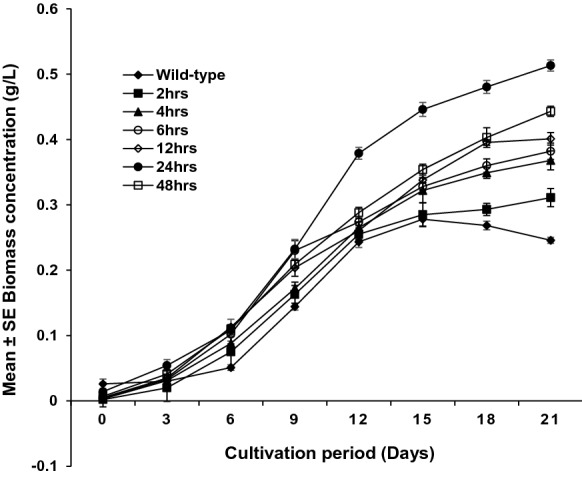
The growth profile of the wild *S. vacuolatus* (unexposed *S. vacuolatus*) and UV exposed *S. vacuolatus* at different time interval (2, 4, 6, 12, 24, 48 h). Mean ± standard error of three replicates (n = 3)

**Fig. 5:**
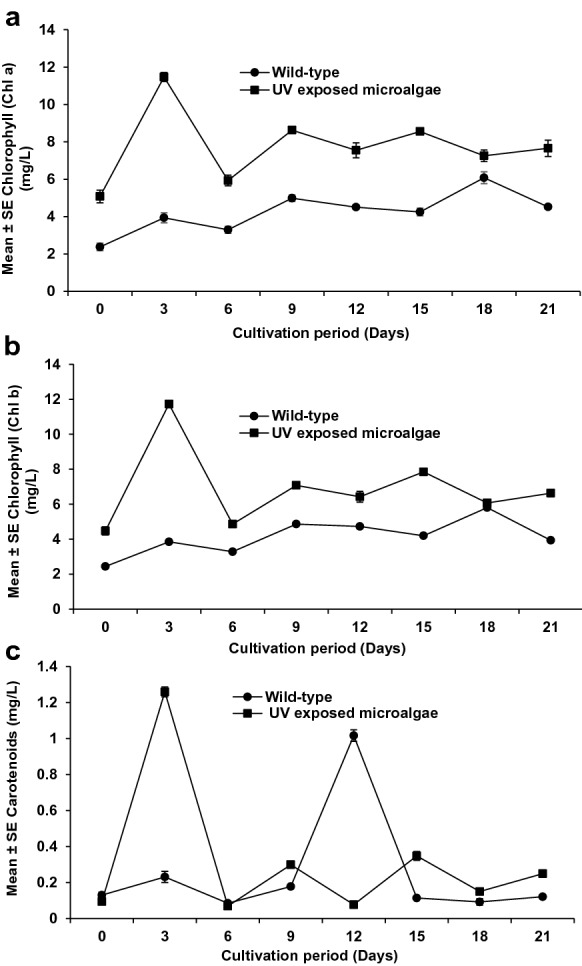
Effect of UV light radiation on the total chlorophyll content of wild *S. vacuolatus* and UV exposed *S. vacuolatus.* **a** Chlorophyll a; **b** chlorophyll b; **c** carotenoids. Mean ± standard error of three replicates (n = 3)

The growth curves of the UV-exposed *S. vacuolatus* at different exposure times are shown in Fig. 4. A shortened lag period and a sharp extended exponential phase was observed for the 24 h UV exposed *S. vacuolatus* compared with the growth patterns obtained for 2, 4, 6, 12, 48 h UV exposed *S. vacuolatus* and the wild *S. vacuolatus*. Similarly, the specific growth rate (*µ*) of the 24 h UV exposed *S. vacuolatus* (0.024 day^−1^) was higher compared to 0.015, 0.017, 0.018, 0.019, 0.020 and 0.012 day^−1^ obtained for 2, 4, 6, 12, 48 h UV exposed *S. vacuolatus* and the wild *S. vacuolatus*, respectively. Furthermore, the obtained biomass concentration (0.513 ± 0.008 g/L) of the 24 h UV exposed *S. vacuolatus* after 21 days of cultivation period showed significant increase (p < 0.001) in biomass concentration in comparison to 0.311 ± 0.009 g/L, 0.368 ± 0.015 g/L, 0.382 ± 0.013 g/L, 0.401 ± 0.009 g/L, 0.443 ± 0.008 g/L and 0.268 ± 0.005 g/L, for 2, 4, 6, 12, 48 h UV exposed *S. vacuolatus* and the wild-type respectively. The high µ and biomass concentration values obtained for the UV-exposed *S. vacuolatus* suggest the impact of UV radiation on *S. vacuolatus* growth. Also, the substantial improvement in specific growth rate observed in the study could be attributed to improved substrate affinity observed with the UV-exposed *S. vacuolatus*. Higher growth rates due to substrate availability and enhanced affinity could trigger certain cellular activities, thus increasing photosynthetic capacity and productivity. Likewise, the higher growth rates and biomass accumulation observed in the present study by the UV-exposed *S. vacuolatus* could be ascribed to the impact of UV light radiation on *S. vacuolatus* photosynthetic apparatus (chlorophyll a and b, carotenoids, large and elongated cell size) (Figs. 1 and 5), that could have resulted in enhanced photosynthetic activities (Subashchandrabose et al. 2013). The modification of the cellular structure through UV exposure has been reported to potentially improve both photosynthetic and metabolic activities of microalgae species (Liu et al. 2015). Amongst the UV-exposed *S. vacuolatus*, 24 h UV-exposed *S. vacuolatus* showed the best desirable traits such as increased chlorophyll content, enhanced substrate affinity, high sugar utilization and growth rate compared to other UV-exposed *S. vacuolatus*. Hence, the 24 h UV-exposed *S. vacuolatus* was selected for further studies.

### Chlorophyll-carotenoid content of UV exposed *S. vacuolatus*

The chlorophyll and carotenoid contents of the 24 h UV exposed *S. vacuolatus* is presented in Fig. 5a–c. Significantly (p < 0.001) higher Chl a (11.470 ± 0.003 μg/mL), Chl b (11.725 ± 0.002 μg/mL) and carotenoid (1.259 ± 0.008 μg/mL) pigment were observed for the 24 h UV exposed *S. vacuolatus* compared to the wild-type (Chl a = 6.080 ± 0.006 μg/mL, Chl b = 5.806 ± 0.003 μg/mL and carotenoid = 1.016 ± 0.009 μg/mL). This is 1.89, 2.02 and 1.24-fold higher than the wild *S. vacuolatus* pigments, respectively. High Chl a, Chl b and carotenoid pigment obtained for the UV-exposed *S. vacuolatus* indicate a positive impact of the UV radiation on the pigment production apparatus (Gonçalves 2021). Improved pigmentation could be attributed to alterations in the genetic make-up responsible for pigment production. High pigmentation may enhance the capturing of light energy and consequently, high photosynthetic activities. It has been reported that Chl a, Chl b and carotenoid are the principal photosynthetic biomolecular apparatus in microalgae that captures light energy and CO_2_. These photosynthetic apparatuses are also UV filters to protect the photosystems from the damaging effects of UV light radiation (El-Sheekh et al. 2021). A similar increase in chlorophyll contents by *Chlorella* sp and *Chlamydomonas reinhardtii* was reported by Liu et al. (2015) after their exposure to UV radiation. On the other hand, the total Chl content of 24 h UV-exposed *S. vacuolatus* in the current study differs from those of Kumar et al. (2018). They reported a decrease in the chlorophyll contents of *C. sorokiniana* (Chl a 1.51 μg/mL, Chl b 0.49 μg/mL), and carotenoid (0.81 μg/mL) after UV exposure. These variances in the Chlorophyll contents are most likely due to variations in exposure time, microalgae species and growth conditions employed.

### Protein, carbohydrate, and lipid accumulation in UV exposed *S. vacuolatus*

**Fig. 6:**
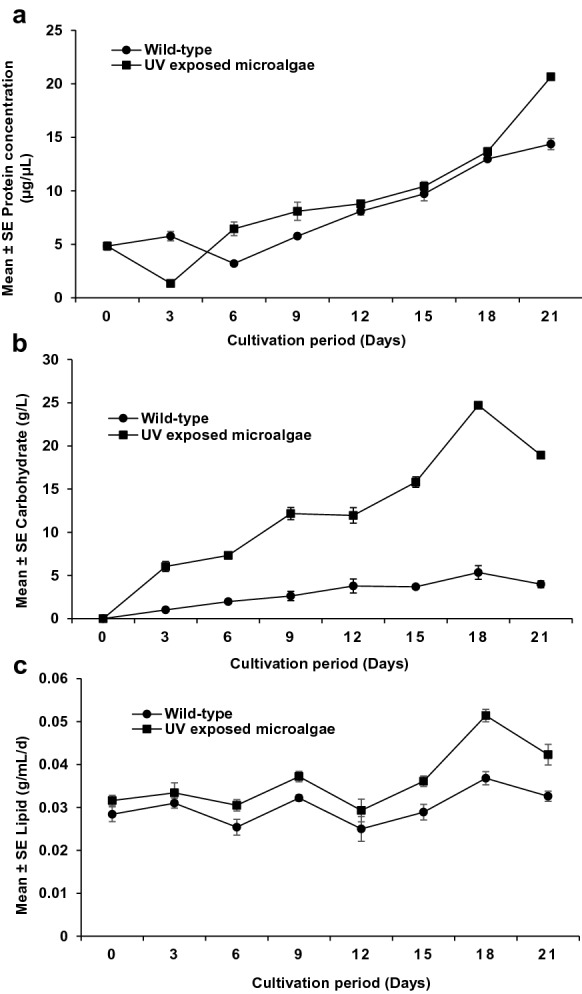
Effect of UV light radiation on the total protein, carbohydrate, and lipid content of UV exposed S. vacuolatus. **a** Protein; **b** carbohydrate; **c** lipid. Mean ± standard error of three replicates (n = 3)

The protein content in the 24 h UV-exposed *S. vacuolatus* was 20.651 ± 0.003 g/L (Fig. 6a). This was 1.44 times significantly (p < 0.001) higher than that of the wild-type (14.372 ± 0.010 g/L). This increase in protein content can be attributed to the impact of UV radiation on the cellular protein machinery. On the hand, the ratio of protein to biomass (P:B) accumulation shows the wild-type (54:1) has higher P:B compared to the UV-exposed*. vacuolatus* (1:40). UV radiation has been used to positively bioengineer microorganisms to improved cellular biomolecules such carbohydrate and lipid (Ganapathy et al. 2017; Tenorio et al. 2022; El-Sheekh et al. 2021). The UV radiation in the present study favoured protein synthesis and accumulation and subsequently enhanced the *S. vacuolatus* enzymatic and metabolic activities. Proteins are one of the most abundant components in microalgae, accounting for up to 50% of total cell weight. Thus, alteration in the protein concentration in the cell could positively affect the overall microalgal metabolic performance (Colina et al. 2020). Moreover, the high protein accumulation suggests the activation of additional protein types such as the defense proteins and enzymes, during UV treatment. These defense proteins could be an adaptive response to protect the organism against UV radiation. The increase in the protein of the UV-exposed microalgae in this study correlates with the report of Kumar et al. (2018), who observed a 23% protein content for *C. sorokiniana* after exposure to UV radiation. Similarly, El-Sheekh et al. (2021) reported a twofold increase in the protein content of *Microcystis* after UV exposure. In addition, Colina et al. (2020) reported that the exposure of microalgae to UV radiation elevates microalgae adaptive mechanisms and the accumulation of biomolecules such as lipids, proteins, chlorophyll, and carbohydrates associated with the defensive mechanism.

The carbohydrate (CHO) accumulation by the wild *S. vacuolatus* and the 24 h UV-exposed *S. vacuolatus* is depicted in Fig. 6b. The result revealed that the total carbohydrate obtained for the wild-type increased from 1.017 ± 0.001 to 5.345 ± 0.016 g/L after 21 days of cultivation period. While the total CHO content obtained with UV-exposed *S. vacuolatus* increased significantly (p < 0.001) from 6.039 ± 0.008 g/L (day 3) to 24.707 ± 0.006 g/L (day 18), followed by a decline. This sharp decline in the CHO accumulation observed with the UV-exposed *S. vacuolatus*, could be an indication of rapid depletion of nutrient due to rapid growth. The 24.707 g/L CHO content observed for the UV-exposed *S. vacuolatus* is 4.62-fold higher in carbohydrate content compared to the wild *S. vacuolatus*. Moreover, the fraction of biomass to CHO is substantially higher with the UV-exposed *S. vacuolatus* (1:48) in comparison with fraction obtained with the wild-type *S. vacuolatus* (1:20). This further support the effect of UV treatment on the build-up of biomolecules such as CHO in *S. vacuolatus*. This increase in CHO is consistent with El-Sheekh et al. (2021) report, who also observed an increase in total CHO accumulation of *S. acutus* after a 24 h exposure to UV radiation.

Figure 6c depicts the significant difference (p < 0.001) of total lipid obtained in *S. vacuolatus* after UV exposure. The lipid build-up of the UV-exposed *S. vacuolatus* was 0.0514 ± 0.012 g/mL/day and 1.40-fold higher than the wild-type 0.0368 ± 0.015 g/mL/day. This result shows UV radiation's impact on the lipid biosynthesis metabolic pathway (Sivaramakrishnan and Incharoensakdi 2017). Carino and Vital (2022) and Choi et al. (2017) also reported a similar increase in lipid accumulation in *C. vulgaris* after UV exposure. The increase in protein, CHO and lipid contents observed in this study show the potential of UV radiation to enhance *S. vacuolatus* cellular biomolecules to improve cellular activities.

### UV exposed *S. vacuolatus* growth kinetics

**Fig. 7:**
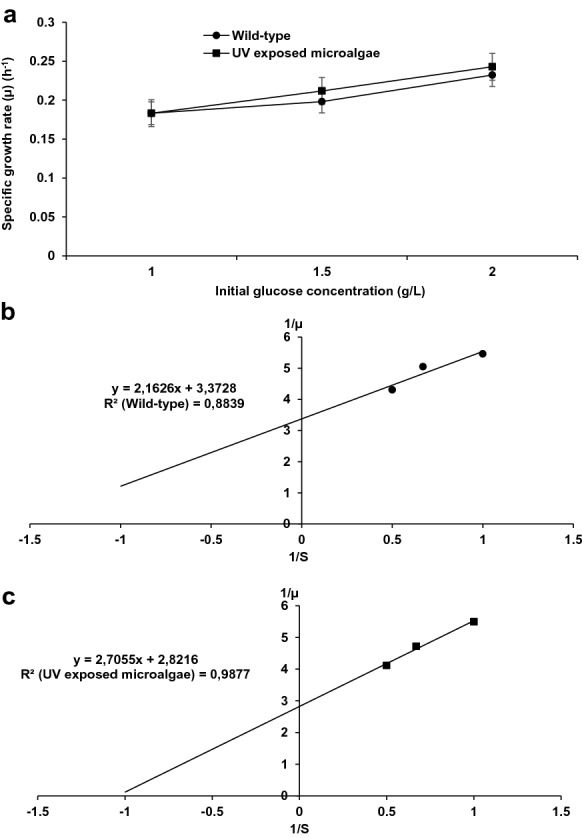
Effect of UV radiation on *S. vacuolatus* growth. **a** Wild-type and UV-exposed microalgae *s*pecific growth rate*s* (μ) at varied initial glucose concentrations increases with increase in initial glucose concentration*.* The specific growth rate of the UV-exposed microalgae was higher compared to the wild-type. **b**, and **c** Lineweaver–Burk plot estimating Monod constants for wild-type and UV exposed microalgae using glucose as substrate. Mean ± standard error of three replicates (n = 3)

The specific growth rate of 24 h UV-exposed *S. vacuolatus* was obtained from the log phase of *S. vacuolatus* growth. The obtained specific growth rate (*µ*) was 0.183, 0.212 and 0.243 day^−1^ at initial substrate concentrations of 1.00, 1.50 and 2.00 g/L, respectively. Alternatively, lower specific growth rate values of 0.183 day^−1^ (1.00 g/L), 0.198 day^−1^ (1.50 g/L) and 0.232 day^−1^ (2.00 g/L) were obtained with the wild *S. vacuolatus* (Fig. 7a). These outcomes suggest the observed µ increases with increasing substrate concentration and could continue until substrate saturation is reached (Okpokwasili and Nweke 2006). Moreover, the higher µ values achieved with the 24 h UV-exposed *S. vacuolatus* suggest the impact of UV radiation on substrate uptake and growth of *S. vacuolatus* (Choi et al. 2017; Ganapathy et al. 2017; Kumar et al. 2018). Microbial engineering developments such as the use of UV radiation and different cloning techniques for inducing improved genetic changes at cellular levels to simultaneously obtained enhanced traits such as shorter lag phase, improved growth rate and metabolic activities have been reported (Choi et al. 2017; Ganapathy et al. 2017; Kumar et al. 2018). Furthermore, taking into account the aforementioned impact of UV radiation on *S. vacuolatus* such as enhanced photosynthetic features and increased growth rate that could have improved *S. vacuolatus* substrate uptake, utilization and improved specific growth rate in the 24 h UV-exposed *S. vacuolatus* was expected.

The estimated Monod constant (*K*_*S*_) and maximum specific growth rate (µ_max_) from the specific growth rate values and initial substrate concentrations are shown in Fig. 7b and c. A maximum specific growth rate (*µ*_*max*_) value of 3.30 day^−1^ and 2.85 day^−1^ were observed for the 24 h UV exposed *S. vacuolatus* and wild-type, respectively. The Ks value obtained for UV-exposed *S. vacuolatus* was 1.56 g/L, while the Ks value of 1.043 g/L was obtained for the wild-type. In addition, the UV-exposed *S. vacuolatus* had a higher affinity constant (1/Ks) for the available substrate (0.959) compared to the wild-type (0.641). The affinity for the substrate by the UV-exposed *S. vacuolatus* was 1.50 times higher than the wild *S. vacuolatus*. The higher µ_max_ and 1/*K*_*s*_ values observed for the 24 h UV-exposed *S. vacuolatus* demonstrate the suitability of UV radiation as a physical agent to improve *S. vacuolatus* substrate uptake and metabolic activities.

The experimental data from the biomass concentration over the cultivation period were used to fit the logistic model with correlation coefficients (*R*^2^) > 0.979. Higher maximum biomass concentration (*X*_*max*_ = 0.511 g/L) was obtained with 24 h UV-exposed *S. vacuolatus* in comparison with wild *S. vacuolatus* (*X*_*max*_ = 0.268 g/L). On the other hand, the wild-type had a higher maximum specific growth rate (µ_max_ = 0.556 day^−1^) than that obtained with 24 h UV-exposed *S. vacuolatus* (µ_max_ = 0.365 day^−1^). Though lower µ_max_ was obtained with 24 h UV-exposed *S. vacuolatus*, higher substrate affinity and biodegradation potential were observed in contrast with the wild-type. Therefore, this suggests that the exposure of *S. vacuolatus* to UV radiation largely enhanced *S. vacuolatus* affinity for the substrate and subsequently enhanced the degradation of SCW to harmless by-products.

Furthermore, the kinetic growth constant (k_g_) (0.1615 day^−1^) was obtained for the UV exposed *S. vacuolatus* during the SLW degradation process (see supplementary document). This was 1.27-fold high compared to wild *S. vacuolatus* (0.1274 day^−1^) under the same experimental condition. Interestingly, the high k_g_ obtained with UV exposed *S. vacuolatus* corroborate with the high substrate affinity, specific growth rate and high biomass accumulation also observed with the UV exposed *S. vacuolatus*. This could be attributed to the positive impact of UV radiation on *Scenedesmus vacuolatus* photosynthetic apparatus, production of enzymes and other physiological cellular behaviour to improve cellular growth and simultaneously degrade the lubricant oil waste.

### *Scenedesmus vacuolatus* substrate usage versatility

**Fig. 8:**
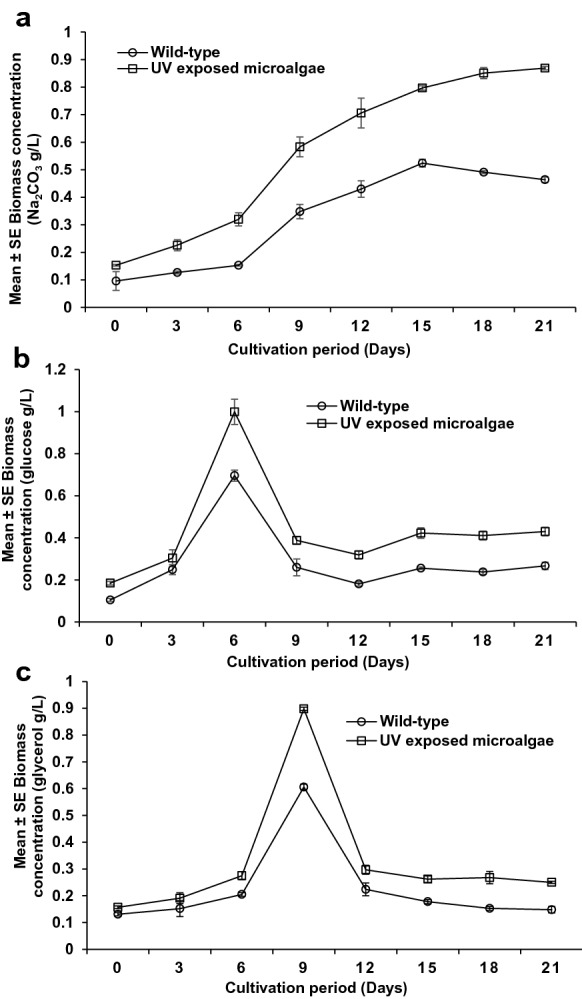
The cultivation of wild-type microalgae and UV exposed *S. vacuolatus* using different substrate. **a** Sodium carbonate NaCO2; **b** glucose; **c** glycerol. Mean ± standard error of three replicates (n = 3)

Figure 8 illustrates the variation in the growth rate of the 24 h UV-exposed *S. vacuolatus* cultivated with sodium carbonate (Na_2_CO_3_), glucose and glycerol as the only carbon source. The results obtained revealed *S. vacuolatus* showed a significant difference in growth pattern with the studied substrates (Na_2_CO_3_, glucose and glycerol) as the sole carbon source. These variations in growth patterns may be attributed to the differences in the chemical structural complexity of the compounds. Also, the variations in the specific growth rates demonstrate variations in the metabolism of the different substrates by *S. vacuolatus* (Danesh et al. 2019; Rambhiya1 et al. 2021). *S. vacuolatus* showed the highest growth rate and biomass concentration with glucose as the sole carbon source. This suggests that glucose is the most preferred carbon source by *S. vacuolatus* of the three substrates, probably due to its simple carbon–carbon bond (Danesh et al. 2019). Moreover, higher specific growth rates were observed in all the studied substrates with 24 h UV-exposed *S. vacuolatus* compared to the wild-type (Fig. 8a–c). The maximum specific growth rates of 0.054, 0.136 and 0.082 day^−1^, were observed for the UV-exposed *S. vacuolatus* compared to that 0.028, 0.099 and 0.053 day^−1^ obtained for Na_2_CO_3_, glucose and glycerol of the wild *S. vacuolatus*, respectively. The UV-exposed *S. vacuolatus* also showed significantly increased biomass concentration up to 0.869 ± 0.006 g/L, 0.999 ± 0.060 g/L, 0.898 ± 0.006 g/L for Na_2_CO_3_, glucose and glycerol, respectively. The attained biomass concentration was 1.67, 1.44 and 1.48 times higher than those obtained with the wild-type (0.524 ± 0.014 g/L, 0.696 ± 0.026 g/L, 0.606 ± 0.011 g/L). The higher specific growth rates and biomass concentrations obtained by the UV-exposed *S. vacuolatus* can be ascribed to enhanced adaptability, metabolic capacity and substrate consumption versatility.

### Effect of UV radiation on *S. vacuolatus* biodegradation potential

**Fig. 9:**
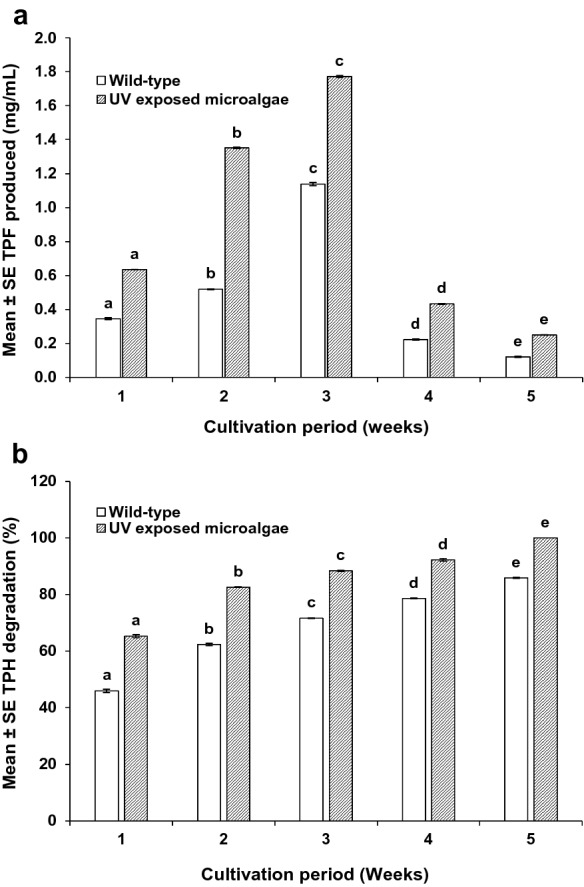
The dehydrogenase activity and total petroleum hydrocarbon degradation of spent coolant waste after 5 weeks. **a** TPF production by *S. vacuolatus*, **b** TPH degradation by *S. vacuolatus*. The share letters (a, b, c, d and e) show that the TPF and TPH at different weeks are significantly different for each microalgae strain. Mean ± standard error of six replicates (n = 6)

To determine the potential of waste lubricant of biodegradation by the 24 h UV-exposed *S. vacuolatus*, a preliminary assessment for spent coolant waste (SCW) biodegradation was carried out. The DHA production and TPH degradation of SCW after 5 weeks is presented in Fig. 9a and b (The TPF produced and gas chromatogram of the TPH degradation are shown in the supplementary document). The results showed the 24 h UV-exposed *S. vacuolatus* has significantly higher (p < 0.001) DHA and TPH degradation efficiency in comparison with the wild *S. vacuolatus*. The highest DHA with TPF production of 1.771 ± 0.006 mg/mL was obtained at week 3, while the highest TPH degradation (100%) was obtained at week 5. These were 36% and 14% improvements over the DHA and the TPH obtained with the wild*-*type. The increment in DHA and TPH degradation might also be attributed to enhanced photosynthetic and metabolic activities of UV-exposed *S. vacuolatus* resulting from improved cellular activities after the UV exposure (Choi et al. 2017; Ganapathy et al. 2017; Kumar et al. 2018). Previous studies by Megharaj et al. (2000) and Eregie and Jamal (2019, 2021) also reported an increase in DHA and TPH degradation of HCs using the *Chlorella* and *Scenedesmus* sp, respectively*.*

The HCs detected in the SCW sample after treatment with the 24 h UV-exposed *S. vacuolatus* are shown in the supplementary document. A relatively high biodegradation of HCs of SCW was observed compared to the control. The possible reasons for the high degradation of HCs after 5 weeks of treatment include (1) high tolerance and metabolism of SCW as substrate, (2) increased enzymatic metabolic activities that improved the metabolic activities towards SCW degradation (Ibrahim 2016; Ichor et al. 2016; Medic et al. 2020). The different alkanes (C8 to C43) present in the control (see supplementary document) were degraded by the UV-exposed *S. vacuolatus* into environmentally friendly products such as alkanols, alcohols, ketones, aldehydes, fatty acids, and carboxylic acids. Similar degradation products were also observed in the SCW treated with the wild-type (see supplementary document). Wild-type and 24 h UV-exposed *S. vacuolatus* probably exhibited a subterminal oxidation pathway for the degradation of alkanes (Patowary et al. 2017; Medic et al. 2020). Subterminal oxidation of alkane HCs have also been reported, resulting in the formation of an alcohol that is then hydroxylated to form ketone and ester by-products. The ester formed is further oxidized to produce fatty acid (Kumar et al. 2014; Patowary et al. 2017). Moreover, mono and polycyclic aromatics were degraded to their harmless degradation products by UV-exposed *S. vacuolatus* within the first week of SCW treatment compared to the control and the wild-type that occurred in subsequent weeks. A suggestive of rapid utilization of SCW as substrate resulting from high photosynthetic and metabolic activities (Patowary et al. 2016; Choi et al. 2017; Żyszka-Haberecht et al. 2019). The ability of the UV-exposed *S. vacuolatus* to degrade the aromatics to metabolic products that are non-toxic within the first week of treatment is significant and could be of biotechnological importance in the biodegradation of SCW, oil waste, or spillage. After 5 weeks of SCW treatment by 24 h UV-exposed *S. vacuolatus*, no HCs nor their degradation products (signifying complete metabolism/degradation) were detected compared to the control and degradation profiles obtained from the wild *S. vacuolatus* experiments where undegraded HCs were detected after 5 weeks. The results obtained further demonstrate the enhanced biodegradation potential of the UV-exposed *S. vacuolatus*.

## Conclusion

This study demonstrates the impact of UV light radiation on the metabolic performance and biodegradation potential of *S. vacuolatus*. Higher growth rate, substrate affinity, chlorophyll, protein, carbohydrate, and lipid accumulation were obtained with the 24 h UV-exposed *S. vacuolatus*. Moreover, the exposure of *S. vacuolatus* to UV radiation enhanced the DHA by 1.55-fold and biodegradation efficiency by 100% with SCW as substrate. This study substantiates the potential of microalgae engineering to improve waste biodegradation towards eco-friendly environmental sustainability.

### Supplementary Information

Below is the link to the electronic supplementary material.

Supplementary file1 (PDF 1707 KB)

Supplementary file2 (DOCX 51498 KB)

## Data Availability

I Stella Eregie confirm that this work is original and has not been published elsewhere, nor is it currently under consideration for publication elsewhere. Opinions expressed, and conclusions arrived at, are those of the authors and are not necessarily to be attributed to the NRF. All the data analyzed during this study are included in this research article and its supplementary documents.

## References

[CR1] Akgül F, Kizilkaya IT, Akgul R, Erdugan H (2017). Morphological and molecular characterization of *Scenedesmus*-like species from Ergene river basin (Thrace, Turkey). Turk J Fish Aquat Sci.

[CR2] Arora N, Yen HW, Philippidis GP (2020). Harnessing the power of mutagenesis and adaptive laboratory evolution for high lipid production by oleaginous microalgae and yeasts. Sustainability.

[CR3] Asgari A, Nabizadeh R, Mahvi AH, Nasseri S, Dehghani MH, Nazmara S, Yaghmaeian K (2017). Biodegradation of total petroleum hydrocarbons from acidic sludge produced by re-refinery industries of waste oil using in-vessel composting. J Environ Health Sci Eng.

[CR4] Baghour M (2019) Algal degradation of organic pollutants. Handbook of Eco-materials. Springer, Cham. pp 1–22

[CR5] Beherepatil KH, Deore LT (2013). Genus *Scenedesmus* from different habitats of Nashik and its environs (MS) India. Int J Bioassays.

[CR6] Bligh EG, Dyer WJ (1959). A rapid method of total lipid extraction and purification. Can J Biochem Physiol.

[CR7] Bradford MM (1976). A rapid and sensitive method for the quantitation of microgram quantities of protein utilizing the principle of protein-dye binding. Anal Biochem.

[CR8] Carino JD, Vital PG (2022). Characterization of isolated UV-C-irradiated mutants of microalga *Chlorella vulgaris* for future biofuel application. Environ Dev Sustain.

[CR9] Choi TO, Kim KH, Kim GD, Choi TJ, Jeon YJ (2017). The evaluation of UV-induced mutation of the Microalgae, *Chlorella vulgaris* in mass production systems. J Life Sci.

[CR10] Colina F, Carbó M, Meijón M, Cañal MJ, Valledor L (2020). Low UV-C stress modulates *Chlamydomonas reinhardtii* biomass composition and oxidative stress response through proteomic and metabolomic changes involving novel signalers and effectors. Biotechnol Biofuels.

[CR11] Danesh A, Zilouei H, Farhadian O (2019). The effect of glycerol and carbonate on the growth and lipid production of *Isochrysis galbana* under different cultivation modes. J Appl Phycol.

[CR12] Dell’Anno F, Rastelli E, Sansone C, Brunet C, Ianora A, Dell’Anno A (2021). Bacteria, fungi and microalgae for the bioremediation of marine sediments contaminated by petroleum hydrocarbons in the omics era. Microorganisms.

[CR59] Ding A, Sun Y, Dou J, Cheng L, Jiang L, Zhang D, Zhao X (2013) Characterizing microbial activity and diversity of hydrocarbon contaminated sites. Hydrocarbon 16:137. 10.5772/50480

[CR13] El-Sheekh MM, Hamouda RA, Nizam AA (2013). Biodegradation of crude oil by *Scenedesmus obliquus* and *Chlorella vulgaris* growing under heterotrophic conditions. Int Biodeterior Biodegrad.

[CR14] El-Sheekh MM, Alwaleed EA, Ibrahim A, Saber H (2021). Detrimental effect of UV-B radiation on growth, photosynthetic pigments, metabolites and ultrastructure of some *cyanobacteria* and freshwater *chlorophyta*. Int J Rad Biol.

[CR15] Eregie SB, Jamal-Ally SF (2019). Comparison of biodegradation of lubricant wastes by *Scenedesmus vacuolatus* vs a microalgal consortium. Bioremed J.

[CR16] Eregie SB, Jamal-Ally SF (2021). Comparison of biodegradative efficiency of wild-type versus mutagenised *Scenedesmus vacuolatus* of spent coolant waste: dehydrogenase activity and total petroleum degradation studies. Int J Environ Anal Chem.

[CR17] Fajardo C, De Donato M, Carrasco R, Martínez-Rodríguez G, Mancera JM, Fernández-Acero FJ (2020). Advances and challenges in genetic engineering of microalgae. Rev Aquacult.

[CR18] Fu A, Chaiboonchoe B, Khraiwesh DR, Nelson D, Al-Khairy A, Mystikou S-A (2016). Algal cell factories: approaches, applications, and potentials. Mar Drugs.

[CR19] Ganapathy K, Chidambaram K, Janarthanan R, Ramasamy R (2017). Effect of UV-B radiation on growth, photosynthetic activity and metabolic activities of *Chlorella vulgaris*. J Microbiol Biotechnol.

[CR20] Gonçalves AL (2021). The use of microalgae and cyanobacteria in the improvement of agricultural practices: a review on their biofertilising, biostimulating and biopesticide roles. Appl Sci.

[CR57] Hlavova MZ Turoczy, Bisova K (2015) Improving microalgae for biotechnology-From genetics to synthetic biology. Biotechnol Adv 33:1194–1203. 10.1016/j.biotechadv.2015.01.009 10.1016/j.biotechadv.2015.01.009 25656099

[CR21] Ibrahim HM (2016). Biodegradation of used engine oil by novel strains of *Ochrobactrum anthropi* HM-1 and *Citrobacter freundii* HM-2 isolated from oil-contaminated soil. 3 Biotech.

[CR22] Ichor T, Okerentugba PO, Okpokwasili GC (2016). Biodegradation of total petroleum hydrocarbon by a consortium of cyanobacteria isolated from crude oil polluted brackish waters of bodo creeks in Ogoniland. Rivers State Res J Environ Toxicol.

[CR58] Irawan C, Sulistiawaty L, Sukiman M (2018) Volatile compound analysis using GC-MS, phytochemical screening, and antioxidant activities of the Husk of Julang-Jaling (*Archidendron bubalinum* (Jack) IC Nielsen) from Lampung, Indonesia. Pharmacogn J 10. 10.5530/pj.2018.1.17

[CR23] Ismail MM, Ismail GA, El-Sheekh MM (2020). Potential assessment of some micro-and macroalgal species for bioethanol and biodiesel production. Energy Sources A.

[CR24] Kajdas C, Mang T (2014). Used oil disposal and collection. Encyclopedia of lubricants and lubrication.

[CR25] Kultschar BE, Dudley S, Wilson LCA (2019). Intracellular and extracellular metabolites from the cyanobacterium *chlorogloeopsis fritschii*, 6912, during 48 hours of UV-B exposure. Metabolites.

[CR26] Kumar AG, Vijayakumar L, Joshi G, Peter DM, Dharani G, Kirubagaran R (2014). Biodegradation of complex hydrocarbons in spent engine oil by novel bacterial consortium isolated from deep sea sediment. Biores Technol.

[CR27] Kumar NM, Muthukumaran C, Sharmila G, Gurunathan B (2018). Genetically modified organisms and its impact on the enhancement of bioremediation. Bioremediation.

[CR28] Kumar G, Shekh A, Jakhu S, Sharma Y, Kapoor R, Sharma TR (2020). Bioengineering of microalgae: recent advances, perspectives, and regulatory challenges for industrial application. Front Bioeng Biotechnol.

[CR29] Lim DK, Schenk PM (2017). Microalgae selection and improvement as oil crops: GM vs non-GM strain engineering. AIMS Bioeng.

[CR30] Liu S, Zhao Y, Liu L, Ao X, Ma L, Wu M, Ma F (2015). Improving cell growth and lipid accumulation in green microalgae *Chlorella* sp. via UV irradiation. Appl Biochem Biotechnol.

[CR31] Medić A, Lješević M, Inui H, Beškoski V, Kojić I, Stojanović K, Karadžić I (2020). Efficient biodegradation of petroleum n-alkanes and polycyclic aromatic hydrocarbons by polyextremophilic *Pseudomonas aeruginosa* san ai with multidegradative capacity. RSC Adv.

[CR60] Megharaj M, Singleton I, McClure NC, Naidu R (2000) Influence of petroleum hydrocarbon contamination on microalgae and microbial activities in a long-term contaminated soil. Arch Environ Contam Toxicol 38: 439–445. 10.1007/s002449910058 10.1007/s002449910058 10787094

[CR32] Miller GL (1959). Use of dinitrosalicylic acid reagent for determination of reducing sugar. Anal Chem.

[CR33] Moha-León JD, Pérez-Legaspi IA, Ortega-Clemente LA, Rubio-Franchini I, Ríos-Leal E (2019). Improving the lipid content of *Nannochloropsis oculata* by a mutation-selection program using UV radiation and quizalofop. J Appl Phycol.

[CR34] Okpokwasili GC, Nweke CO (2006). Microbial growth and substrate utilization kinetics. Afr J Biotechnol.

[CR35] Patowary K, Patowary R, Kalita MC, Deka S (2016). Development of an efficient bacterial consortium for the potential remediation of hydrocarbons from contaminated sites. Front Microbiol.

[CR36] Patowary K, Patowary R, Kalita MC, Deka S (2017). Characterization of biosurfactant produced during degradation of hydrocarbons using crude oil as sole source of carbon. Front Microbiol.

[CR37] Pessoa MF (2012). Harmful effects of UV radiation in algae and aquatic macrophytes-a review. Emirates J Food Agric.

[CR38] Radziff SBM, Ahmad SA, Shaharuddin NA, Merican F, Kok YY, Zulkharnain A, Wong CY (2021). Potential application of algae in biodegradation of phenol: a review and bibliometric study. Plants.

[CR39] Rambhiya SJ, Magar CS, Deodhar MA (2021). Using seawater-based Na_2_CO_3_ medium for scrubbing the CO_2_ released from Bio-CNG plant for enhanced biomass production of *Pseudanabaena limnetica*. SN Appl Sci.

[CR40] Rastogi RP, Madamwar D, Nakamoto H, Incharoensakdi A (2020). Resilience and self-regulation processes of microalgae under UV radiation stress. J Photochem Photobiol.

[CR41] Rathi V, Yadav V (2019). Oil degradation taking microbial help and bioremediation: a review. J Bioremed Biodegrad.

[CR42] Saha RC, Reza A, Hasan MS, Saha P (2019) A review-bioremediation of oil sludge contaminated soil. In E3S *Web of Conferences* 96:01004. EDP Sciences. 10.1051/e3sconf/20199601004

[CR43] Sanusi IA, Suinyuy TN, Lateef A, Kana GE (2020). Effect of nickel oxide nanoparticles on bioethanol production: process optimization, kinetic and metabolic studies. Process Biochem.

[CR44] Sarayloo E, Simsek S, Unlu YS, Cevahir G, Erkey C, Kavakli IH (2018). Enhancement of the lipid productivity and fatty acid methyl ester profile of *Chlorella vulgaris* by two rounds of mutagenesis. Biores Technol.

[CR45] Sarwa P, Verma SK (2017). Identification and characterization of green microalgae, *Scenedesmus* sp. MCC26 and *Acutodesmus obliquus* MCC33 isolated from industrial polluted site using morphological and molecular markers. Int J Appl Sci Biotechnol.

[CR46] Savvidou MG, Dardavila MM, Georgiopoulou I, Louli V, Stamatis H, Kekos D, Voutsas E (2021). Optimization of microalga *Chlorella vulgaris* magnetic harvesting. Nanomater.

[CR47] Sihag S, Pathak H (2016) Biodegradation of 2T engine oil using soil microbe and gravimetric analysis. Int J Sci Eng Res

[CR48] Sivaramakrishnan R, Incharoensakdi A (2017). Enhancement of lipid production in *Scenedesmus* sp. by UV mutagenesis and hydrogen peroxide treatment. Biores Technol.

[CR49] Subashchandrabose SRB, Ramakrishnan M, Megharaj K, Venkateswarlu NR (2013). Mixotrophic cyanobacteria and microalgae as distinctive biological agents for organic pollutant degradation. Environ Int.

[CR50] Sydney T, Marshall-Thompson JA, Kapoore RV, Vaidyanathan S, Pandhal J, Fairclough JPA (2018). The effect of high-intensity ultraviolet light to elicit microalgal cell lysis and enhance lipid extraction. Metabolites.

[CR51] Tenorio C, Ramírez JAH, Ramos LF, Soto AR, Vargas J (2022). Effects of ultraviolet radiation on production of photoprotective compounds in microalgae of the genus *Pediastrum* from high Andean areas of Peru. J Appl Pharm Sci.

[CR52] Thurakit T, Pumas C, Pathomaree W, Pekkoh J, Peerapornpisal Y (2018). Enhancement of biomass, lipid and hydrocarbon production from green microalga, *Botryococcus braunii* AARL G037, by UV-C induction. Chiang Mai J Sci.

[CR53] Tomar RS, Jajoo A (2021). Enzymatic pathway involved in the degradation of fluoranthene by microalgae *Chlorella vulgaris*. Ecotoxicology.

[CR54] Touliabah HE, El-Sheekh S, Ismail MM, El-Kassas H (2022). A review of microalgae-and cyanobacteria-based biodegradation of organic pollutants. Mole.

[CR56] Żyszka-Haberecht B, Niemczyk E, Lipok J (2019). Metabolic relation of cyanobacteria to aromatic compounds. Appl Microbiol Biotechnol.

