# Supplementary material for: Effect of ultra-violet light radiation on Scenedesmus vacuolatus growth kinetics, metabolic performance, and preliminary biodegradation study
Source: Biodegradation. 2023 Apr 13;35(1):71–86. doi: 10.1007/s10532-023-10029-2 (PMC10774200; doi:10.1007/s10532-023-10029-2)
Supplement: Supplementary file 1 — Supplementary file1 (PDF 1707 KB) [file 10532_2023_10029_MOESM1_ESM.pdf]

Supplementary documents

Effect of ultra-violet light radiation on *Scenedesmus vacuolatus* growth: Process kinetics, metabolic performance, and preliminary biodegradation study

Stella B. Eregie[1]*, Isaac A. Sanusi[1,2], Gueguim E.B. Kana[1], Olaniran O. Ademola[3]

[1]School of Life Sciences, University of KwaZulu-Natal, Private Bag, X0, Pietermaritzburg, South Africa

[2]Fort Hare Institute of Technology, University of Fort Hare, Private Bag X1314, Alice, 5700, South Africa

[3]School of Life Sciences, University of KwaZulu-Natal Private Bag X54001, Westville Campus, South Africa

Supplementary document 1

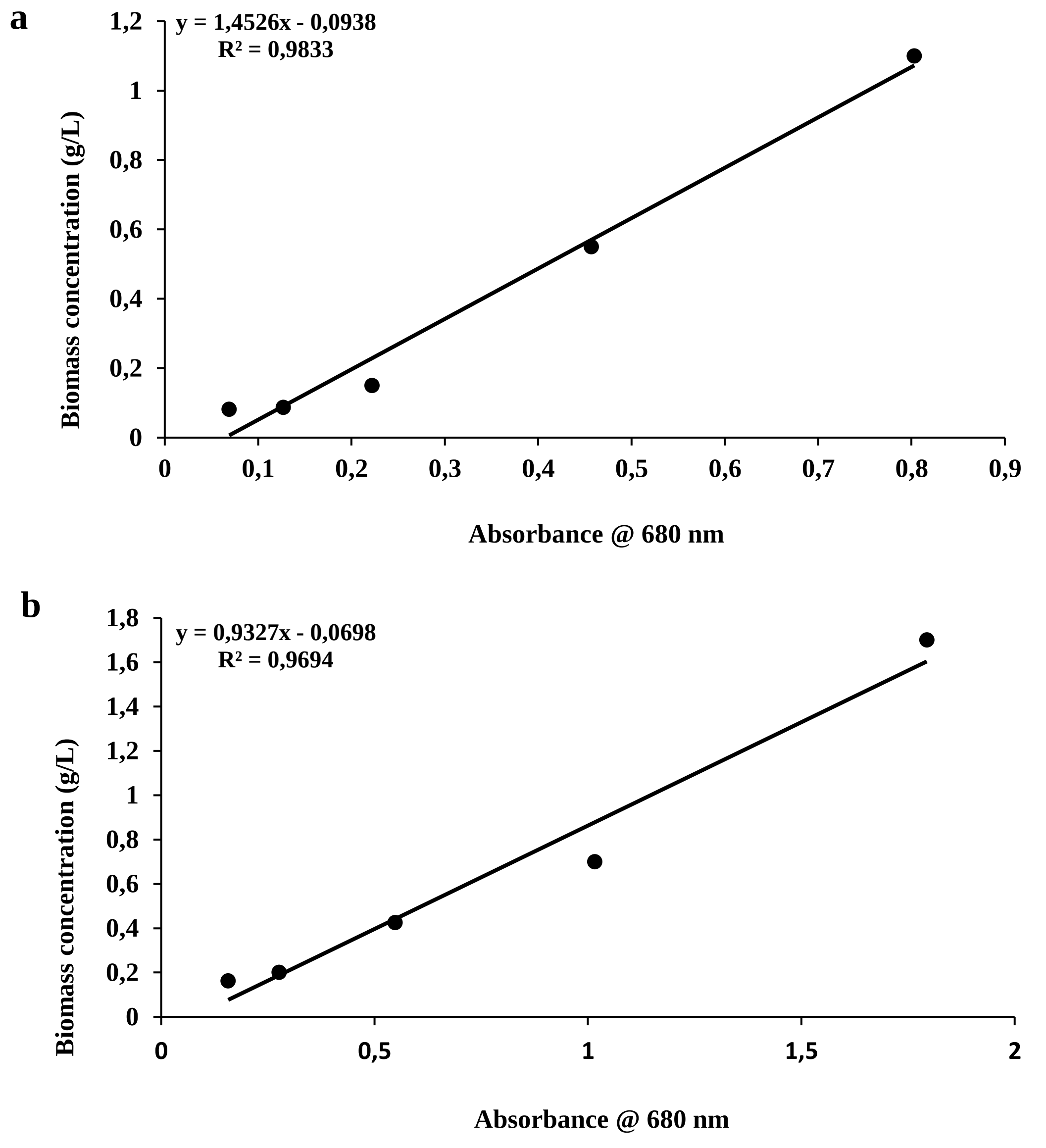

Fig. S1. The standard calibration curves used to calculate (a) standard curve used to calculate wild-type cell dry weight and (b) standard curve used to calculate UV-exposed *Scenedesmus*

Supplementary document 2

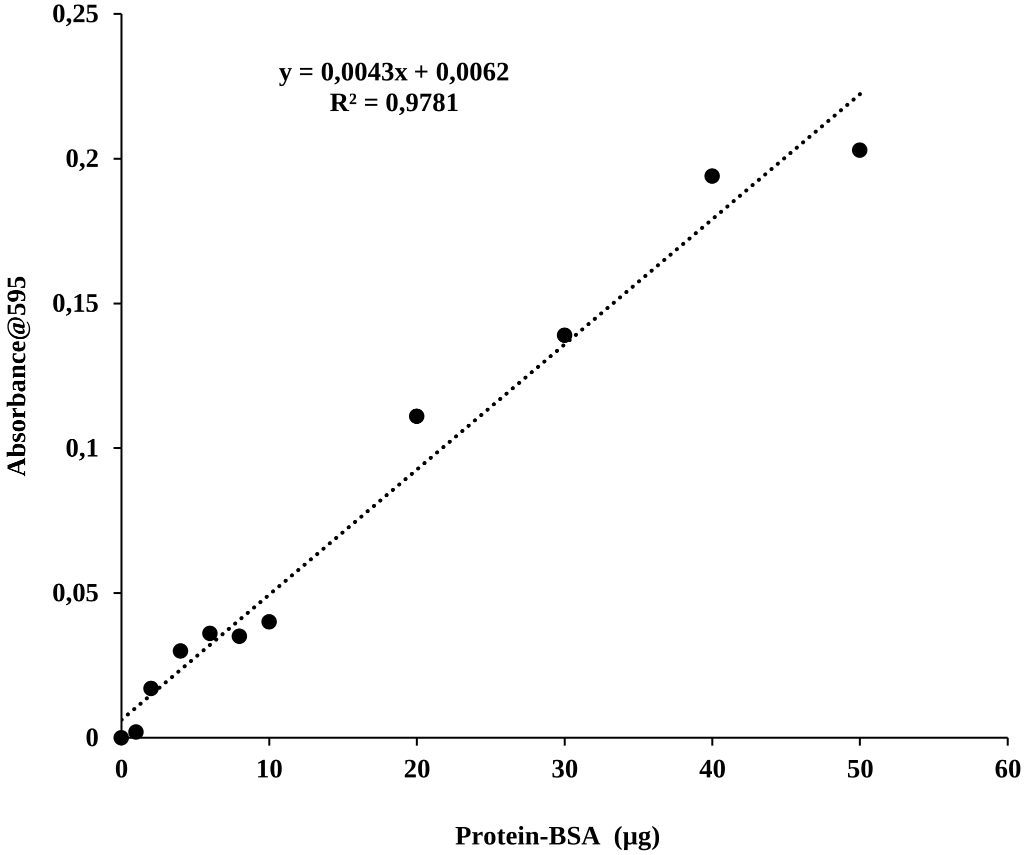

Fig. S2. The standard calibration curve used to deduce protein concentration of microalgae

Supplementary document 3

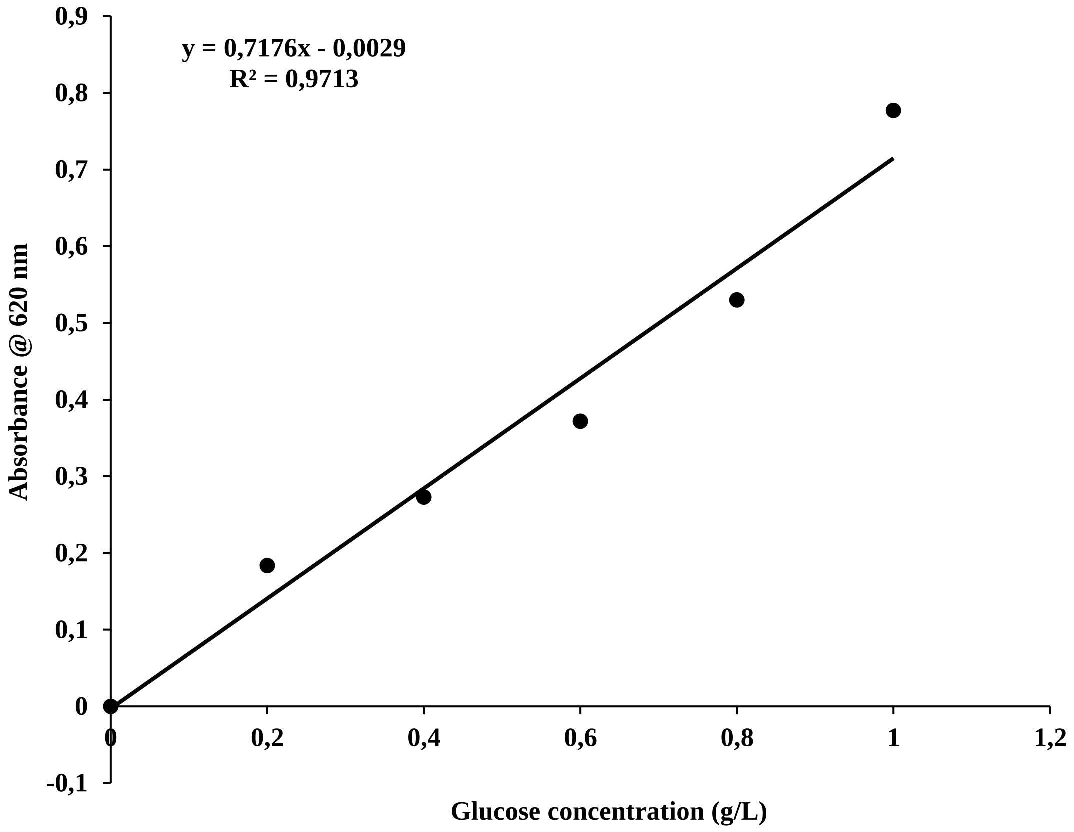

Fig. S3. The standard calibration curve used to deduce carbohydrate accumulation of microalgae

Supplementary document 4

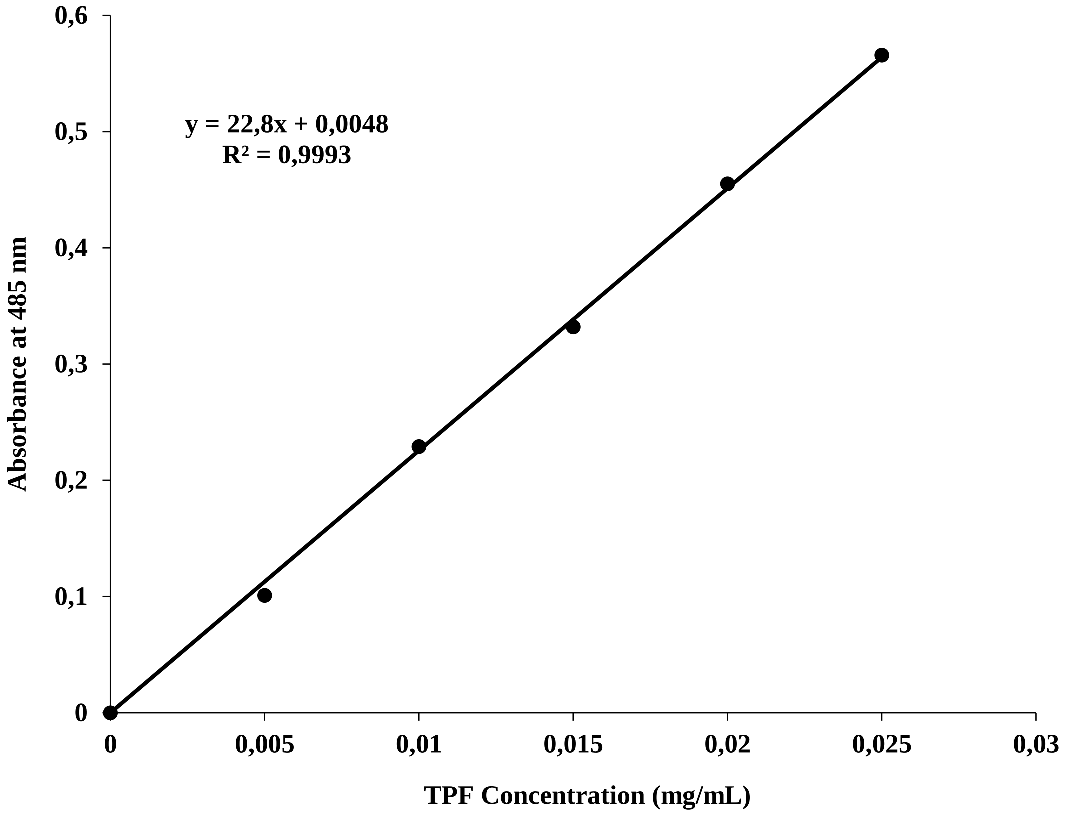

Fig. S4. The Triphenyl formazan (TPF) standard calibration curve used to calculate the unknown concentration of TPF produced.

Supplementary document 5

a

SS

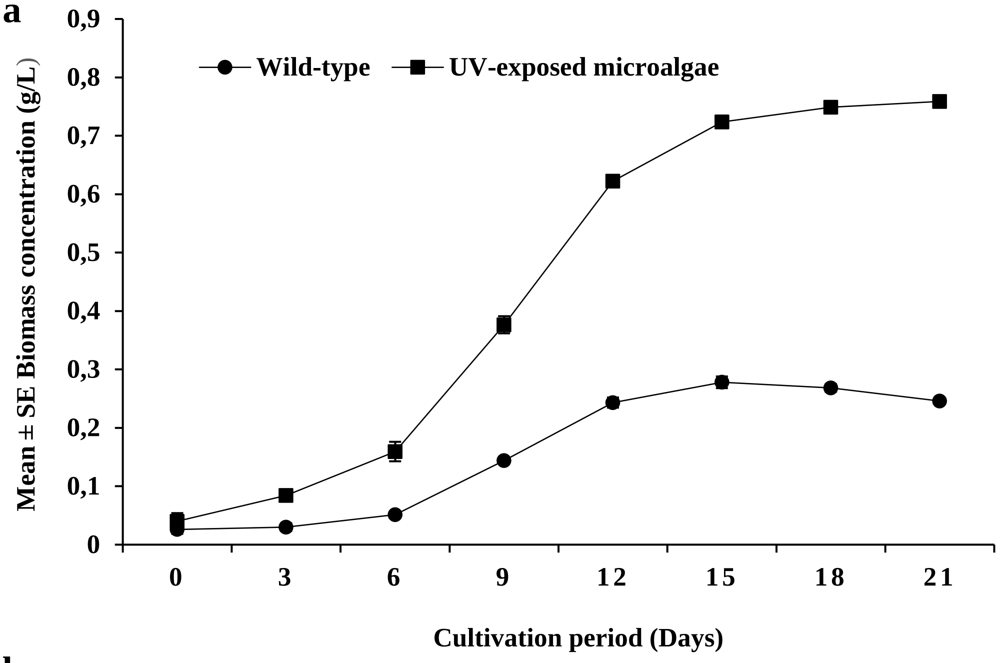

b

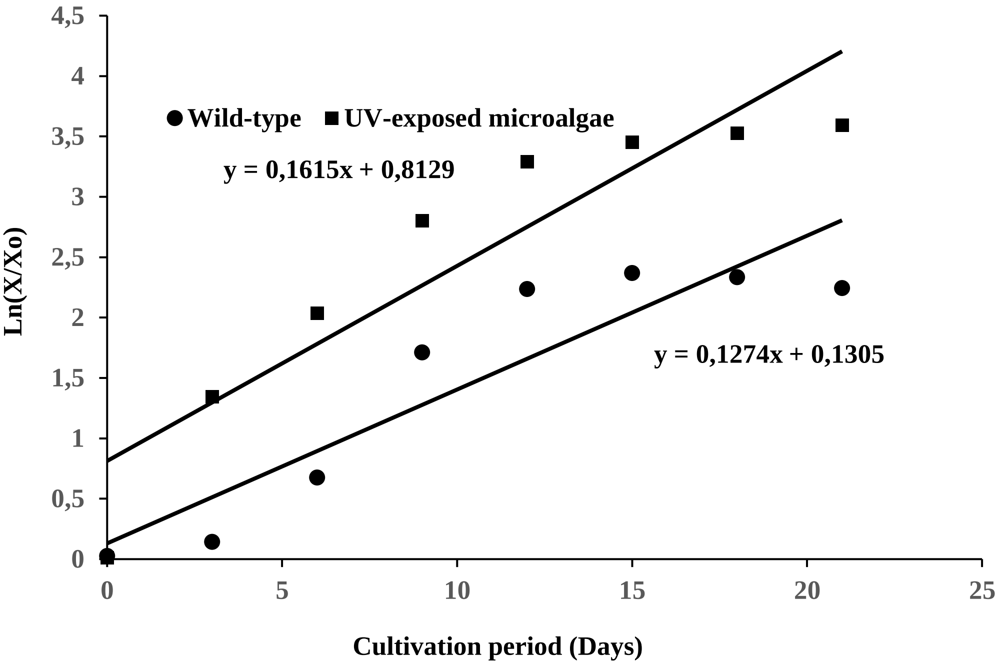

Fig. S5. The biomass concentration and growth constant of microalgae. (a) Biomass concentration obtained for wild-type and UV-exposed microalgae. (b) The growth rate constant of microalgae after 15 days of cultivation. From the result obtained, ln X/Xo increased proportionally with increasing incubation time. The slope measures the rate of growth. In this case the greater the slope the higher the growth rate and biomass accumulation. The UV-exposed microalgae had the highest growth rate and biomass concentration and the steepest slope during the log phase (0.162), compared to the wild-type (0.127).

Supplementary document 6

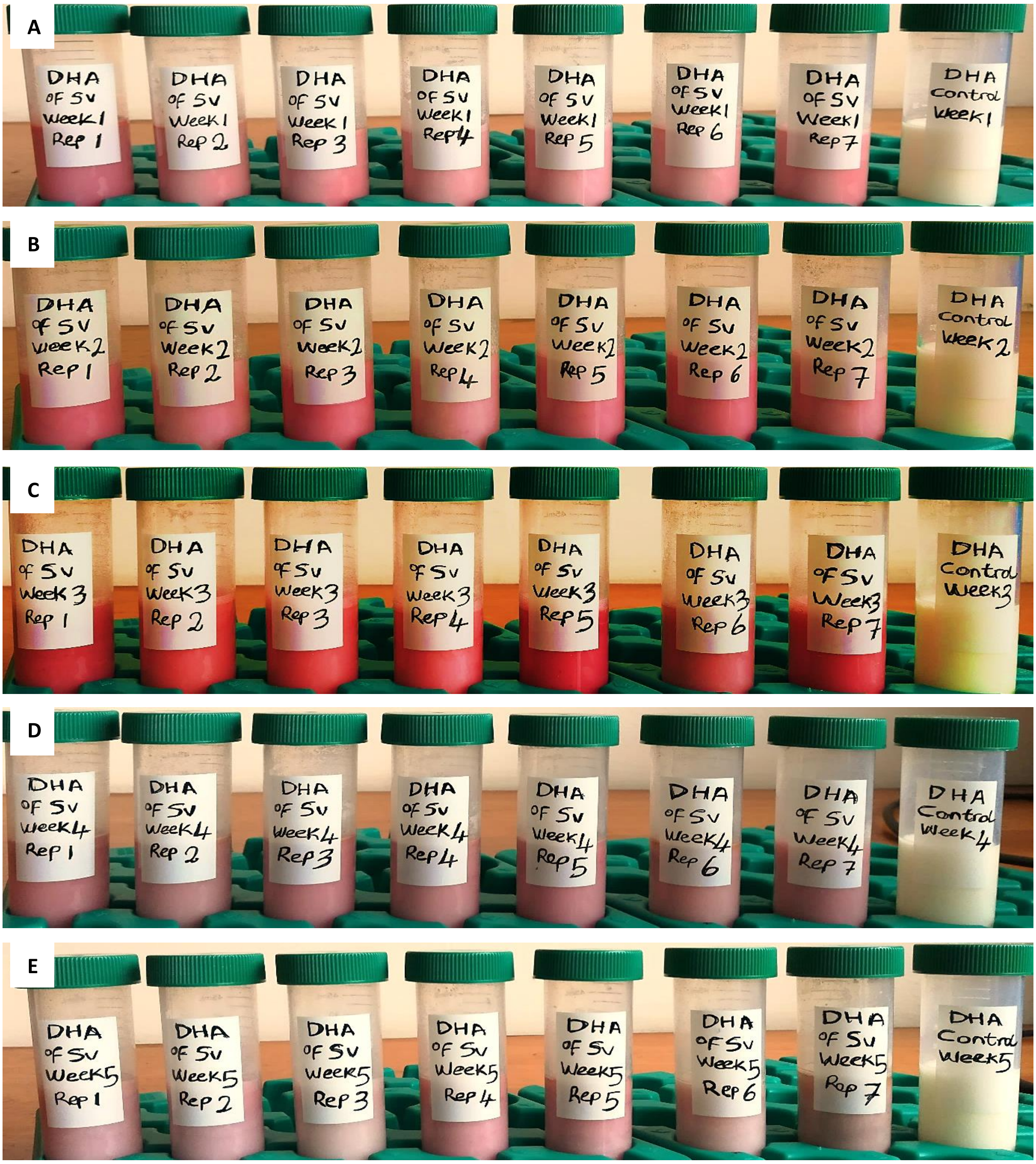

Fig. S6: Dehydrogenase activity of wildtype microalgae in spent coolant waste. The pink color represents the TPF produced. A show a light pink color at week one, followed by B with pink color at week two. C show a dark pink color with high concentrations of TPF at week three. pink color was observed at D week four and week five a pale pink color was observed indicating a decrease in TPF production.

Supplementary document 7

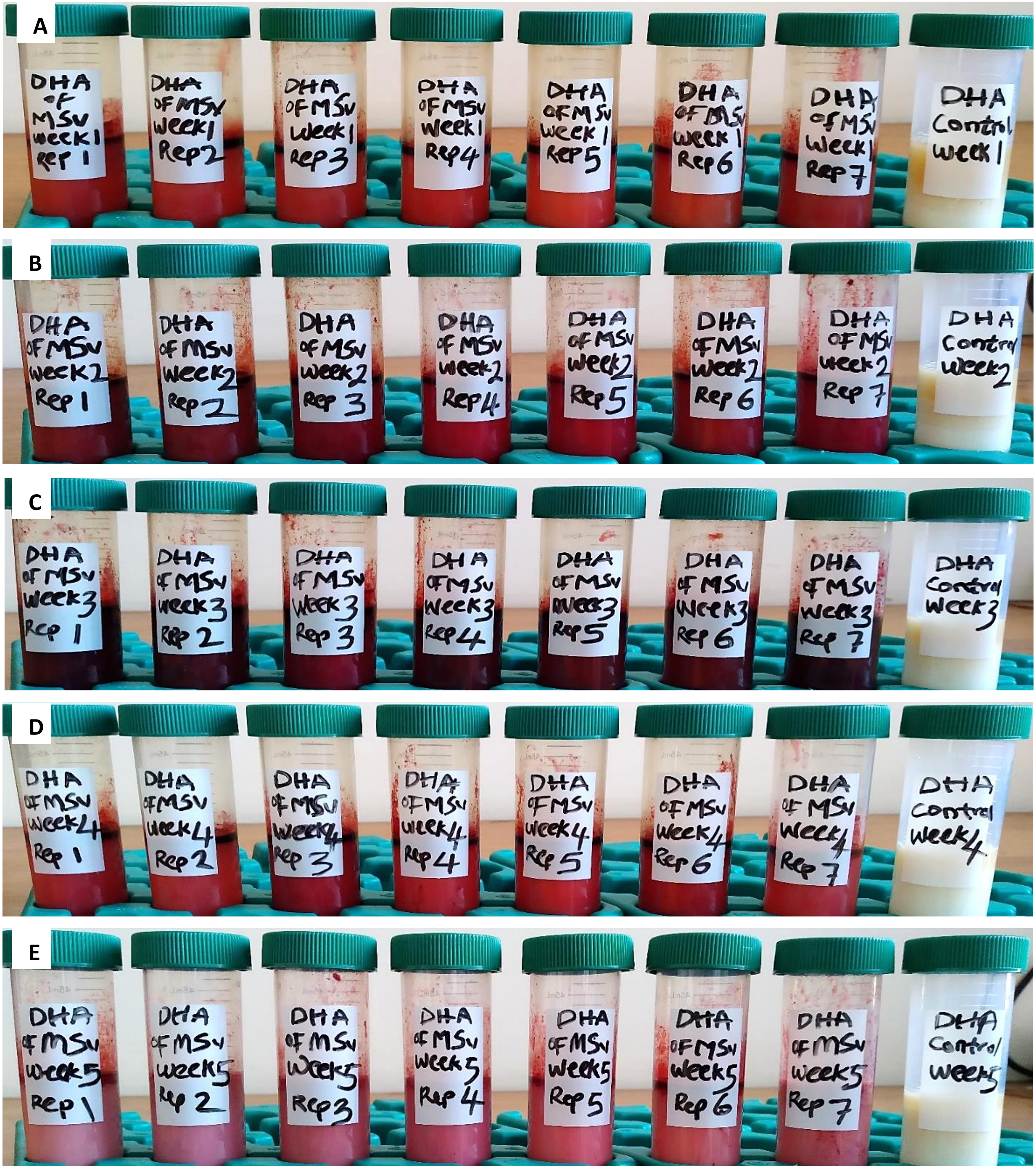

Fig S7: Dehydrogenase activity of UV exposed *S. vacuolatus* in spent coolant waste. The pink color represents the TPF produced. A show a pink color at week one, followed by B with dark pink color with high production of TPF at week two. C show a very dark pink color with higher production of TPF at week three. Pink color was observed at D week four and week five a light pink color was observed indicating a decrease in TPF production.

Supplementary document 8

Table 1: Hydrocarbon compounds present in spent coolant waste (SCW) abiotic control

Alkanes compounds	**Formula/Isomers**		**Alkenes compounds**	**Formula/Isomers**		**Monoaromatic compounds**	**Formula/Isomer**	
Hexane,2,3-dimethyl	C8H18	3	2-Undecene,2,5-dimethyl	C13H26		p-Xylene	C8H10	
Cyclohexane,1,3-dimethyl-cis	C8H16		1-Undecene,7-methyl	C12H24		Benzene,1-ethyl-2-methyl	C9H12	
Cyclopentane,1-ethyl-3-methyl-cis	C8H16		8-Heptadecene,1-chloro-	C17H33Cl		Benzene, (1,3,3-trimethylnonyl)	C18H30	
1-Ethyl-2-(4-methylpentyl) cyclopentane	C13H26		Tricyclo (2,6) undeca-2(6),3-diene,11-methyl	C11H10O2		Benzene,1-ethyl-2,4-dimethyl	C10H14	1
Nonane	C9H20	4	1,3-Cyclopentadiene,1,2,3,4-tetramethyl-5-methylene	C10H14		Benzene,2-(2-butenyl)-1,3,5-trimethyl	C13H18	
1-Ethyl-4-methylcyclohexane	C9H18	1	3-Hexene, 3-ethyl-2,5-dimethyl-					
Decane	C10H22	4	2,4,4,6-Tetramethyl-6-phenyl-1-heptene			4-Ethylbenzylamine, N, N-diheptyl	C23H41N	
Cyclohexane,1,1-dimethyl-2-propyl	C11H22	1	**Organic acids/other compounds**			**Alcohols**		
Undecane	C11H24	4	Sulfurous acid,2-ethylhexylhexadecyl ester	C14H50O3S		1-Undecanol	C11H24O	
Tritetracontane	C43H88		Malonic acid, 2-heptyl tetradecyl ester	C24H46O4		2-Hexyl-1-octanol	C14H30O	
Cyclopentane,1,1'-ethylidenebis	C12H22		m-Toluic acid,2-tetrahydrofurylmethyl ester	C11H14O2		1-Heptanol,2-propyl	C10H22O	
Hexadecane, 1-chloro-			10-Chlorodecyl propyl carbonate			(+-)-trans-1-Isopropenyl-4-methyl-1,4-cyclohexanediol		
Cyclohexane, 1-ethyl-1-methyl-			2-Butyloxycarbonyloxy-1,1,10-trimethyl-6,9-epidioxydecalin			n-Pentadecanol	C15H32O	
Cyclopropane,1-chloro-1-ethyl-2,2,3-trimethyl	C8H15Cl		**PAHs**			1-Hexanol,5-methyl-2-(1-methyl ethyl)	C10H22O	
Dodecane	C12H26	4	Naphthalene,1,2,3,4-tetrahydro-6-methyl	C11H14		**Chlorinated compounds**		
Dodecane,3-cyclohexyl	C18H36		Naphthalene,2-methyl	C11H10	1	1-Octadecanesulphonyl chloride	C18H37ClO2S	
Tridecane	C13H28	5	Naphthalene,2,6-dimethyl	C12H12	2	Behenyl chloride	C22H45Cl	
Heptacosane,1-chloro	C27H55Cl		Naphthalene,1,2,3,4-tetrahydro-2,7-dimethyl-	C12H16				
Hexadecane,2,6,10,14-tetramethyl	C20H42							
Cyclohexane,1,1,3-trimethyl-	C15H30							
Tetradecane	C14H30	4						
Hexadecane,7,9-dimethyl	C18H38							
Cyclohexane, octyl	C14H28							
Heptadecane	C17H36							
Pentadecane	C15H32	4						
Hexadecane	C16H34	2						

The table shows the hydrocarbon compounds with their molecular formular, and isomer present in the spent coolant waste.

Supplementary document 9

Table 2a: Hydrocarbon compounds detected after week one, two, and three of treatment of SCW by wild type microalgae

**Week 1**		**Week 2**		**Week 3**	
**Alkanes Compounds**	**Formula**	**Alkanes Compounds**	**Formula**	**Alkanes compounds**	**Formula**
Hexane,2,2,5-trimethyl-	C9H20	Cyclopentane,1,2,4-trimethyl-	C8H16	Heptane,2,2-dimethyl-	C9H20
Cyclopentane,1,2,4-trimethyl-	C8H16	Heptane,2,2-dimethyl-	C9H20	Hexadecane,2,6,11,15-tetramethyl	C20H42
Pentadecane,2,6,10-trimethyl-	C18H38	Cyclohexane,1,1,4,4-tetramethyl-	C10H20	Cyclohexane,1,2-dimethyl-, trans-	C8H16
Eicosane,10-methyl-	C21H44	Nonane,4-methyl-	C10H22	Heptadecane,2,6,10,15, tetramethyl	C21H44
Tetradecane,2,6,10-trimethyl-	C17H36	Dodecane,2,7,10-trimethyl-	C15H32	2-Cyclohexylnonadecane	C25H50
Tetradecane,4,11-dimethyl-	C16H34	Pentadecane, 3-methyl-	C16H34	Octane,3,3-dimethyl-	C10H22
Heptadecane,2,6,10,15-tetramethyl-	C21H44	2-methylhexacosane	C27H56	Dodecane,2,7,10-trimethyl-	C15H32
Heneicosane,11-(1-ethylpropyl)-	C26H54	Undecane,4,8-dimethyl-	C13H28	Tetradecane,2,6,10-trimethyl-	C17H36
**Alcohols**		10-Methylnonadecane	C20H42	Cyclooctane,1,2-dimethyl-	C10H20
n-Tridecan-1-ol	C13H28O	**Alcohols**		**Alcohols**	
1-Heptacosanol	C27H56O	1-Decanol,2-octyl-	C18H38O	1-Decanol,2-methyl-	C11H24O
n-Tetracosanol-1	C24H50O	n-Heptadecanol-1	C17H36O	1-Dodecanol, 2-hexyl-	C18H38O
**Monoaromatic compounds**		11-Methyldodecanol	C13H28O	7-Heptadecanol,7-methyl-	C18H38O
Benzene,1,3-dimethyl-	C8H10	cis-1,2-Cyclododecanediol	C12H24O2	Cholesta-8,24-dien-3-ol,4methyl	C26H46O
Benzene,1-ethyl-3-methyl-	C9H12	**Monoaromatic compounds**		n-Nonadecanol-1	C19H40O
Benzene,2-ethyl-1,4-dimethyl	C10H14	Benzene,1,3-dimethyl-	C8H10	n-Tridecan-1-ol	C13H28O
1,2-Benzenedicarboxylic acid	C8H6O4	3-Trifluoromethylbenzoic acid, dodecyl ester	C20H28F4O	9-Undecenol,2,10-dimethyl-	C13H26O
Benzhydrol ether	C26H22O	Benzeneacetic acid, 4-tridecyl ester	C21H34O2	1-Nonanol,4,8-dimethyl-	C11H24O
**PAHs**		Benzene,1,4-diethyl-	C10H14	1-Decanol,2-octyl-	C18H38O
Naphthalene,1-methyl	C11H10	1,4-Benzenediol,2,6-bis(1,1-dimethylethyl)-	C14H22O2	Z, E-3,13-Octadecadien-1-ol	C18H34O
Naphthalene,1,3-dimethyl-	C12H12	1,2-Benzenedicarboxylic acid, butyl 2-methylpropyl	C16H22O4	**Fatty acids/other compounds**	
1H-Indene,2,3-dihydro-4-methyl-	C10H12	**PAHs**		Malonic acid, neopentyl tridecyl ester	C21H40O4
1,2,3,4-tetrahydro-1,2-naphthalenediol	C10H12O2	1H-Indene,2,3-dihydro-4-methyl-	C10H12	Dodecanoic acid, methyl ester	C13H26O2
1,2-Naphthalenediol	C10H8O2	Decahydro-4,4,8,9,10-pentamethyl naphthalene	C15H28	Fumaric acid, ethyl heptadecyl ester	C23H42O4
1-Naphthol,1,2,3,4-tetrahydro-2-methyl	C11H14O	1-Naphthol,1,2,3,4-tetrahydro-2-methyl-	C11H14O	Hydrazine carboxylic acid	C8H10N2O2
**Fatty acid/other compounds**		**Fatty acids/other compounds**		Trichloroacetic acid,1-cyclopentyl ethyl ester	C9H13Cl3O
Dichloroacetic acid, 2-tridecyl ester	C15H28Cl	Decadienedioic acid diethyl ester	C14H26O4	Palmitoleic acid	C16H30O2
Sulfurous acid, dicyclohexyl ester	C12H22O3S	Palmitoleic acid	C16H30O2	Acetic acid, trifluoro-, dodecyl ester	C14H25F3O2
Palmitoleic acid	C16H30O2	Carbonic acid, ethyl isobutyl ester	C7H14O3	Tetradecanoic acid, 2,3-dihydroxypropyl	C17H34O4
Malonic acid,2-heptyltetradecyl ester	C24H46O4	Octadecanoic acid	C18H36O2	Tridecanoic acid	C13H26O2
Fumaric acid, ethyl heptadecyl ester	C23H42O4	Trichloroacetic acid,1-cyclopentyl ethyl ester	C9H13Cl3O		
Acetic acid, trifluoro-, dodecyl ester	C14H25F3O2	Octanoic acid, tetradecyl ester	C22H44O2		
Tetradecanoic acid, 2,3-dihydroxypropyl	C17H34O4				

The table shows the list of extracellular hydrocarbons present with their molecular formula after wild-type microalgae treatment of SCW.

Table 2b: Hydrocarbon compounds detected after week four and five of treatment of SCW by wild type microalgae

**Week 4**		**Week 5**	
**Alkanes Compounds**	**Formula**	**Alcohols**	**Formula**
Hexane,3,3,4-trimethyl-	C9H20	1-Decanol,2-methyl-	C11H24O
Decane,3,6-dimethyl-	C12H26	Tridecanol,2-ethyl-2-methyl	C16H34O
3-Hexane,3-ethyl-2,5-dimethyl	C10H22	2-Isopropyl-5-methyl-1-heptanol	C11H24O
Heptadecane,2,6,10,15-tetramethyl-	C21H44	11-Methyldodecanol	C13H28O
Heneicosane,5-methyl	C22H46	1-Dodecanol, 2-octyl-	C18H38O
Tetradecane,2,6,10-trimethyl-	C17H36	2-Tridecen-1-ol, (E)-	C13H26O
Undecane,4,8-dimethyl-	C13H28	**Fatty acids/other compounds**	
Dodecane,2,6,11-trimethyl-	C15H32	Pentadecanoic acid	C15H30O2
Heptadecane,2-methyl-	C18H38	Octanoic acid, hexadecyl ester	C24H48O2
**Alcohols**		Methyl tetradecanoate	C15H30O2
1-Octanol,2-butyl-	C12H26O	4-Bromobutanoic acid, heptadecyl ester	C21H41BrO2
1-Nonanol,4,8-dimethyl-	C11H24O	Trichloroacetic acid,1-cyclopentyl ethyl ester	C9H13Cl3O2
n-Tridecan-1-ol	C13H28O	Tridecanoic acid, methyl ester	C14H28O2
11-Methyldodecanol	C13H28O	Fumaric acid, 8-chlorooctyl hexyl ester	C18H31ClO4
Cyclododecanemethanol	C13H26O	Methoxyacetic acid, 4-hexadecyl ester	C19H38O3
1-Decanol, 2-octyl-	C18H38O	Dichloroacetic acid, tridecyl ester	C15H28Cl2O2
n-Tetracosanol-1	C24H50O	Palmitoleic acid	C16H30O2
**Fatty acids/other compounds**			
Decanoic acid	C10H20O2		
Palmitoleic acid	C16H30O2		
Carbonic acid, ethyl isobutyl ester	C7H14O3		
Hexadecanoic acid	C16H32O2		
Nonanoic acid	C9H18O2		
Dodecanoic acid, methyl ester	C13H26O2		
Dichloroacetic acid, tridecyl ester	C15H28Cl2O2		
Formic acid,2-ethylhexyl ester	C9H18O2		
4-Bromobutanoic acid, heptadecyl ester	C21H41BrO2		

The table shows the list of extracellular hydrocarbons present with their molecular formula after wild-type microalgae treatment of SCW.

Supplementary document 10

Table 3a: Hydrocarbon compounds detected after week one, two and three of treatment of spent coolant waste by UV exposed *S. vacuolatus*

**Week 1**		**Week 2**		**Week 3**	
**Alkanes Compounds**	**Formula**	**Alkanes Compounds**	**Formula**	**Alkanes compounds**	**Formula**
Pentadecane,2,6,10,14-tetramethyl	C16H34	Cyclotridecane	C13H26	Undecane,4,7-dimethyl-	C13H28
Decane,4-ethyl	C12H26	Hexadecane,4-methyl-	C17H36	Dodecane,2,6,11-trimethyl-	C15H32
Nonane,2,3-dimethyl	C11H24	Tetradecane,4,11-dimethyl-	C16H34	Pentadecane,3-methyl-	C16H34
Dodecane,2,6,11-trimethyl	C15H32	Hexadecane,2,6,11,15-tetramethyl-	C20H42	Heptadecane,2,6,10,15, tetramethyl	C21H44
Hexadecane,2,6,11,15-tetramethyl	C20H42	2,2-Dimethyldodecane	C14H30	Tetrapentacontane,1,54-dibromo-	C54H108Br2
Heptadecane,2,6,10,15-tetramethyl	C21H44	Heptadecane,2,6-dimethyl-	C19H40	Hexadecane,7,9-dimethyl	C18H38
**Alcohols**		Undecane,2,4-dimethyl	C13H28	**Alcohols**	
1-Decanol, 2-octyl	C18H36O	**Alcohols**		n-Tridecan-1-ol	C13H28O
9,12-Octadecadien-1-ol	C18H34O	n-Tridecan-1-ol	C13H28O	Cyclohexanol,2,4-dimethyl-	C8H16O
n-Tetracosanol-1	C24H50O	Cyclohexanol,2,4-dimethyl-	C8H16O	1-Hexadecanol,3,7,11,15-tetramethyl	C20H40O
**Monoaromatic compounds**		1-Hexadecanol,3,7,11,15-tetramethyl	C20H40O	**Ketones**	
1,2-Benzenediol, 4-[2-[[3-(4-hydroxyphenyl)-1-	C18H23NO3	1-Decanol,2-hexyl	C16H34O	5,8-Tridecadione	C19H34O2
methylDihydroartemisininoxymethyl benzoic acid	C23H30O7	6,10,13-Trimethyl tetradecanol	C5H11ClO	4,4-Dimethyl-2-allylcyclohexanone	C11H18O
Benzyl alcohol,4-methoxy-6-fluoro	C8H9FO2	n-Nonadecanol-1	C19H40O	3,6-Undecandione	C17H36O
Benzyloxy tridecanoic acid	C20H32O3	Tetracontane-1,40-diol	C40H82O2	2-Pentadecanone, 6,10,14-trimethyl-	C18H36O
Phthalic acid	C8H6O4	1,4-cyclohexanediol	C6H12O2	1,3-Cyclohexanedione,5,5-dimethyl-2,2-dipropyl-	C13H24O2
1,2-Benzenedicarboxylic acid, butyl,2-methyl propyl ester	C16H22O4	n-Nonadecanol-1	C19H40O		
Phthalic acid, di(2-propylpentyl) ester	C24H38O	n-Tetracosanol-1	C24H50O		
2,5-di-tert-Butyl-1,4-benzoquinone	C14H20O2	1-Dodecanol	C12H26O	**Fatty acids/other compounds**	
**PAHs**		**Fatty acids/other compounds**		Pentadecanoic acid,3-methyl-, methyl ester	C17H34O2
				Octadecanoic acid,9,10-epoxy-, isopropyl	C19H36O3
2-Ethyl-1-methyl-1,2,3,4-tetrahydro-1,2-naphthalenediol	13H18O2	Cyclohexane carboxylic acid,4-pentyl-,2,3-dicyano-4-ethoxyphenyl ester	C20H24N2O3		
				Cyclopentane carboxylic acid	C6H10O2
1-Naphthol,1,2,3,4-tetrahydro-2-methyl	C11H14O	Acetic acid,7,7-dimethyl-2-oxobicyclohept-1-ylmethyl ester	C12H18O3	Octadecanoate	
1,5-Naphthalenediol, decahydro	C10H18O2				C18H35O2
3,4-dihydro-3-methylnaphthalen-1-one	C11H12O	Dodecanoic acid, 2,2,2-trifluoroethyl ester	C14H25F3O2	Hexadecanoate	
2-Naphthoic acid, 3-methoxy-4-methyl	C25H20O6	Nonahexacontanoic acid	C69H138O2		C16H31O2
1-Naphthaleneacetic acid	C12H10O2	cis-13-Eicosenoic acid	C18H36O	6-Octadecenoic, acid, methyl ester, (Z)-	
2-Naphthalenecarboxylic acid, 4,4'-methylenebis	C23H14Li2O6	Alpha-hydroxyisocaproic acid	C6H12O3		C19H36O2
**Fatty acids/other compounds**		Octadecanoic acid	C18H36O2	Palmitoleic acid	C16H30O2
		Linoleic acid	C18H32O2		
Tetradecanoic acid	C14H28O2	Palmitoleic acid	C16H30O2	8-Octadecenoic acid	C18H34O2
Pentadecanoic acid	C15H30O2	Eicosapentaenoic acid	C20H30O2	hexanedioic acid beta-citronellyl octyl ester	C26H46O4
Cyclopentane carboxylic acid,3-tridecyl ester	C20H38O2	Dodecanoic acid, cyclohexyl ester	C18H34O2	Undecanoic acid	C11H22O2
Formic acid,2-ethylhexyl ester	C9H18O2	Dichloroacetic acid, heptadecyl ester	C19H36Cl2O2		
Cyclohexane carboxylic acid,4-pentyl-,2,3-dicyano-4	C22H28N2O3	Methoxyacetic acid, 4-hexadecyl ester	C19H38O3		
6-Octadecenoic acid, methyl ester, (Z)-	C19H36O2	3-Methylcyclohexane carboxylic acid	C8H14O2		
Hexadecanoic acid, (3-bromoprop-2-ynyl) ester	C19H33BrO2	Hexanedioic acid, bis(2-ethylhexyl) ester	C22H42O		
Cyclohexane, carboxylic acid	C7H12O2	Nonanoic acid, nonyl ester	C18H36O2		

The table shows the list of extracellular hydrocarbons present with their molecular formula after mutagenized microalgal treatment of SCW.

Table 3b: Hydrocarbon compounds detected after week four and five treatment of SCW by UV exposed *S. vacuolatus*

**Week 4**		**Week 5**
**Fatty acids/other compounds**	**Formula**	
Hexanoic acid	$C_6H_{12}O_2$	**Hydrocarbons not detected**
Heptanoic acid	$C_7H_{14}O_2$	
Octanoic acid	$C_8H_{16}O_2$	
Tridecanoic acid	$C_{13}H_{26}O_2$	
Pentadecanoic acid	$C_{15}H_{30}O_2$	
Acetic acid, trifluoro-, dodecyl ester	$C_{14}H_{25}F_3O_2$	
Tetradecanoic acid, 2,3-dihydroxypropyl ester	$C_{17}H_{34}O_4$	
Hexadecanoic acid,2,3-bis(acetyloxy)propyl ester	$C_{23}H_{42}O_6$	
n-Decanoic acid	$C_{10}H_{20}O_2$	
Hexanedioic acid, dioctyl ester	$C_{22}H_{42}O_4$	
alpha-Ketostearic acid	$C_{18}H_{34}O_3$	
Octadecanoic acid,2,3-bis[(1-oxotetradecyl) oxy] propyl ester	$C_{49}H_{94}O_6$	
9-Oxononanoic acid	$C_9H_{16}O_3$	
Dodecanoic acid	$C_{12}H_{24}O_2$	
Octanedioic acid	$C_8H_{14}O_4$	
Palmitoleic acid	$C_{16}H_{30}O_2$	
Tridecanoic acid, methyl ester	$C_{14}H_{28}O_2$	
Octadecanoic acid,9,10-epoxy-, isopropyl ester	$C_{19}H_{36}O_3$	
Oleic acid	$C_{18}H_{34}O_2$	
Cyclohexane carboxylic acid hydrazide	$C_7H_{14}N_2O$	
Palmitic acid	$C_{16}H_{32}O_2$	
Decanoic acid,5,5-dimethyl-9-oxo-, methyl ester	$C_{13}H_{24}O_3$	

The table shows the list of extracellular hydrocarbons present with their molecular formula after mutagenized *S. vacuolatus* treatment of SCW.

Supplementary document 11

GCMS chromatogram of hydrocarbons of SCW treated by the wild-type microalgae

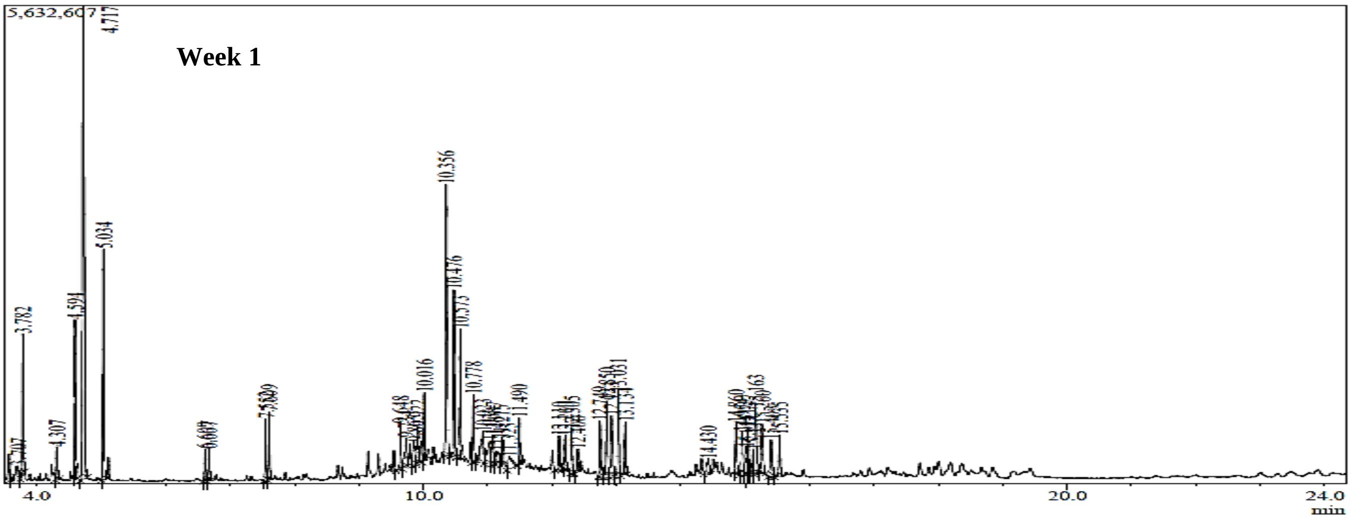

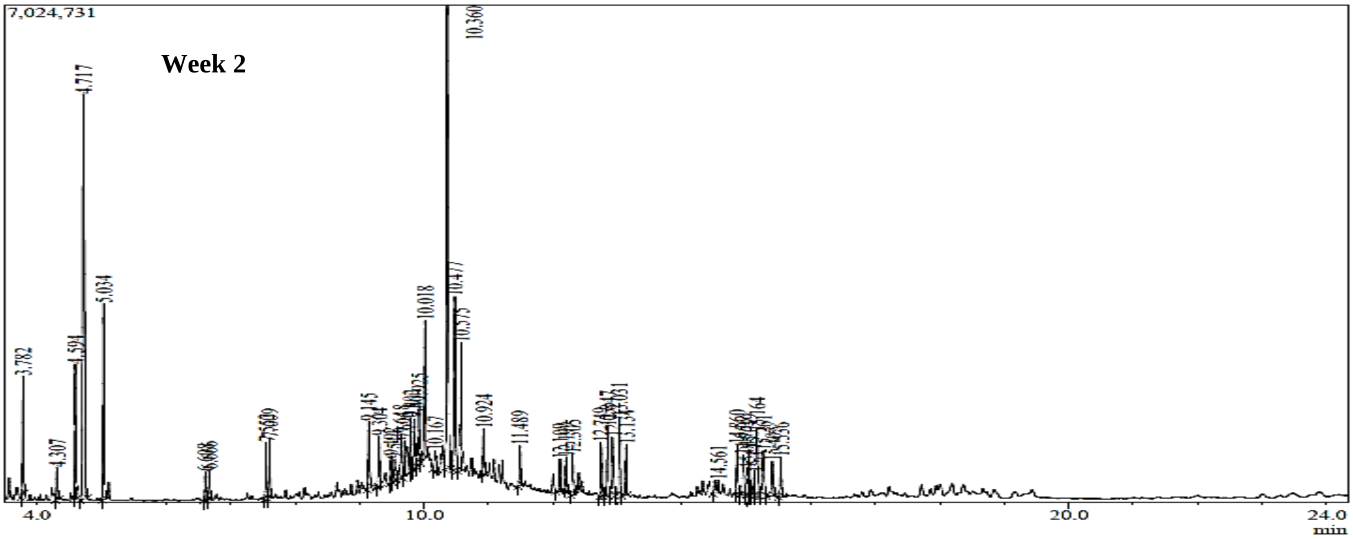

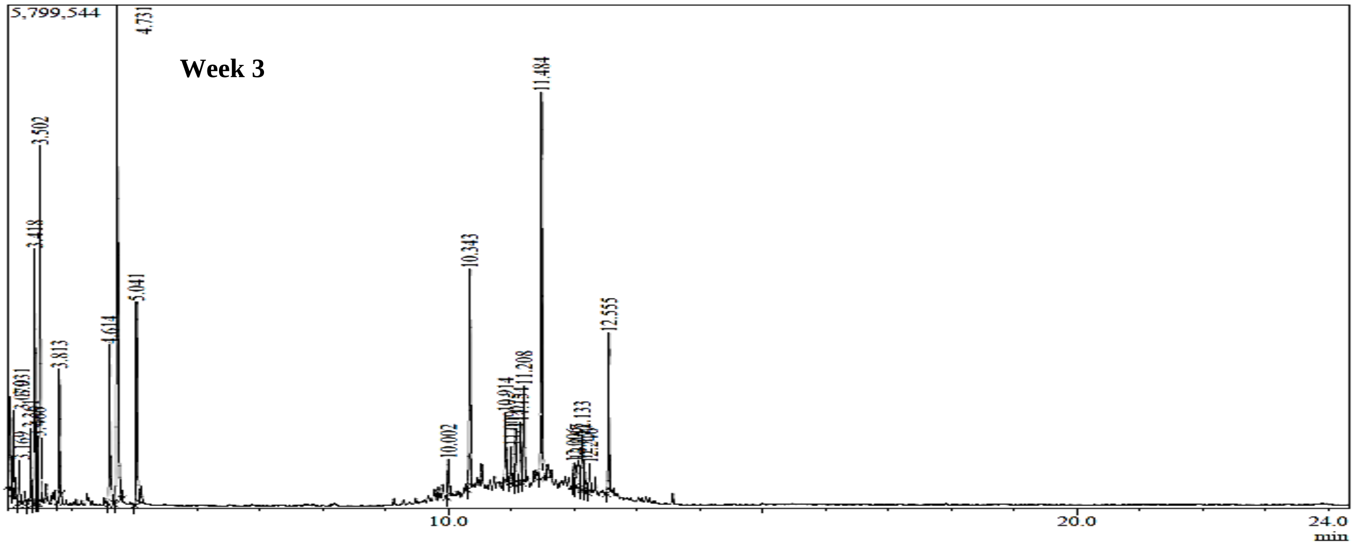

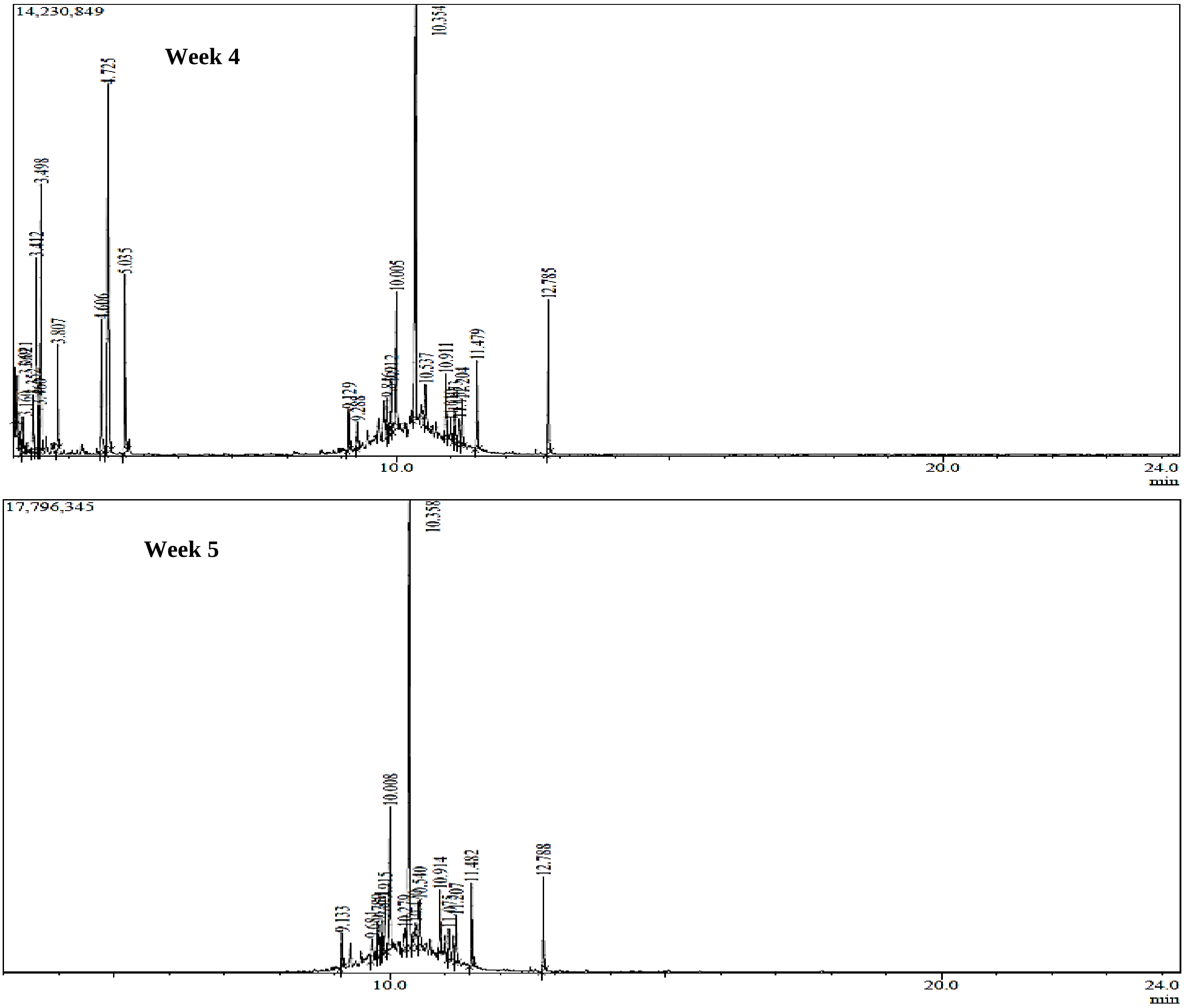

Fig. S11: The gas chromatogram of spent coolant waste at different weeks of degradation by the wild-type. The numbers on the chromatogram peaks indicates the elution time of the hydrocarbon compounds.

Supplementary document 12

GCMS chromatogram of hydrocarbons of SCW treated by the UV exposed *S. vacuolatus*

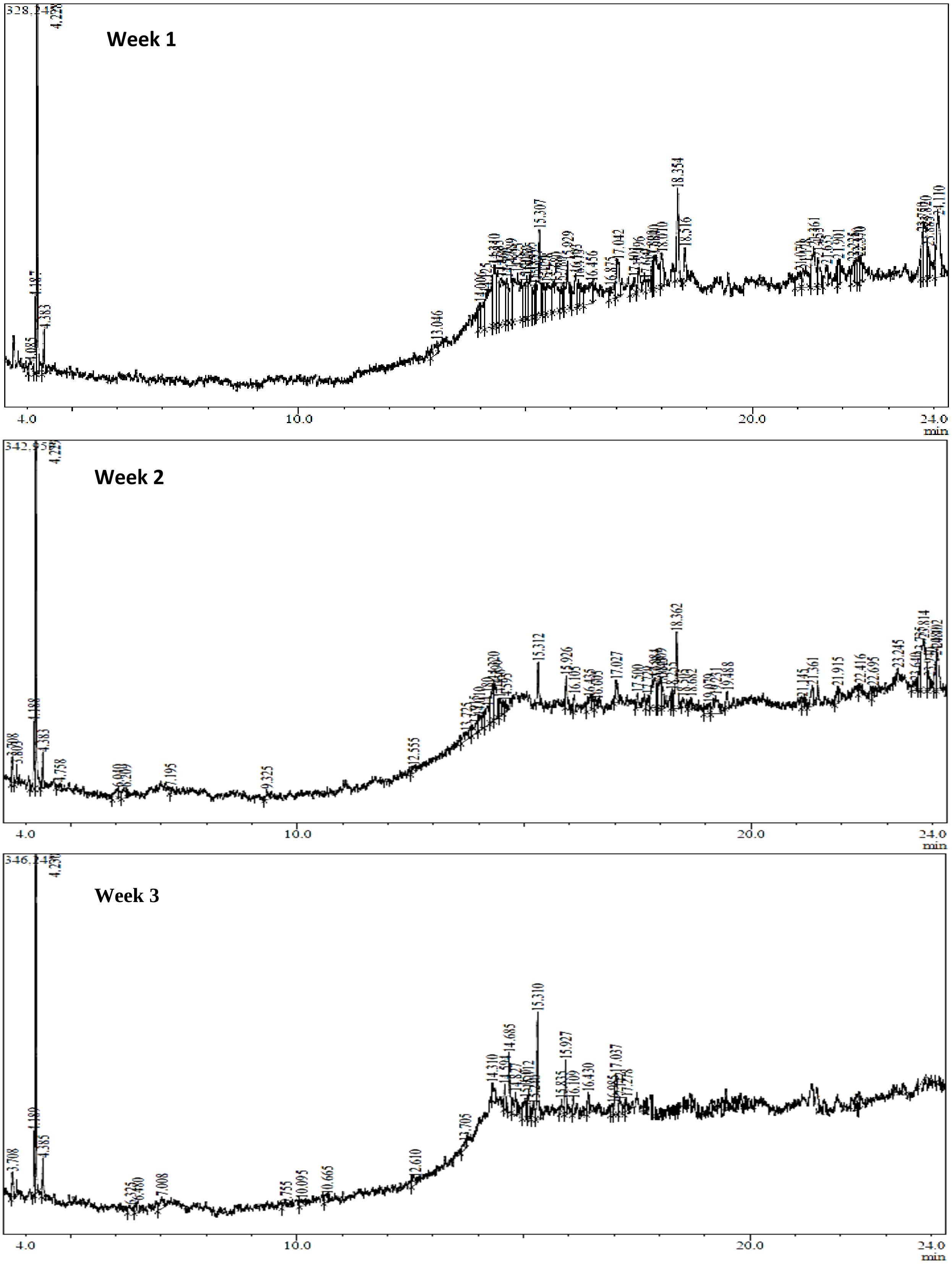

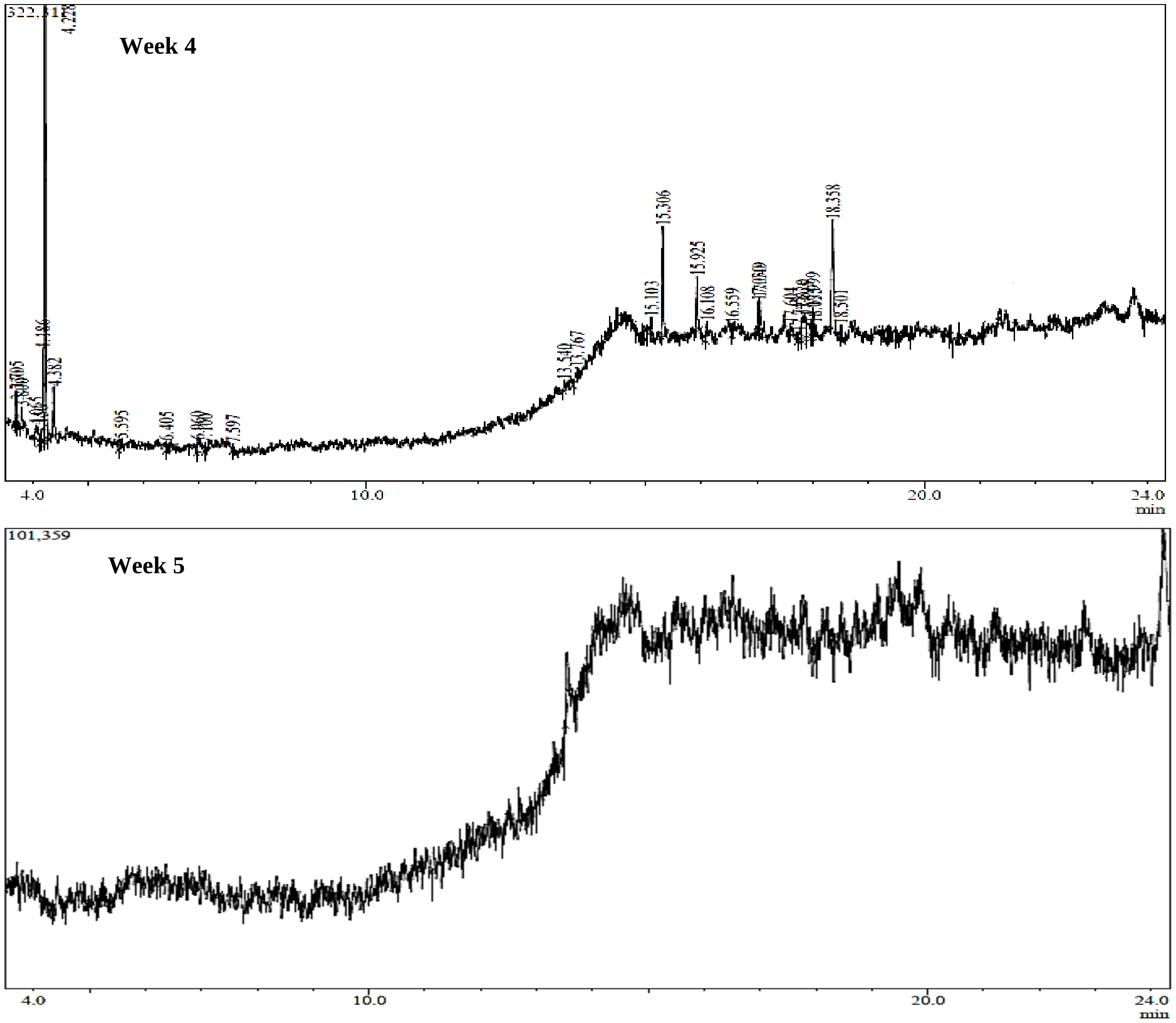

Fig. S12: The gas chromatogram of spent coolant waste at different weeks of degradation by the UV exposed *S. vacuolatus*. The numbers on the chromatogram peaks indicates the elution time of the hydrocarbon compounds.

Supplementary document 13

Statistical analysis of the biomass concentration at different UV time exposure

ANOVA

		Sum of Squares	df	Mean Square	F	Sig.
WT	Between Groups	.000	6	.000	12.670	<,001
	Within Groups	.000	14	.000		
	Total	.000	20			
UV_2hrs	Between Groups	.004	6	.001	31.164	<,001
	Within Groups	.000	14	.000		
	Total	.004	20			
UV_4hrs	Between Groups	.008	6	.001	12.675	<,001
	Within Groups	.002	14	.000		
	Total	.010	20			
UV_6hrs	Between Groups	.014	6	.002	9.492	<,001
	Within Groups	.003	14	.000		
	Total	.017	20			
UV_12hrs	Between Groups	.040	6	.007	37.084	<,001
	Within Groups	.003	14	.000		
	Total	.043	20			
UV_24hrs	Between Groups	.062	6	.010	32.356	<,001
	Within Groups	.004	14	.000		
	Total	.067	20			
UV_48hrs	Between Groups	.103	6	.017	154.882	<,001
	Within Groups	.002	14	.000		
	Total	.105	20			

Supplementary document 14

Statistical analysis of the Chlorophyll a

ANOVA

		Sum of Squares	df	Mean Square	F	Sig.
ChLa_D0	Between Groups	4.376	1	4.376	8.734	.042
	Within Groups	2.004	4	.501		
	Total	6.380	5			
Chla_D3	Between Groups	82.140	1	82.140	2386.542	<,001
	Within Groups	.138	4	.034		
	Total	82.278	5			
Chla_D6	Between Groups	6.876	1	6.876	55.001	.002
	Within Groups	.500	4	.125		
	Total	7.376	5			
Chla_D9	Between Groups	21.083	1	21.083	3484.423	<,001
	Within Groups	.024	4	.006		
	Total	21.107	5			
Chla_D12	Between Groups	14.931	1	14.931	2467.736	<,001
	Within Groups	.024	4	.006		
	Total	14.955	5			
Chla_D15	Between Groups	27.234	1	27.234	20655.428	<,001
	Within Groups	.005	4	.001		
	Total	27.239	5			
Chla_D18	Between Groups	1.763	1	1.763	349.027	<,001
	Within Groups	.020	4	.005		
	Total	1.783	5			
Chla_D21	Between Groups	6.242	1	6.242	10.206	<,001
	Within Groups	2.447	4	.612		
	Total	8.689	5			

Supplementary document 15

Statistical analysis of the Chlorophyll b

ANOVA

		Sum of Squares	df	Mean Square	F	Sig.
ChLb_D0	Between Groups	6.786	1	6.786	251.779	<,001
	Within Groups	.108	4	.027		
	Total	6.894	5			
Chlb_D3	Between Groups	90.863	1	90.863	3449.279	<,001
	Within Groups	.105	4	.026		
	Total	90.968	5			
Chlb_D6	Between Groups	2.965	1	2.965	143.592	<,001
	Within Groups	.083	4	.021		
	Total	3.048	5			
Chlb_D9	Between Groups	8.662	1	8.662	974.858	<,001
	Within Groups	.036	4	.009		
	Total	8.697	5			
Chlb_D12	Between Groups	4.887	1	4.887	184.943	<,001
	Within Groups	.106	4	.026		
	Total	4.993	5			
Chlb_D15	Between Groups	20.646	1	20.646	3215.661	<,001
	Within Groups	.026	4	.006		
	Total	20.672	5			
Chlb_D18	Between Groups	.180	1	.180	50.835	.002
	Within Groups	.014	4	.004		
	Total	.194	5			
Chlb_D21	Between Groups	10.741	1	10.741	1290.654	<,001
	Within Groups	.033	4	.008		
	Total	10.775	5			

Supplementary document 16

Statistical analysis of the Carotenoid

ANOVA

		Sum of Squares	df	Mean Square	F	Sig.
Carot_D0	Between Groups	6.786	1	6.786	251.779	<,001
	Within Groups	.108	4	.027		
	Total	6.894	5			
Carot_D3	Between Groups	90.863	1	90.863	3449.279	<,001
	Within Groups	.105	4	.026		
	Total	90.968	5			
Carot_D6	Between Groups	2.965	1	2.965	143.592	<,001
	Within Groups	.083	4	.021		
	Total	3.048	5			
Carot_D9	Between Groups	8.662	1	8.662	974.858	<,001
	Within Groups	.036	4	.009		
	Total	8.697	5			
Carot_D12	Between Groups	4.887	1	4.887	184.943	<,001
	Within Groups	.106	4	.026		
	Total	4.993	5			
Carot_D15	Between Groups	20.646	1	20.646	3215.661	<,001
	Within Groups	.026	4	.006		
	Total	20.672	5			
Carot_D18	Between Groups	.180	1	.180	50.835	.002
	Within Groups	.014	4	.004		
	Total	.194	5			
Carot_D21	Between Groups	10.741	1	10.741	1290.654	<,001
	Within Groups	.033	4	.008		
	Total	10.775	5			

Supplementary document 17

Statistical analysis of the protein concentration

ANOVA

		Sum of Squares	df	Mean Square	F	Sig.
PC_D0	Between Groups	1.585	1	1.585	2.439	.193
	Within Groups	2.599	4	.650		
	Total	4.185	5			
PC_D3	Between Groups	29.384	1	29.384	2661.373	<,001
	Within Groups	.044	4	.011		
	Total	29.428	5			
PC_D6	Between Groups	8.067	1	8.067	15.632	.017
	Within Groups	2.064	4	.516		
	Total	10.131	5			
PC_D9	Between Groups	3.042	1	3.042	5.252	.084
	Within Groups	2.317	4	.579		
	Total	5.358	5			
PC_D12	Between Groups	3.878	1	3.878	7.198	.055
	Within Groups	2.155	4	.539		
	Total	6.034	5			
PC_D15	Between Groups	.913	1	.913	.851	.409
	Within Groups	4.292	4	1.073		
	Total	5.204	5			
PC_D18	Between Groups	.156	1	.156	.271	.630
	Within Groups	2.313	4	.578		
	Total	2.469	5			
PC_D21	Between Groups	76.255	1	76.255	166.405	<,001
	Within Groups	1.833	4	.458		
	**Total**	**78.088**	**5**			

Supplementary document 18

Statistical analysis of the carbohydrate accumulation

ANOVA

		Sum of Squares	df	Mean Square	F	Sig.
CHO_D0	Between Groups	.000	1	.000	.	.
	Within Groups	.000	4	.000		
	Total	.000	5			
CHO_D3	Between Groups	37.951	1	37.951	1897567.500	<,001
	Within Groups	.000	4	.000		
	Total	37.951	5			
CHO_D6	Between Groups	44.652	1	44.652	10625.081	<,001
	Within Groups	.017	4	.004		
	Total	44.669	5			
CHO_D9	Between Groups	134.663	1	134.663	71897.190	<,001
	Within Groups	.007	4	.002		
	Total	134.671	5			
CHO_D12	Between Groups	100.099	1	100.099	2780523.375	<,001
	Within Groups	.000	4	.000		
	Total	100.099	5			
CHO_D15	Between Groups	220.305	1	220.305	1608067.456	<,001
	Within Groups	.001	4	.000		
	Total	220.306	5			
CHO_D18	Between Groups	564.249	1	564.249	1929056.538	<,001
	Within Groups	.001	4	.000		
	Total	564.250	5			
CHO_D21	Between Groups	334.357	1	334.357	11529563.793	<,001
	Within Groups	.000	4	.000		
	Total	334.357	5			

Supplementary document 19

Statistical analysis of the Lipid accumulation

ANOVA

		Sum of Squares	df	Mean Square	F	Sig.
Lip_D0	Between Groups	.000	1	.000	14.266	.019
	Within Groups	.000	4	.000		
	Total	.000	5			
Lip_D3	Between Groups	.000	1	.000	9.936	.034
	Within Groups	.000	4	.000		
	Total	.000	5			
Lip_D6	Between Groups	.000	1	.000	10.521	.032
	Within Groups	.000	4	.000		
	Total	.000	5			
Lip_D9	Between Groups	.000	1	.000	63.075	.001
	Within Groups	.000	4	.000		
	Total	.000	5			
Lip_D12	Between Groups	.000	1	.000	11.286	.028
	Within Groups	.000	4	.000		
	Total	.000	5			
Lip_D15	Between Groups	.000	1	.000	19.898	.011
	Within Groups	.000	4	.000		
	Total	.000	5			
Lip_D18	Between Groups	.000	1	.000	71.813	.001
	Within Groups	.000	4	.000		
	Total	.000	5			
Lip_D21	Between Groups	.000	1	.000	261.664	<,001
	Within Groups	.000	4	.000		
	Total	.000	5			

Supplementary document 20

Statistical analysis of the substrate versatility
Na2CO3

ANOVA

		Sum of Squares	df	Mean Square	F	Sig.
Na2CO3_D0	Between Groups	.778	1	.778	2.596	.182
	Within Groups	1.198	4	.300		
	Total	1.976	5			
Na2CO3_D3	Between Groups	.019	1	.019	361.387	<,001
	Within Groups	.000	4	.000		
	Total	.019	5			
Na2CO3_D6	Between Groups	.017	1	.017	8.211	.046
	Within Groups	.008	4	.002		
	Total	.025	5			
Na2CO3_D9	Between Groups	.023	1	.023	2.445	.193
	Within Groups	.038	4	.010		
	Total	.062	5			
Na2CO3_D12	Between Groups	.095	1	.095	49.925	.002
	Within Groups	.008	4	.002		
	Total	.103	5			
Na2CO3_D15	Between Groups	.117	1	.117	898.165	<,001
	Within Groups	.001	4	.000		
	Total	.117	5			
Na2CO3_D18	Between Groups	.090	1	.090	12.484	.024
	Within Groups	.029	4	.007		
	Total	.119	5			
Na2CO3_D21	Between Groups	.183	1	.183	82.002	<,001
	Within Groups	.009	4	.002		
	Total	.192	5			

Supplementary document 21

Statistical analysis of the substrate versatility
Glucose
ANOVA

		Sum of Squares	df	Mean Square	F	Sig.
Glucose_D0	Between Groups	.009	1	.009	3.512	.134
	Within Groups	.010	4	.003		
	Total	.020	5			
Glucose_D3	Between Groups	.010	1	.010	20.063	.011
	Within Groups	.002	4	.000		
	Total	.012	5			
Glucose_D6	Between Groups	.245	1	.245	20.875	.010
	Within Groups	.047	4	.012		
	Total	.292	5			
Glucose_D9	Between Groups	.002	1	.002	.234	.654
	Within Groups	.037	4	.009		
	Total	.039	5			
Glucose_D12	Between Groups	.003	1	.003	.684	.455
	Within Groups	.020	4	.005		
	Total	.024	5			
Glucose_D15	Between Groups	.033	1	.033	99.413	<,001
	Within Groups	.001	4	.000		
	Total	.034	5			
Glucose_D18	Between Groups	.050	1	.050	441.606	<,001
	Within Groups	.000	4	.000		
	Total	.051	5			
Glucose_D21	Between Groups	.077	1	.077	76.216	<,001
	Within Groups	.004	4	.001		
	Total	.081	5			

Supplementary document 22

Statistical analysis of the substrate versatility
Glycerol
ANOVA

		Sum of Squares	df	Mean Square	F	Sig.
Gly_D0	Between Groups	.002	1	.002	67.328	.001
	Within Groups	.000	4	.000		
	Total	.002	5			
Gly_D3	Between Groups	.002	1	.002	1464.100	<,001
	Within Groups	.000	4	.000		
	Total	.002	5			
Gly_D6	Between Groups	.009	1	.009	172.969	<,001
	Within Groups	.000	4	.000		
	Total	.009	5			
Gly_D9	Between Groups	.126	1	.126	10788.014	<,001
	Within Groups	.000	4	.000		
	Total	.126	5			
Gly_D12	Between Groups	.010	1	.010	22.197	.009
	Within Groups	.002	4	.000		
	Total	.011	5			
Gly_D15	Between Groups	.012	1	.012	1275.586	<,001
	Within Groups	.000	4	.000		
	Total	.012	5			
Gly_D18	Between Groups	.018	1	.018	377.173	<,001
	Within Groups	.000	4	.000		
	Total	.019	5			
Gly_D21	Between Groups	.007	1	.007	30.991	.005
	Within Groups	.001	4	.000		
	Total	.008	5			

Supplementary document 23

Statistical analysis of the dehydrogenase activity (TPF production) and TPH degradation during degradation

Parameters	Source of variation	df	Significant test of TPF production and TPH biodegradation				
			**Week 1**	**Week 2**	**Week 3**	**Week 4**	**Week 5**
**TPF production**	Replicates	6	0.0001382***	0.0005708***	0.001386**	0.00011962***	0.0001087***
	Treatment	1	0.2940601	2.4186258	1.396720	0.15414007	0.0582435*
	Error	6	0.0001969***	0.0003701***	0.001108**	0.00007190***	0.0001222***
**TPH biodegradation**	Replicates	6	0.0002279***	0.00007370***	0.00001615***	0.00011985***	0.00001428***
	Treatment	1	0.1307964	0.14384606	0.09809640	0.06709633	2.01476579
	Error	6	0.0002226***	0.00007928***	0.00002013***	0.00005173***	0.00001428***

Table 4: Accumulated analysis of variance for TPF production and TPH degradation

Key: df: degrees of freedom; TPF: triphenyl formazan; TPH: total petroleum hydrocarbon; asterisks *, **, and ***, indicates various levels of significance at $P < 0.05$; $P < 0.01$; $P < 0.001$, respectively. Mean of six replicates (n=6). This was extrapolated from the statistical analysis and summarised.

Supplementary document 24

Statistical analysis for dehydrogenase activity analysis of wild-type and UV exposed *S. vacuolatus*

Analysis of variance

Variate: week1

Source of variation	d.f.	s.s.	m.s.	v.r.	F pr.
Replicates stratum	6	0.0008294	0.0001382	0.70	
Replicates.*Units* stratum					
Treatment	1	0.2940601	0.2940601	1493.41	<.001
Residual	6	0.0011814	0.0001969		
Total	13	0.2960709			

Tables of means

Variate: week1

Grand mean 0.4901

Treatment	A	B
	0.3451	0.6350

Standard errors of differences of means

Table	Treatment
rep.	7
d.f.	6
s.e.d.	0.00750

Analysis of variance

Variate: week2

Source of variation	d.f.	s.s.	m.s.	v.r.	F pr.
Replicates stratum	6	0.0034247	0.0005708	1.54	
Replicates.*Units* stratum					
Treatment	1	2.4186258	2.4186258	6534.72	<.001
Residual	6	0.0022207	0.0003701		
Total	13	2.4242712			

Tables of means

Variate: week2

Grand mean 0.9346

Treatment	A	B
	0.5190	1.3503

Standard errors of differences of means

Table	Treatment
rep.	7
d.f.	6
s.e.d.	0.01028

Analysis of variance

Variate: week3

Source of variation	d.f.	s.s.	m.s.	v.r.	F pr.
Replicates stratum	6	0.008314	0.001386	1.25	
Replicates.*Units* stratum					
Treatment	1	1.396720	1.396720	1260.25	<.001
Residual	6	0.006650	0.001108		
Total	13	1.411684			

Tables of means

Variate: week3

Grand mean 1.4549

Treatment	A	B
	1.1390	1.7707

Standard errors of differences of means

Table	Treatment
rep.	7
d.f.	6
s.e.d.	0.01779

Analysis of variance

Variate: week4

Source of variation	d.f.	s.s.	m.s.	v.r.	F pr.

Replicates stratum	6	0.00071771	0.00011962	1.66	
Replicates.*Units* stratum					
Treatment	1	0.15414007	0.15414007	2143.67	<.001
Residual	6	0.00043143	0.00007190		
Total	13	0.15528921			

Message: the following units have large residuals.

Replicates 2 *units* 1	0.0114	s.e.	0.0056
Replicates 2 *units* 2	-0.0114	s.e.	0.0056

Tables of means

Variate: week4

Grand mean 0.3276

Treatment	A	B
	0.2227	0.4326

Standard errors of differences of means

Table	Treatment
rep.	7
d.f.	6
s.e.d.	0.00453

Analysis of variance

Variate: week5

Source of variation	d.f.	s.s.	m.s.	v.r.	F pr.
Replicates stratum	6	0.0006524	0.0001087	0.89	
Replicates.*Units* stratum					
Treatment	1	0.0582435	0.0582435	476.75	<.001
Residual	6	0.0007330	0.0001222		
Total	13	0.0596289			

Message: the following units have large residuals.

Replicates 5	0.0146	s.e.	0.0068
Replicates 3 *units* 1	0.0155	s.e.	0.0072
Replicates 3 *units* 2	-0.0155	s.e.	0.0072

Tables of means

Variate: week5

Grand mean 0.1849

Treatment	A	B
	0.1204	0.2494

Standard errors of differences of means

Table	Treatment
rep.	7
d.f.	6
s.e.d.	0.00591

Supplementary document 25

Statistical analysis for GCMS total petroleum hydrocarbon (TPH) of wildtype and UV exposed *S. vacuolatus*

Analysis of variance

Variate: week1

Source of variation	d.f.	s.s.	m.s.	v.r.	F pr.
Replicates stratum	6	0.0013673	0.0002279	1.02	
Replicates.*Units* stratum					
Treatment	1	0.1307964	0.1307964	587.52	<.001
Residual	6	0.0013358	0.0002226		
Total	13	0.1334995			

Tables of means

Variate: week1

Grand mean 0.5555

Treatment	A	B
	0.4589	0.6522

Standard errors of differences of means

Table	Treatment
rep.	7
d.f.	6
s.e.d.	0.00798

Least significant differences of means (5% level)

Table	Treatment
rep.	7
d.f.	6
l.s.d.	0.01952

Analysis of variance

Variate: week2

Source of variation	d.f.	s.s.	m.s.	v.r.	F pr.
Replicates stratum	6	0.00044221	0.00007370	0.93	

Replicates.*Units* stratum					
Treatment	1	0.14384606	0.14384606	1814.34	<.001
Residual	6	0.00047570	0.00007928		
Total	13	0.14476396			

Tables of means

Variate: week2

Grand mean 0.7244

Treatment	A	B
	0.6230	0.8257

Standard errors of differences of means

Table	Treatment
rep.	7
d.f.	6
s.e.d.	0.00476

Least significant differences of means (5% level)

Table	Treatment
rep.	7
d.f.	6
l.s.d.	0.01165

Analysis of variance

Variate: week3

Source of variation	d.f.	s.s.	m.s.	v.r.	F pr.
Replicates stratum	6	0.00009689	0.00001615	0.80	
Replicates.*Units* stratum					
Treatment	1	0.09809640	0.09809640	4874.18	<.001
Residual	6	0.00012075	0.00002013		
Total	13	0.09831404			

Tables of means

Variate: week3

Grand mean 0.7996

Treatment	A	B
	0.7159	0.8833

Standard errors of differences of means

Table	Treatment
rep.	7
d.f.	6
s.e.d.	0.00240

Least significant differences of means (5% level)

Table	Treatment
rep.	7
d.f.	6
l.s.d.	0.00587

Analysis of variance

Variate: week4

Source of variation	d.f.	s.s.	m.s.	v.r.	F pr.
Replicates stratum	6	0.00071907	0.00011985	2.32	
Replicates.*Units* stratum					
Treatment	1	0.06709633	0.06709633	1297.01	<.001
Residual	6	0.00031039	0.00005173		
Total	13	0.06812579			

Tables of means

Variate: week4

Grand mean 0.8533

Treatment	A	B
	0.7841	0.9226

Standard errors of differences of means

Table	Treatment
rep.	7
d.f.	6
s.e.d.	0.00384

Least significant differences of means (5% level)

Table	Treatment
rep.	7
d.f.	6
l.s.d.	0.00941

Analysis of variance

Variate: week5

Source of variation	d.f.	s.s.	m.s.	v.r.	F pr.
Replicates stratum	6	0.00008565	0.00001428	1.00	
Replicates.*Units* stratum					
Treatment	1	2.01476579	2.01476579	1.411E+05	<.001
Residual	6	0.00008565	0.00001428		
Total	13	2.01493709			

Tables of means

Variate: week5

Grand mean 0.4794

Treatment	A	B
	0.8587	0.1000

Standard errors of differences of means

Table	Treatment
rep.	7
d.f.	6
s.e.d.	0.00202

Least significant differences of means (5% level)

Table	Treatment
rep.	7
d.f.	6
l.s.d.	0.00494
