# Supplementary material for: Effect of ultra-violet light radiation on Scenedesmus vacuolatus growth kinetics, metabolic performance, and preliminary biodegradation study
Source: Biodegradation. 2023 Apr 13;35(1):71–86. doi: 10.1007/s10532-023-10029-2 (PMC10774200; doi:10.1007/s10532-023-10029-2)
Supplement: Supplementary file 2 — Supplementary file2 (DOCX 51498 KB) [file 10532_2023_10029_MOESM2_ESM.docx]

**Supplementary documents**

**Effect of ultra-violet light radiation on *Scenedesmus vacuolatus* growth: Process kinetics, metabolic performance and preliminary biodegradation study**

**Stella B. Eregie^1^*, Isaac A. Sanusi^3^, Gueguim E.B. Kana^1^,** **Olaniran O.** **Ademola^2^**

^1^Discipline of Microbiology, School of Life Sciences, University of KwaZulu-Natal, Private Bag, X01, Scottsville 3209, Pietermaritzburg, South Africa

^2^Discipline of Microbiology, School of Life Sciences, University of KwaZulu-Natal Private Bag X54001, Westville Campus, South Africa

^3^Fort Hare Institute of Technology, University of Fort Hare, Private Bag X1314, Alice, 5700, South Africa

**Supplementary document 1**


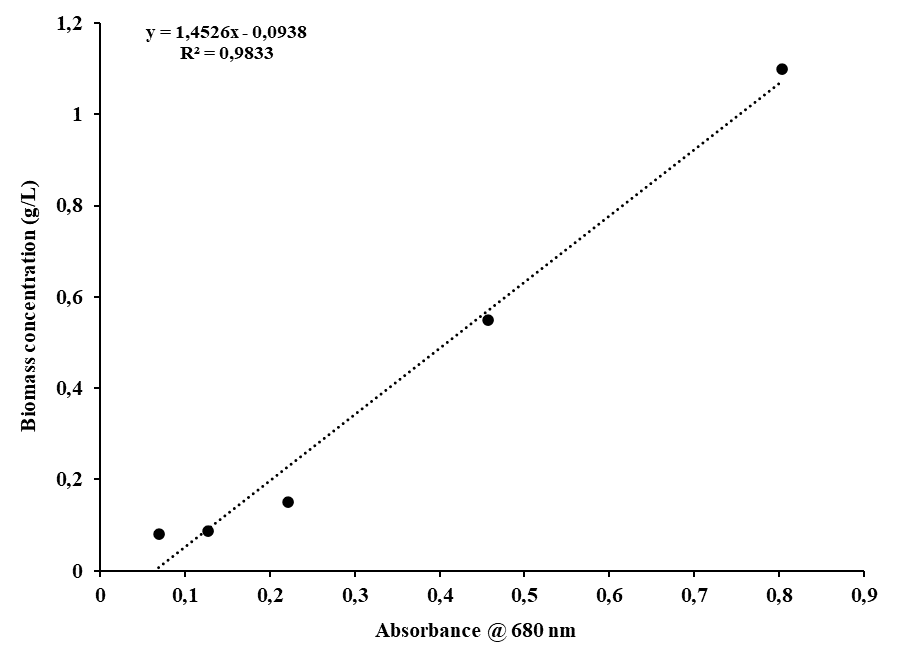


Fig. S1. The standard calibration curve used to calculate the wild-type microalgae cell dry weight

**Supplementary document 2**


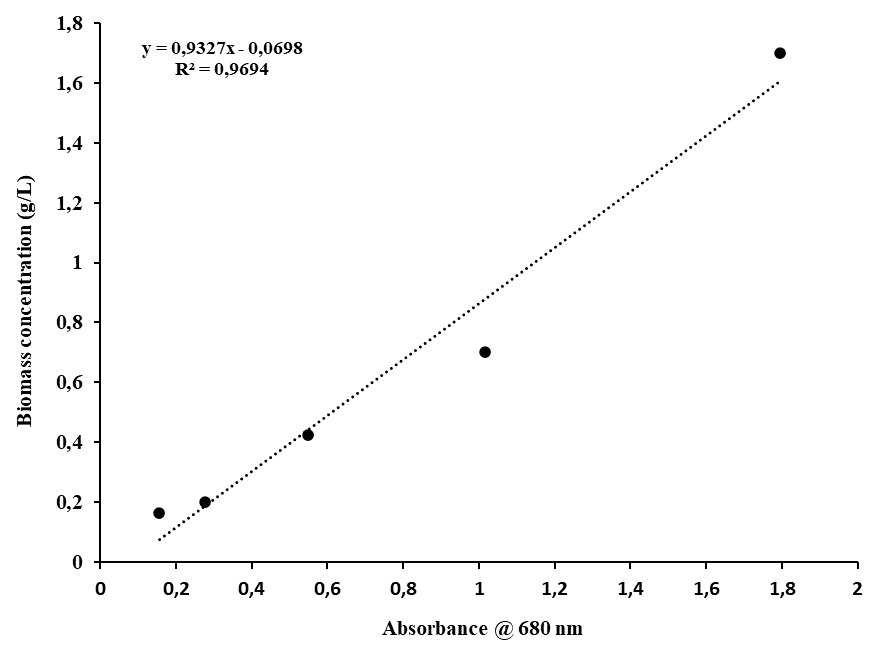


Fig. S2. The standard calibration curve used to calculate the UV exposed *Scenedesmus vacuolatus* cell dry weight

**Supplementary document 3**


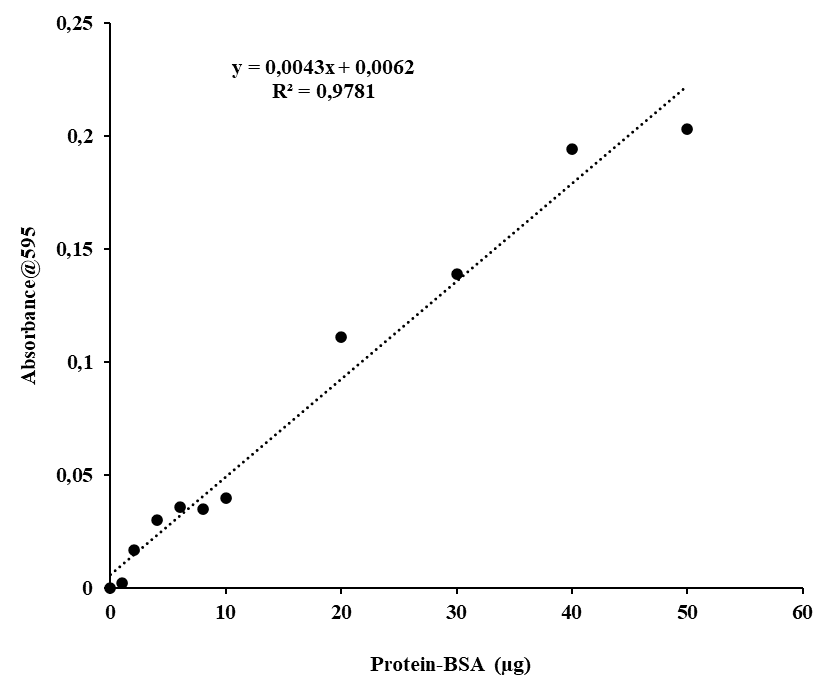


Fig. S3. The standard calibration curve used to deduce protein concentration of microalgae

**Supplementary document 4**


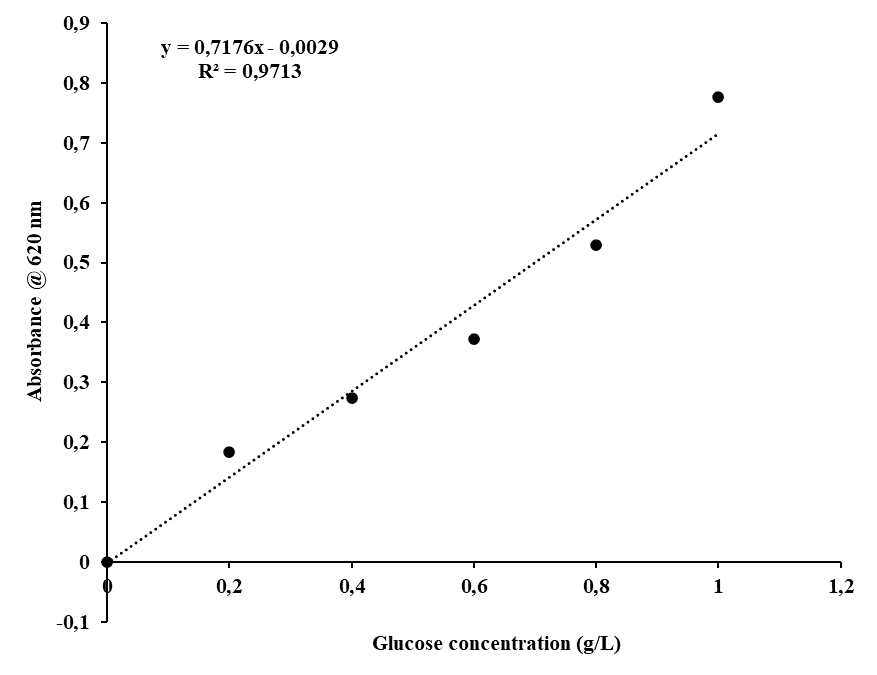


Fig. S4. The standard calibration curve used to deduce carbohydrate accumulation of microalgae

**Supplementary document 5**


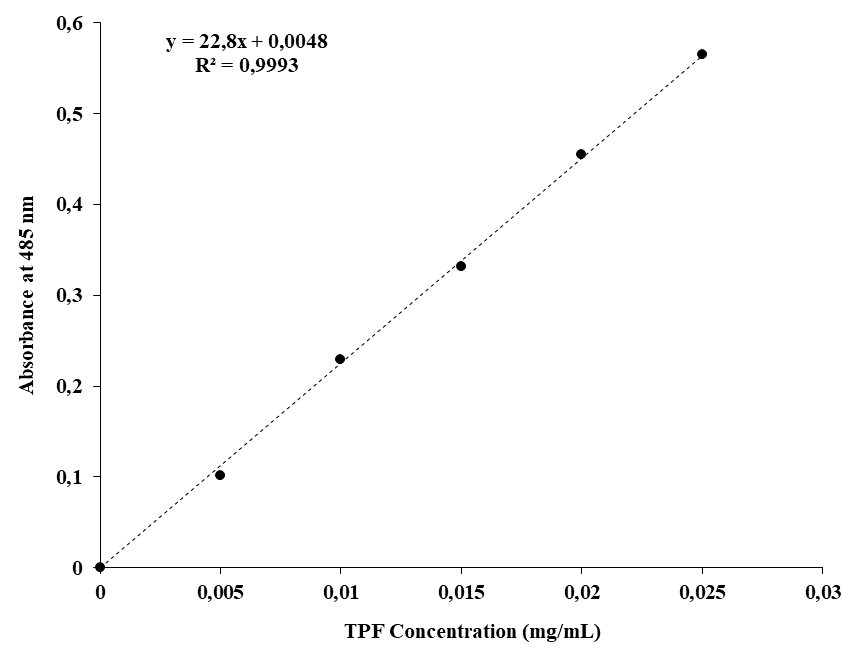


Fig. S5. The Triphenyl formazan (TPF) standard calibration curve used to calculate the unknown concentration of TPF produced.

**Supplementary document 6**


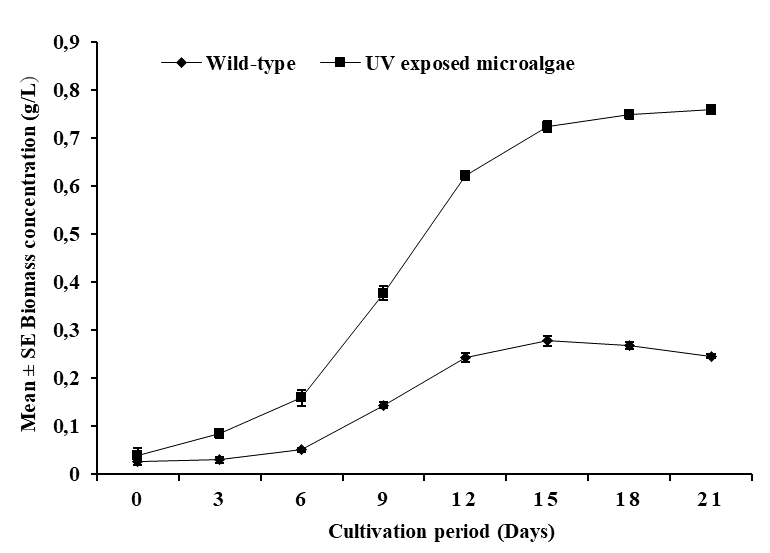


**a**


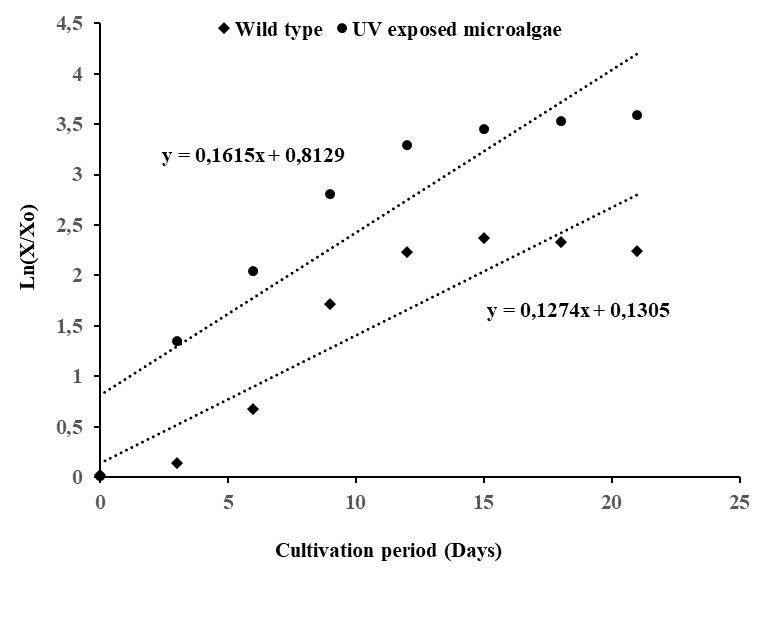


**b**

Fig. 6- shows the growth rate constant of microalgae after 15 days of cultivation. From the result obtained, ln X/Xo increased proportionally with increasing incubation time. The slope measures the rate of growth. In this case the greater the slope the higher the growth rate and biomass accumulation. The UV-exposed microalgae had the highest growth rate and biomass concentration and the steepest slope during the log phase (0.162), compared to the wild-type (0.127).

**Supplementary document 7**


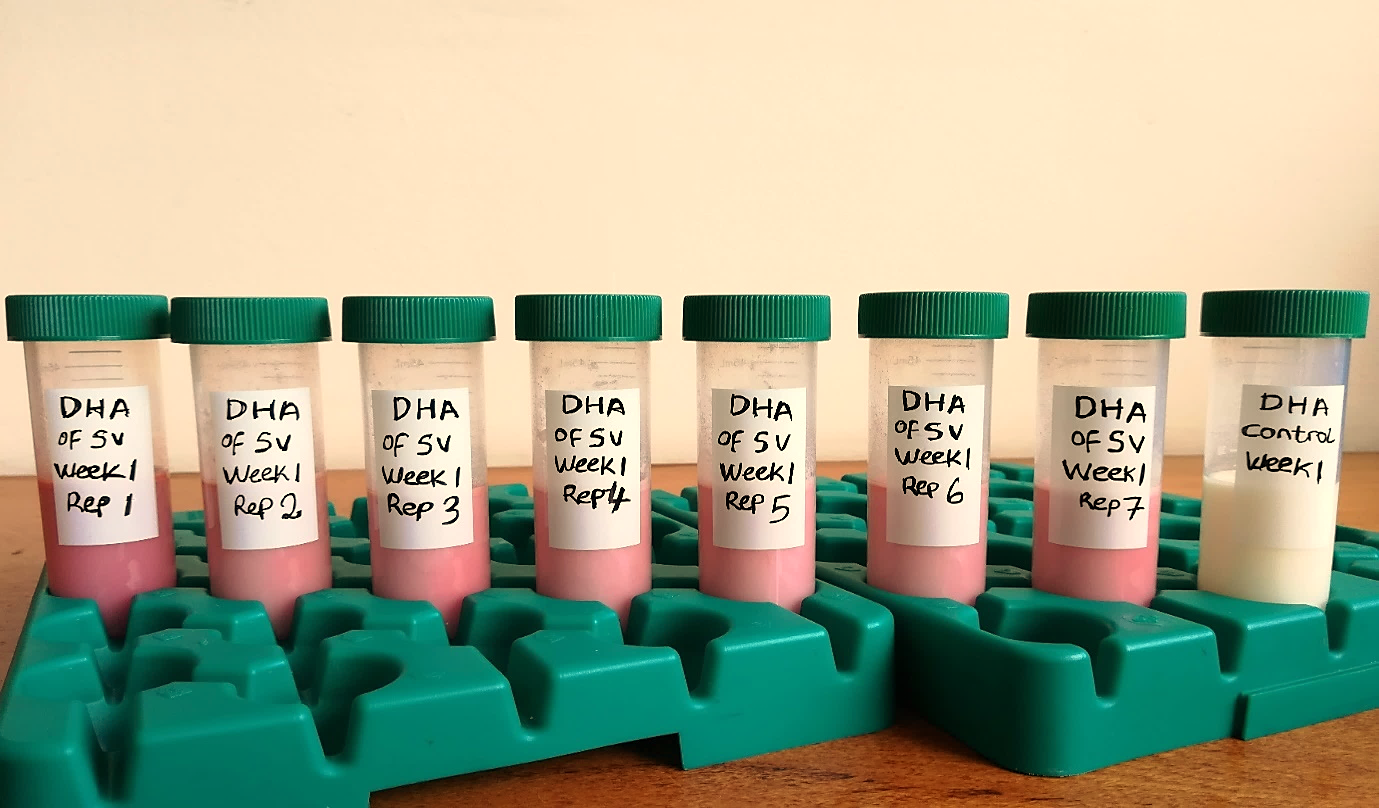

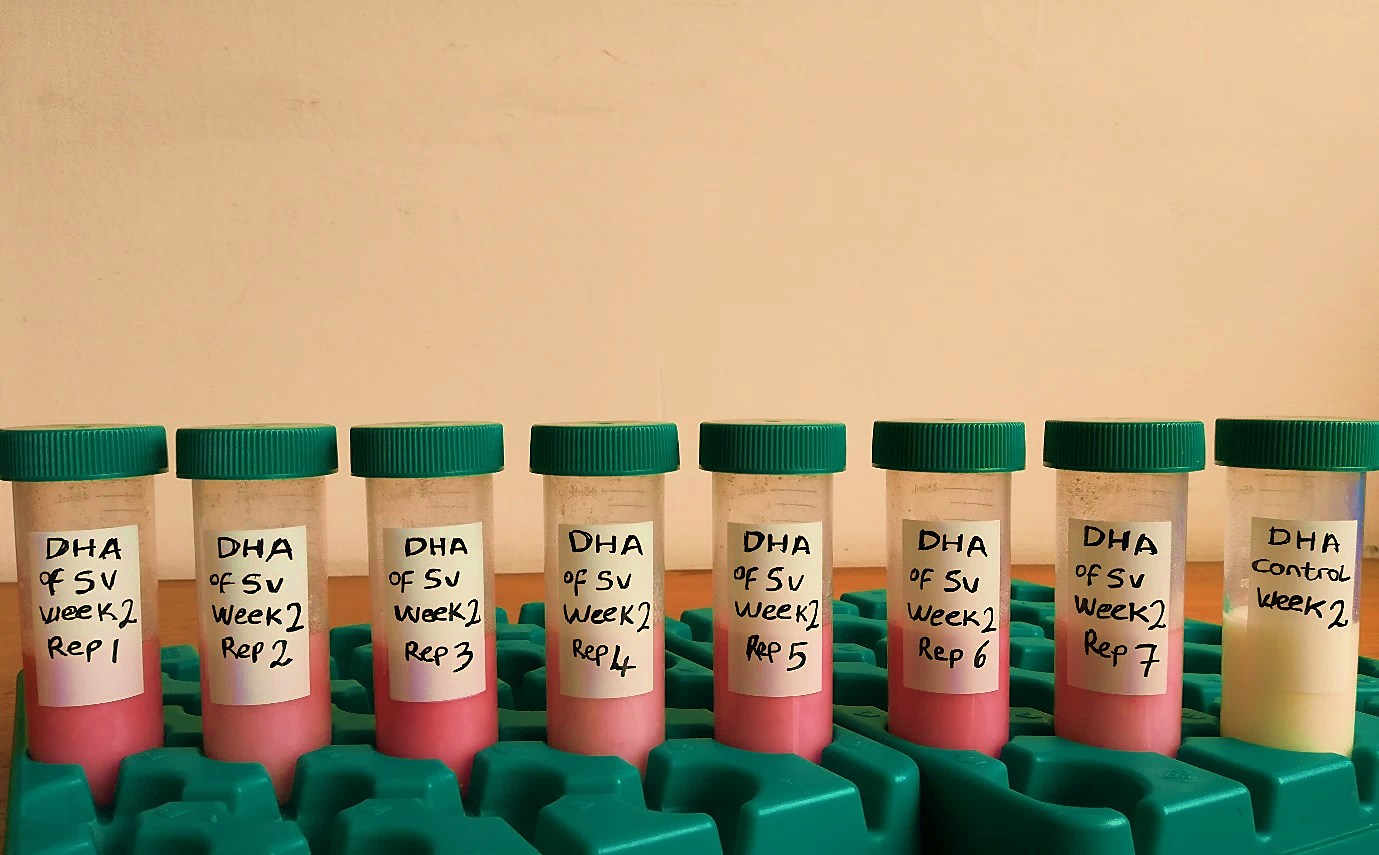

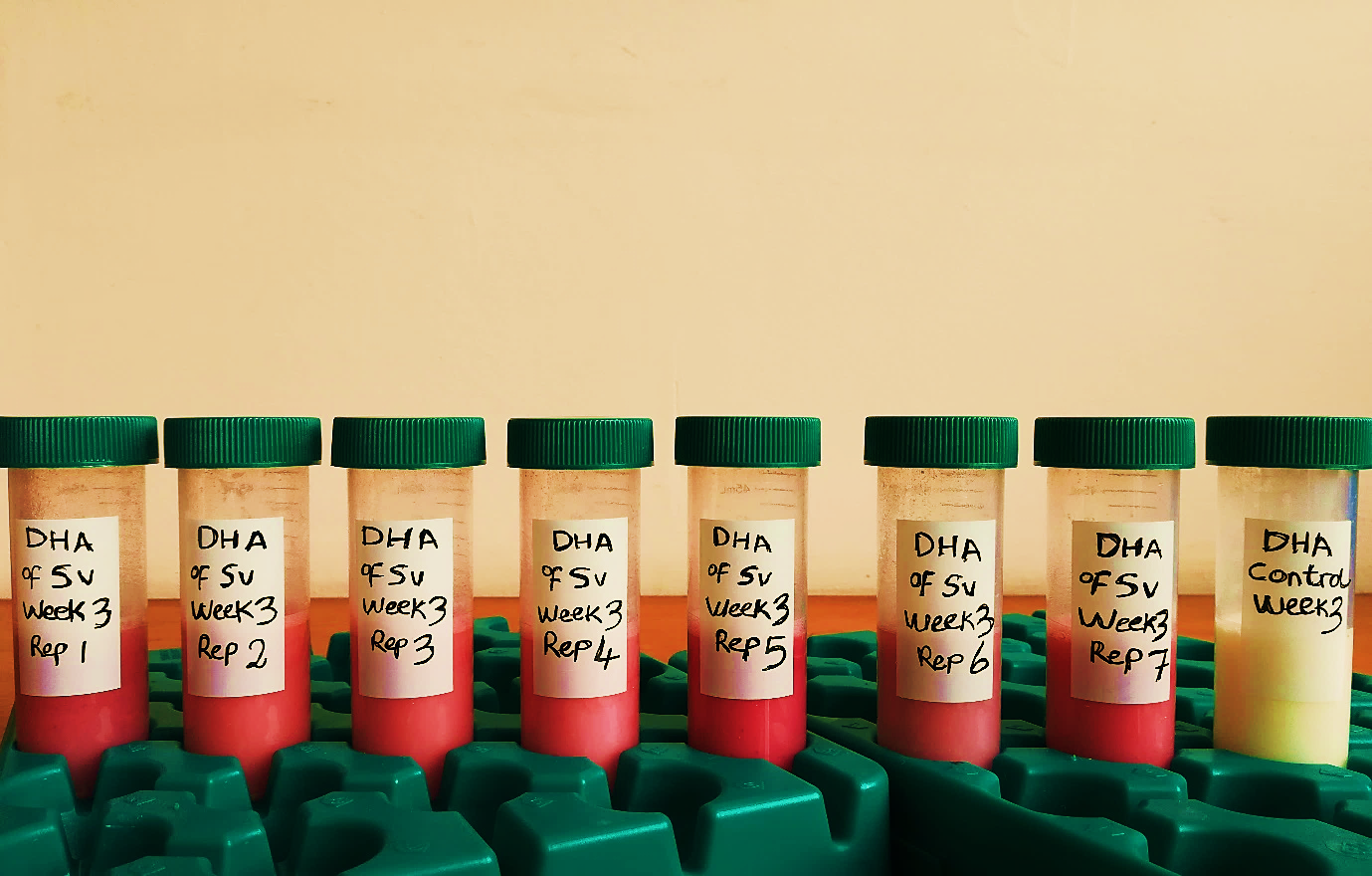

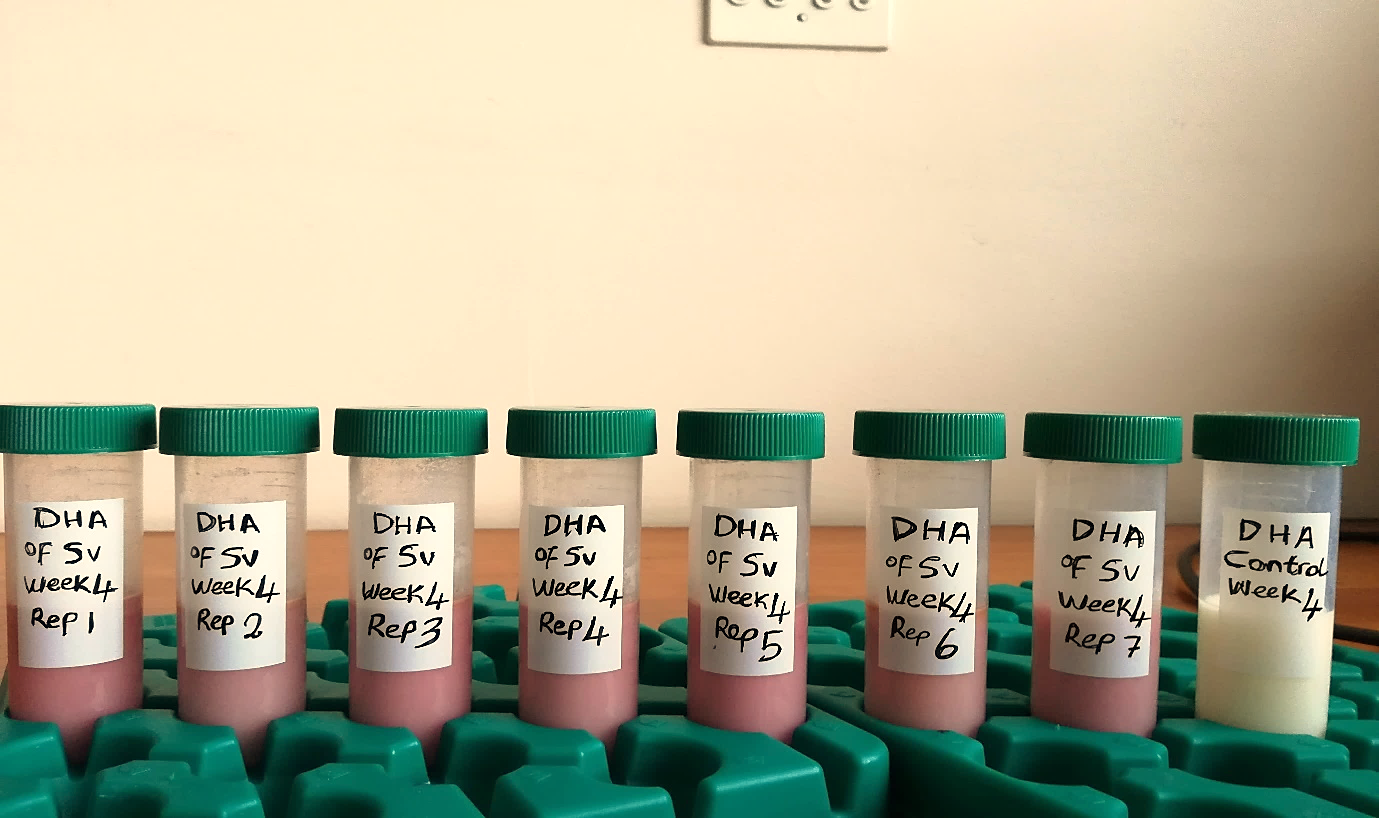

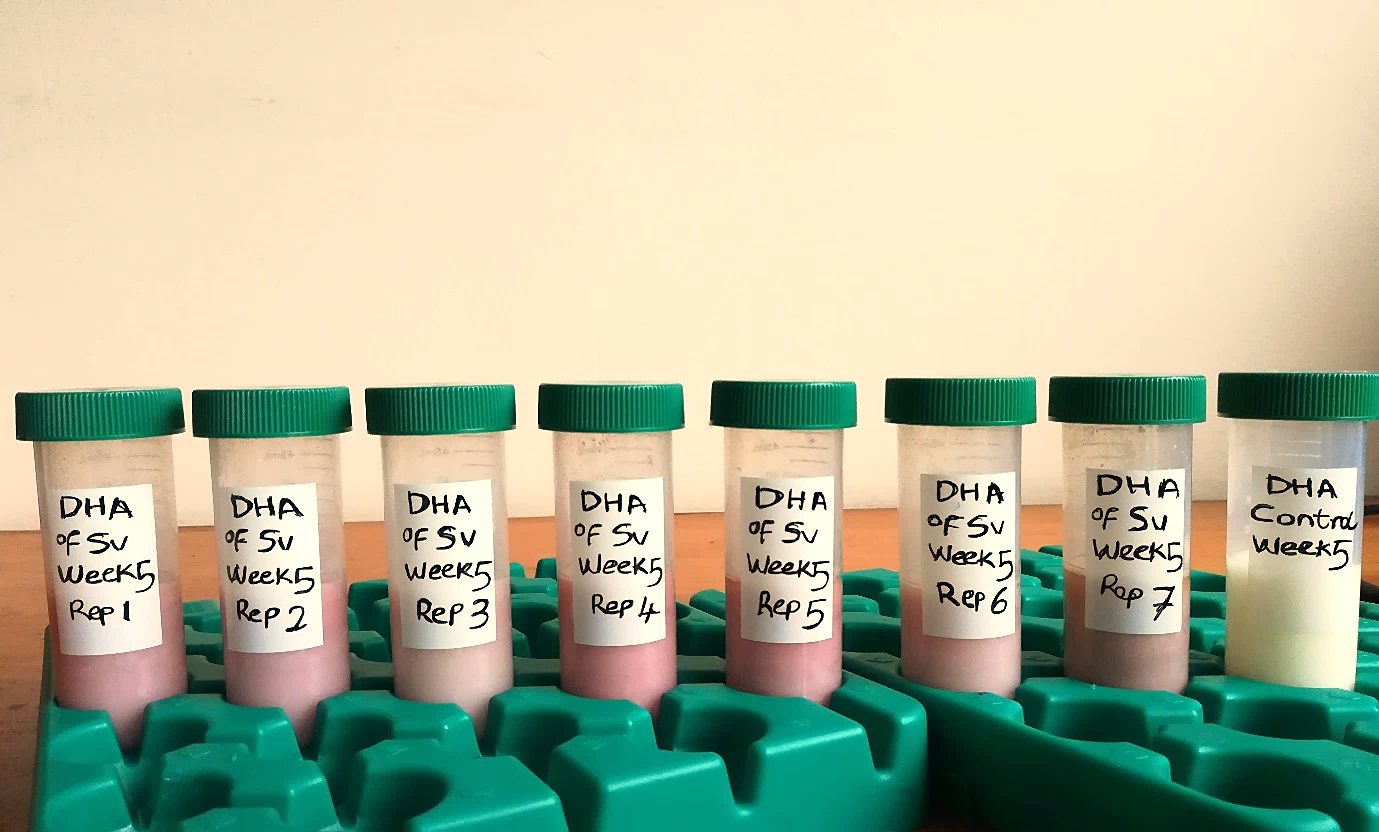


**Fig. S7:** Dehydrogenase activity of wildtype microalgae in spent coolant waste. The pink color represents the TPF produced. A show a light pink color at week one, followed by B with pink color at week two. C show a dark pink color with high concentrations of TPF at week three. pink color was observed at D week four and week five a pale pink color was observed indicating a decrease in TPF production.

**A**

**E**

**D**

**C**

**B**

**Supplementary document 8**


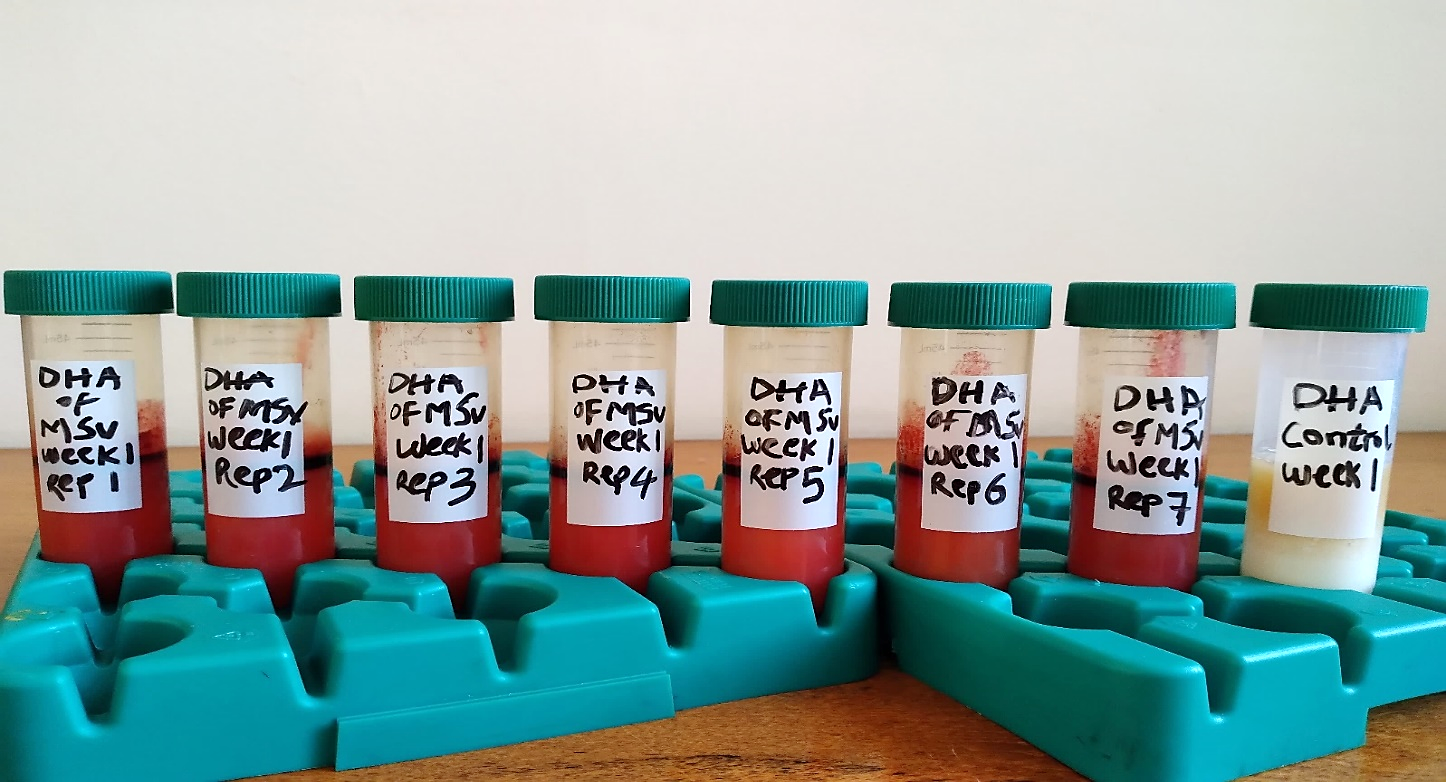

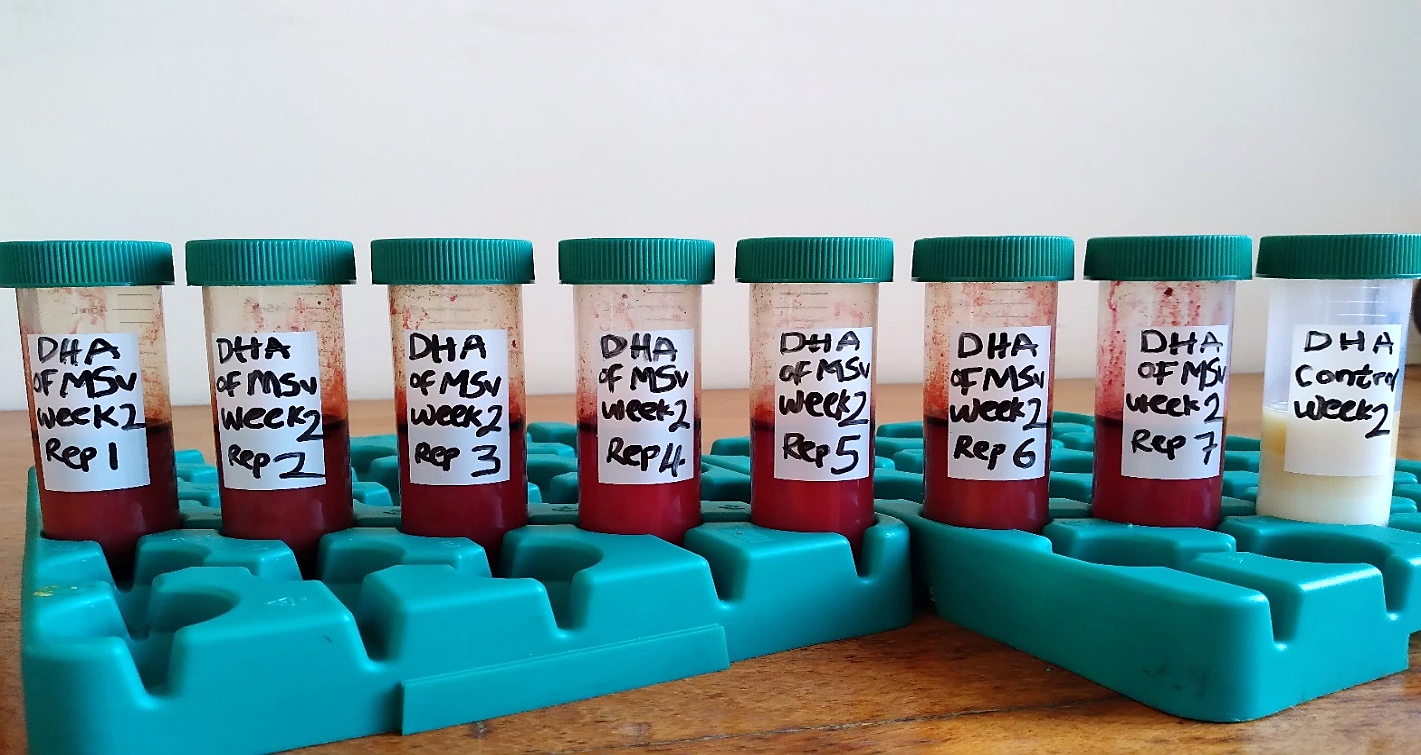

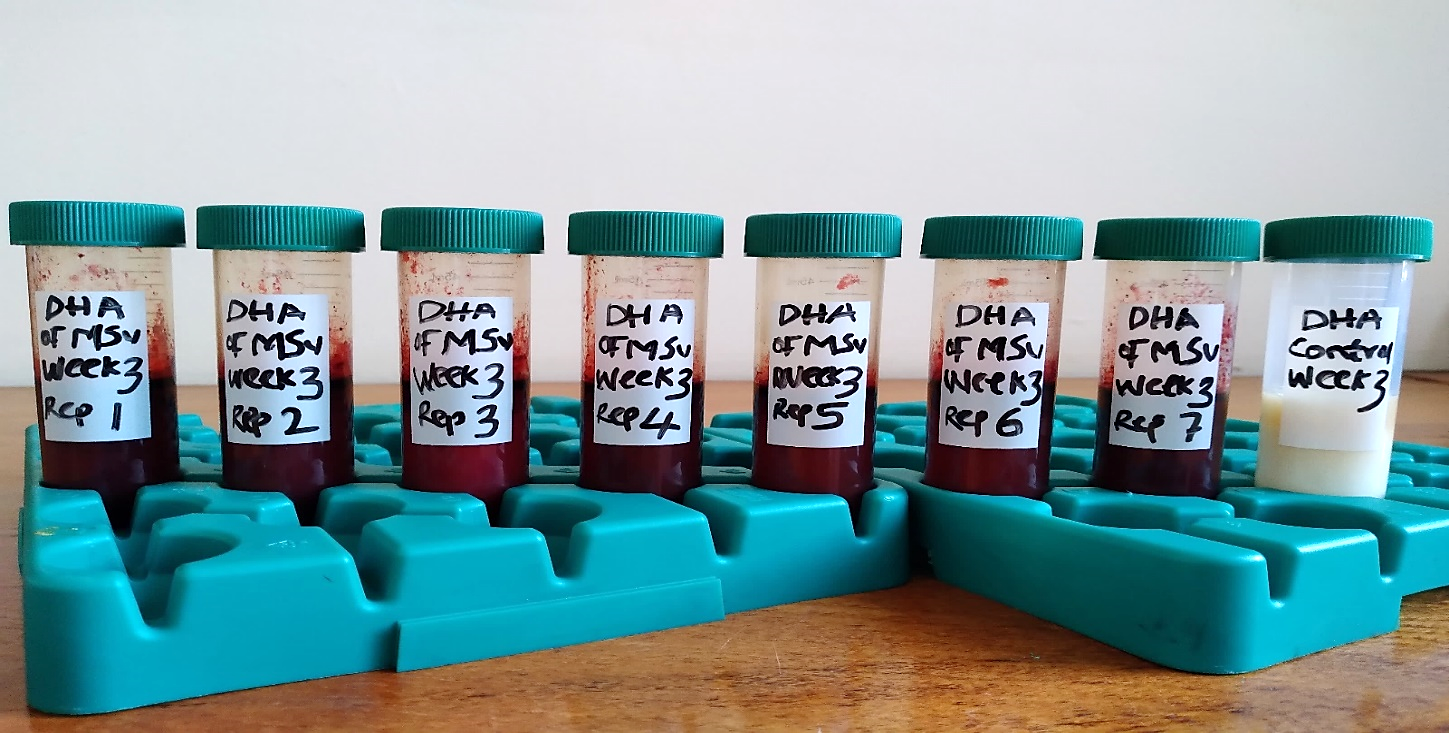

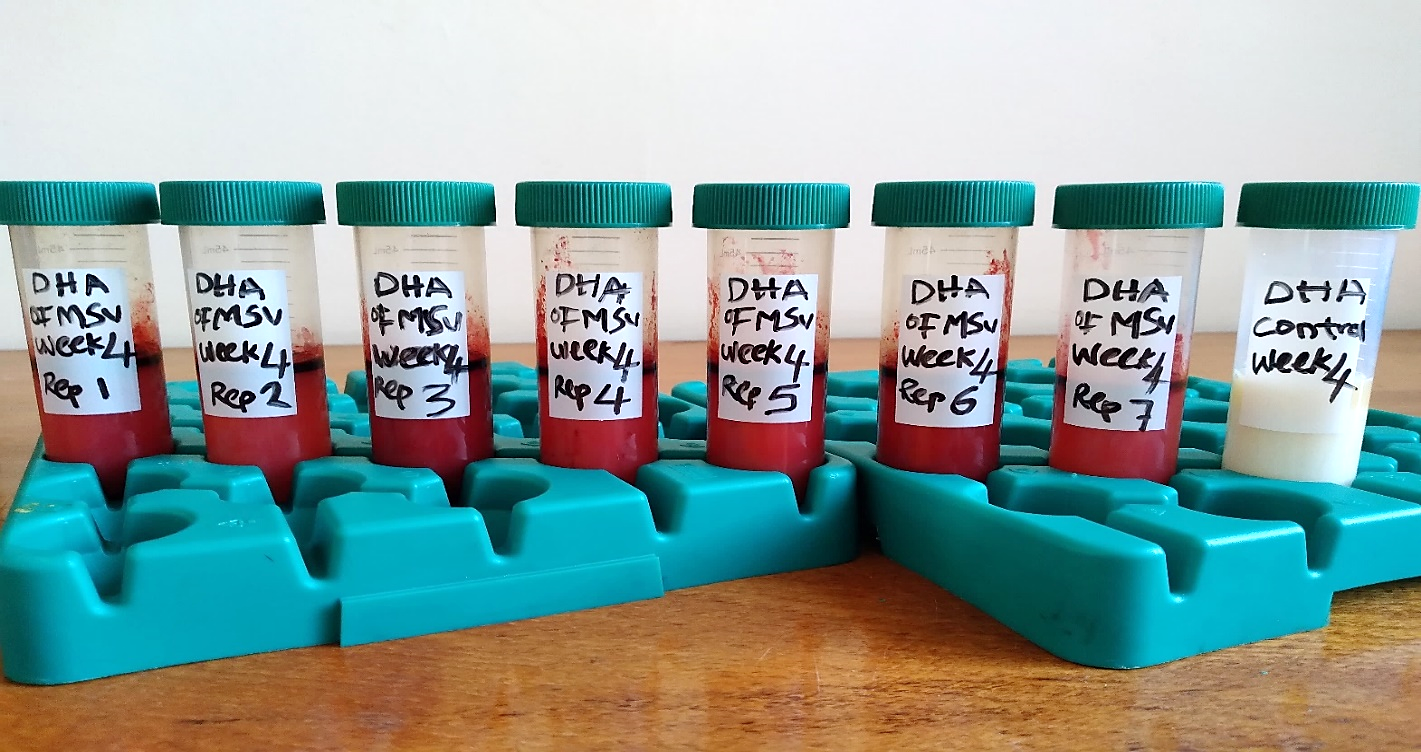

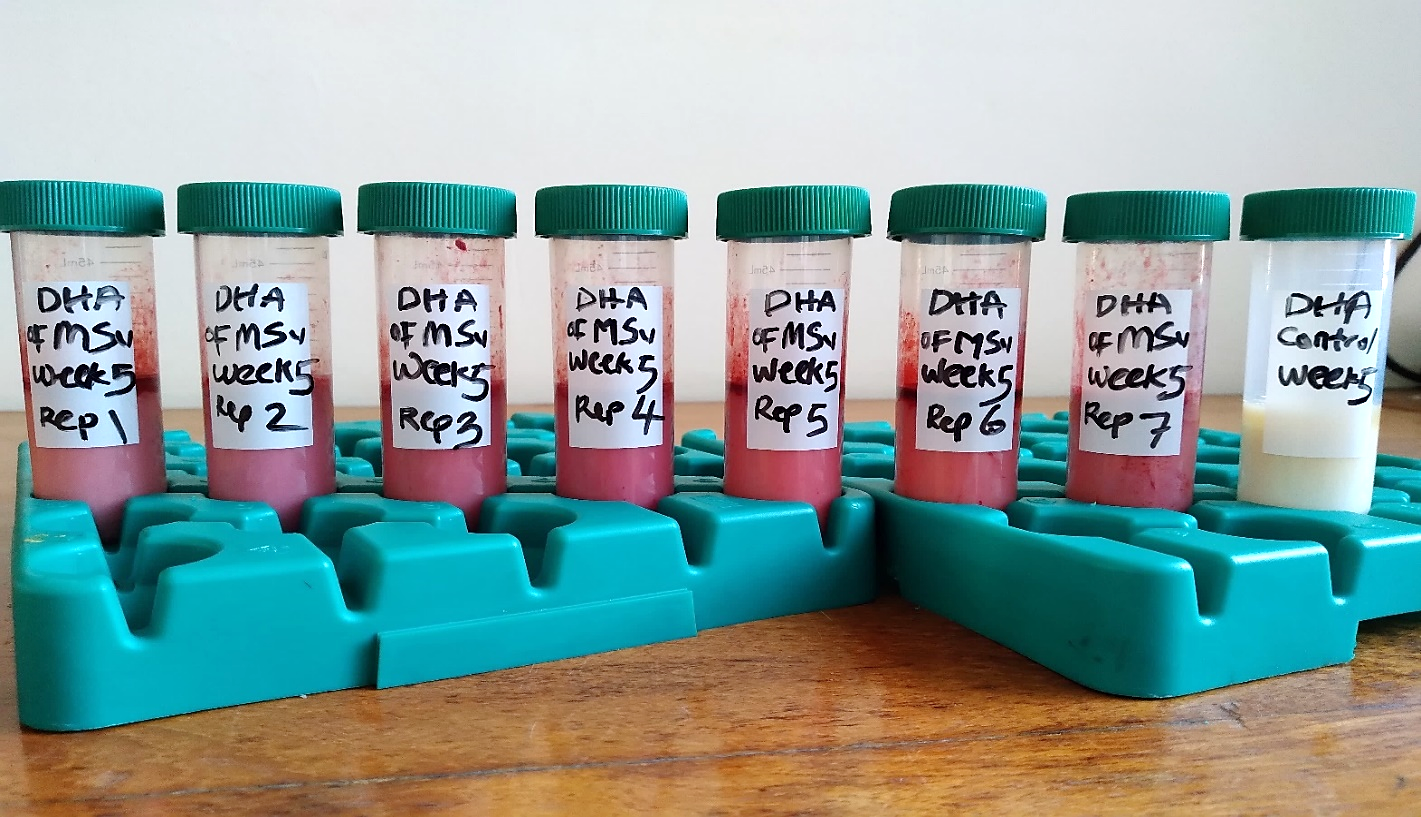


**Fig S8**: Dehydrogenase activity of UV exposed *S. vacuolatus* in spent coolant waste. The pink color represents the TPF produced. A show a pink color at week one, followed by B with dark pink color with high production of TPF at week two. C show a very dark pink color with higher production of TPF at week three. Pink color was observed at D week four and week five a light pink color was observed indicating a decrease in TPF production.

**A**

**E**

**D**

**C**

**B**

**Supplementary document 9**

**Table 1**: Hydrocarbon compounds present in spent coolant waste (SCW) abiotic control

| Alkanes compounds | **Formula/Isomers** | **Alkenes compounds** | **Formula/Isomers** | **Monoaromatic compounds** | | **Formula/Isomer** |
| --- | --- | --- | --- | --- | --- | --- |
| Hexane,2,3-dimethyl | C8H18 3 | 2-Undecene,2,5-dimethyl | C13H26 | p-Xylene | | C8H10 |
| Cyclohexane,1,3-dimethyl-cis | C8H16 | 1-Undecene,7-methyl | C12H24 | Benzene,1-ethyl-2-methyl | | C9H12 |
| Cyclopentane,1-ethyl-3-methyl-cis | C8H16 | 8-Heptadecene,1-chloro- | C17H33Cl | Benzene, (1,3,3-trimethylnonyl) | | C18H30 |
| 1-Ethyl-2-(4-methylpentyl) cyclopentane | C13H26 | Tricyclo (2,6) undeca-2(6),3-diene,11-methyl | C11H10O2 | Benzene,1-ethyl-2,4-dimethyl | | C10H14 1 |
| Nonane | C9H20 4 | 1,3-Cyclopentadiene,1,2,3,4-tetramethyl-5-methylene | C10H14 | Benzene,2-(2-butenyl)-1,3,5-trimethyl | | C13H18 |
| 1-Ethyl-4-methylcyclohexane | C9H18 1 | 3-Hexene, 3-ethyl-2,5-dimethyl- |  |  | |  |
| Decane | C10H22 4 | 2,4,4,6-Tetramethyl-6-phenyl-1-heptene |  | 4-Ethylbenzylamine, N, N-diheptyl | | C23H41N |
| Cyclohexane,1,1-dimethyl-2-propyl | C11H22 1 | **Organic acids/other compounds** |  | **Alcohols** | |  |
| Undecane | C11H24 4 | Sulfurous acid,2-ethylhexylhexadecyl ester | C14H50O3S | 1-Undecanol | | C11H24O |
| Tritetracontane | C43H88 | Malonic acid, 2-heptyl tetradecyl ester | C24H46O4 | 2-Hexyl-1-octanol | | C14H30O |
| Cyclopentane,1,1'-ethylidenebis | C12H22 | m-Toluic acid,2-tetrahydrofurylmethyl ester | C11H14O2 | 1-Heptanol,2-propyl | | C10H22O |
| Hexadecane, 1-chloro- |  | 10-Chlorodecyl propyl carbonate |  | (+-)-trans-1-Isopropenyl-4-methyl-1,4-cyclohexanediol | |  |
| Cyclohexane, 1-ethyl-1-methyl- |  | 2-Butyloxycarbonyloxy-1,1,10-trimethyl-6,9-epidioxydecalin |  | n-Pentadecanol | | C15H32O |
| Cyclopropane,1-chloro-1-ethyl-2,2,3-trimethyl | C8H15Cl | **PAHs** |  | 1-Hexanol,5-methyl-2-(1-methyl ethyl) | | C10H22O |
| Dodecane | C12H26 4 | Naphthalene,1,2,3,4-tetrahydro-6-methyl | C11H14 | **Chlorinated compounds** | |  |
| Dodecane,3-cyclohexyl | C18H36 | Naphthalene,2-methyl | C11H10 1 | 1-Octadecanesulphonyl chloride | | C18H37ClO2S |
| Tridecane | C13H28 5 | Naphthalene,2,6-dimethyl | C12H12 2 | Behenyl chloride | | C22H45Cl |
| Heptacosane,1-chloro | C27H55Cl | Naphthalene,1,2,3,4-tetrahydro-2,7-dimethyl- | C12H16 |  | |  |
| Hexadecane,2,6,10,14-tetramethyl | C20H42 |  |  |  | |  |
| Cyclohexane,1,1,3-trimethyl- | C15H30 |  |  |  |  | |
| Tetradecane | C14H30 4 |  |  |  |  | |
| Hexadecane,7,9-dimethyl | C18H38 |  |  |  | |  |
| Cyclohexane, octyl | C14H28 |  |  |  | |  |
| Heptadecane | C17H36 |  |  |  | |  |
| Pentadecane | C15H32 4 |  |  |  | |  |
| Hexadecane | C16H34 2 |  |  |  | |  |

The table shows the hydrocarbon compounds with their molecular formular, and isomer present in the spent coolant waste.

**Supplementary document 10**

**Table 2a**: Hydrocarbon compounds detected after week one, two, and three of treatment of SCW by wild type microalgae

| **Week 1** |  | **Week 2** |  | **Week 3** |  |
| --- | --- | --- | --- | --- | --- |
| **Alkanes Compounds** | **Formula** | **Alkanes Compounds** | **Formula** | **Alkanes compounds** | **Formula** |
| Hexane,2,2,5-trimethyl- | C9H20 | Cyclopentane,1,2,4-trimethyl- | C8H16 | Heptane,2,2-dimethyl- | C9H20 |
| Cyclopentane,1,2,4-trimethyl- | C8H16 | Heptane,2,2-dimethyl- | C9H20 | Hexadecane,2,6,11,15-tetramethyl | C20H42 |
| Pentadecane,2,6,10-trimethyl- | C18H38 | Cyclohexane,1,1,4,4-tetramethyl- | C10H20 | Cyclohexane,1,2-dimethyl-, trans- | C8H16 |
| Eicosane,10-methyl- | C21H44 | Nonane,4-methyl- | ‎C10H22 | Heptadecane,2,6,10,15, tetramethyl | C21H44 |
| Tetradecane,2,6,10-trimethyl- | C17H36 | Dodecane,2,7,10-trimethyl- | C15H32 | 2-Cyclohexylnonadecane | C25H50 |
| Tetradecane,4,11-dimethyl- | C16H34 | Pentadecane, 3-methyl- | C16H34 | Octane,3,3-dimethyl- | ‎C10H22 |
| Heptadecane,2,6,10,15-tetramethyl- | C21H44 | 2-methylhexacosane | C27H56 | Dodecane,2,7,10-trimethyl- | C15H32 |
| Heneicosane,11-(1-ethylpropyl)- | C26H54 | Undecane,4,8-dimethyl- | C13H28 | Tetradecane,2,6,10-trimethyl- | C17H36 |
| **Alcohols** |  | 10-Methylnonadecane | C20H42 | Cyclooctane,1,2-dimethyl- | C10H20 |
| n-Tridecan-1-ol | C13H28O | **Alcohols** |  | **Alcohols** |  |
| 1-Heptacosanol | C27H56O | 1-Decanol,2-octyl- | C18H38O | 1-Decanol,2-methyl- | C11H24O |
| n-Tetracosanol-1 | C24H50O | n-Heptadecanol-1 | C17H36O | 1-Dodecanol, 2-hexyl- | C18H38O |
| **Monoaromatic compounds** |  | 11-Methyldodecanol | C13H28O | 7-Heptadecanol,7-methyl- | C18H38O |
| Benzene,1,3-dimethyl- | C8H10 | cis-1,2-Cyclododecanediol | C12H24O2 | Cholesta-8,24-dien-3-ol,4methyl | C26H46O |
| Benzene,1-ethyl-3-methyl- | C9H12 | **Monoaromatic compounds** |  | n-Nonadecanol-1 | C19H40O |
| Benzene,2-ethyl-1,4-dimethyl | C10H14 | Benzene,1,3-dimethyl- | C8H10 | n-Tridecan-1-ol | C13H28O |
| 1,2-Benzenedicarboxylic acid | C8H6O4 | 3-Trifluoromethylbenzoic acid, dodecyl ester | C20H28F4O | 9-Undecenol,2,10-dimethyl- | C13H26O |
| Benzhydrol ether | C26H22O | Benzeneacetic acid, 4-tridecyl ester | C21H34O2 | 1-Nonanol,4,8-dimethyl- | C11H24O |
| **PAHs** |  | Benzene,1,4-diethyl- | C10H14 | 1-Decanol,2-octyl- | C18H38O |
| Naphthalene,1-methyl | C11H10 | 1,4-Benzenediol,2,6-bis(1,1-dimethylethyl)- | C14H22O2 | Z, E-3,13-Octadecadien-1-ol | C18H34O |
| Naphthalene,1,3-dimethyl- | C12H12 | 1,2-Benzenedicarboxylic acid, butyl 2-methylpropyl | C16H22O4 | **Fatty acids/other compounds** |  |
| 1H-Indene,2,3-dihydro-4-methyl- | C10H12 | **PAHs** |  | Malonic acid, neopentyl tridecyl ester | C21H40O4 |
| 1,2,3,4-tetrahydro-1,2-naphthalenediol | C10H12O2 | 1H-Indene,2,3-dihydro-4-methyl- | C10H12 | Dodecanoic acid, methyl ester | C13H26O2 |
| 1,2-Naphthalenediol | C10H8O2 | Decahydro-4,4,8,9,10-pentamethyl naphthalene | C15H28 | Fumaric acid, ethyl heptadecyl ester | C23H42O4 |
| 1-Naphthol,1,2,3,4-tetrahydro-2-methyl | C11H14O | 1-Naphthol,1,2,3,4-tetrahydro-2-methyl- | C11H14O | Hydrazine carboxylic acid | C8H10N2O2 |
| **Fatty acid/other compounds** |  | **Fatty acids/other compounds** |  | Trichloroacetic acid,1-cyclopentyl ethyl ester | C9H13Cl3O |
| Dichloroacetic acid, 2-tridecyl ester | C15H28Cl | Decadienedioic acid diethyl ester | C14H26O4 | Palmitoleic acid | C16H30O2 |
| Sulfurous acid, dicyclohexyl ester | C12H22O3S | Palmitoleic acid | C16H30O2 | Acetic acid, trifluoro-, dodecyl ester | C14H25F3O2 |
| Palmitoleic acid | C16H30O2 | Carbonic acid, ethyl isobutyl ester | C7H14O3 | Tetradecanoic acid, 2,3-dihydroxypropyl | C17H34O4 |
| Malonic acid,2-heptyltetradecyl ester | C24H46O4 | Octadecanoic acid | C18H36O2 | Tridecanoic acid | C13H26O2 |
| Fumaric acid, ethyl heptadecyl ester | C23H42O4 | Trichloroacetic acid,1-cyclopentyl ethyl ester | C9H13Cl3O |  |  |
| Acetic acid, trifluoro-, dodecyl ester | C14H25F3O2 | Octanoic acid, tetradecyl ester | C22H44O2 |  |  |
| Tetradecanoic acid, 2,3-dihydroxypropyl | C17H34O4 |  |  |  |  |

The table shows the list of extracellular hydrocarbons present with their molecular formula after wild-type microalgae treatment of SCW.

**Table 2b**: Hydrocarbon compounds detected after week four and five of treatment of SCW by wild type microalgae

| **Week 4** |  | **Week 5** |  |
| --- | --- | --- | --- |
| **Alkanes Compounds** | **Formula** | **Alcohols** | **Formula** |
| Hexane,3,3,4-trimethyl- | C9H20 | 1-Decanol,2-methyl- | C11H24O |
| Decane,3,6-dimethyl- | ‎C12H26 | Tridecanol,2-ethyl-2-methyl | C16H34O |
| 3-Hexane,3-ethyl-2,5-dimethyl | C10H22 | 2-Isopropyl-5-methyl-1-heptanol | ‎C11H24O |
| Heptadecane,2,6,10,15-tetramethyl- | C21H44 | 11-Methyldodecanol | C13H28O |
| Heneicosane,5-methyl | C22H46 | 1-Dodecanol, 2-octyl- | C18H38O |
| Tetradecane,2,6,10-trimethyl- | C17H36 | 2-Tridecen-1-ol, (E)- | C13H26O |
| Undecane,4,8-dimethyl- | C13H28 | **Fatty acids/other compounds** |  |
| Dodecane,2,6,11-trimethyl- | C15H32 | Pentadecanoic acid | C15H30O2 |
| Heptadecane,2-methyl- | C18H38 | Octanoic acid, hexadecyl ester | C24H48O2 |
| **Alcohols** |  | Methyl tetradecanoate | C15H30O2 |
| 1-Octanol,2-butyl- | C12H26O | 4-Bromobutanoic acid, heptadecyl ester | C21H41BrO2 |
| 1-Nonanol,4,8-dimethyl- | C11H24O | Trichloroacetic acid,1-cyclopentyl ethyl ester | C9H13Cl3O2 |
| n-Tridecan-1-ol | C13H28O | Tridecanoic acid, methyl ester | C14H28O2 |
| 11-Methyldodecanol | C13H28O | Fumaric acid, 8-chlorooctyl hexyl ester | C18H31ClO4 |
| Cyclododecanemethanol | C13H26O | Methoxyacetic acid, 4-hexadecyl ester | C19H38O3 |
| 1-Decanol, 2-octyl- | C18H38O | Dichloroacetic acid, tridecyl ester | C15H28Cl2O2 |
| n-Tetracosanol-1 | C24H50O | Palmitoleic acid | C16H30O2 |
| **Fatty acids/other compounds** |  |  |  |
| Decanoic acid | C10H20O2 |  |  |
| Palmitoleic acid | C16H30O2 |  |  |
| Carbonic acid, ethyl isobutyl ester | C7H14O3 |  |  |
| Hexadecanoic acid | C16H32O2 |  |  |
| Nonanoic acid | C9H18O2 |  |  |
| Dodecanoic acid, methyl ester | C13H26O2 |  |  |
| Dichloroacetic acid, tridecyl ester | C15H28Cl2O2 |  |  |
| Formic acid,2-ethylhexyl ester | C9H18O2 |  |  |
| 4-Bromobutanoic acid, heptadecyl ester | C21H41BrO2 |  |  |

The table shows the list of extracellular hydrocarbons present with their molecular formula after wild-type microalgae treatment of SCW.

**Supplementary document 11**

**Table 3a**: Hydrocarbon compounds detected after week one, two and three of treatment of spent coolant waste by UV exposed *S. vacuolatus*

| **Week 1** |  | **Week 2** |  | **Week 3** |  |
| --- | --- | --- | --- | --- | --- |
| **Alkanes Compounds** | **Formula** | **Alkanes Compounds** | **Formula** | **Alkanes compounds** | **Formula** |
| Pentadecane,2,6,10,14-tetramethyl | C16H34 | Cyclotridecane | C13H26 | Undecane,4,7-dimethyl- | C13H28 |
| Decane,4-ethyl | C12H26 | Hexadecane,4-methyl- | C17H36 | Dodecane,2,6,11-trimethyl- | C15H32 |
| Nonane,2,3-dimethyl | ‎C11H24 | Tetradecane,4,11-dimethyl- | C16H34 | Pentadecane,3-methyl- | C16H34 |
| Dodecane,2,6,11-trimethyl | C15H32 | Hexadecane,2,6,11,15-tetramethyl- | C20H42 | Heptadecane,2,6,10,15, tetramethyl | C21H44 |
| Hexadecane,2,6,11,15-tetramethyl | C20H42 | 2,2-Dimethyldodecane | C14H30 | Tetrapentacontane,1,54-dibromo- | C54H108Br2 |
| Heptadecane,2,6,10,15-tetramethyl | C21H44 | Heptadecane,2,6-dimethyl- | C19H40 | Hexadecane,7,9-dimethyl | C18H38 |
| **Alcohols** |  | Undecane,2,4-dimethyl | C13H28 | **Alcohols** |  |
| 1-Decanol, 2-octyl | C18H36O | **Alcohols** |  | n-Tridecan-1-ol | C13H28O |
| 9,12-Octadecadien-1-ol | C18H34O | n-Tridecan-1-ol | C13H28O | Cyclohexanol,2,4-dimethyl- | C8H16O |
| n-Tetracosanol-1 | C24H50O | Cyclohexanol,2,4-dimethyl- | C8H16O | 1-Hexadecanol,3,7,11,15-tetramethyl | C20H40O |
| **Monoaromatic compounds** |  | 1-Hexadecanol,3,7,11,15-tetramethyl | C20H40O | **Ketones** |  |
| 1,2-Benzenediol, 4-[2-[[3-(4-hydroxyphenyl)-1-methylDihydroartemisininoxymethyl benzoic acid | C18H23NO3  C23H30O7 | 1-Decanol,2-hexyl  6,10,13-Trimethyl tetradecanol | C16H34O  C5H11ClO | 5,8-Tridecadione  4,4-Dimethyl-2-allylcyclohexanone | C19H34O2  C11H18O |
| Benzyl alcohol,4-methoxy-6-fluoro  Benzyloxy tridecanoic acid | C8H9FO2  C20H32O3 | n-Nonadecanol-1  Tetracontane-1,40-diol | C19H40O  C40H82O2 | 3,6-Undecandione  2-Pentadecanone, 6,10,14-trimethyl- | C17H36O  C18H36O |
| Phthalic acid  1,2-Benzenedicarboxylic acid, butyl,2-methyl propyl ester | C8H6O4  C16H22O4 | 1,4-cyclohexanediol  n-Nonadecanol-1 | C6H12O2  C19H40O | 1,3-Cyclohexanedione,5,5-dimethyl-2,2-dipropyl- | C13H24O2 |
| Phthalic acid, di(2-propylpentyl) ester  2,5-di-tert-Butyl-1,4-benzoquinone | C24H38O  C14H20O2 | n-Tetracosanol-1  1-Dodecanol | C24H50O  C12H26O | **Fatty acids/other compounds** |  |
| **PAHs** |  | **Fatty acids/other compounds** |  | Pentadecanoic acid,3-methyl-, methyl ester  Octadecanoic acid,9,10-epoxy-, isopropyl | C17H34O2  C19H36O3 |
| 2-Ethyl-1-methyl-1,2,3,4-tetrahydro-1,2-naphthalenediol | 13H18O2 | Cyclohexane carboxylic acid,4-pentyl-,2,3-dicyano-4-ethoxyphenyl ester | C20H24N2O3 | Cyclopentane carboxylic acid | C6H10O2 |
| 1-Naphthol,1,2,3,4-tetrahydro-2-methyl  1,5-Naphthalenediol, decahydro | C11H14O  C10H18O2 | Acetic acid,7,7-dimethyl-2-oxobicyclohept-1-ylmethyl ester | C12H18O3 | Octadecanoate | C18H35O2 |
| 3,4-dihydro-3-methylnaphthalen-1-one  2-Naphthoic acid, 3-methoxy-4-methyl | C11H12O  C25H20O6 | Dodecanoic acid, 2,2,2-trifluoroethyl ester  Nonahexacontanoic acid | C14H25F3O2  C69H138O2 | Hexadecanoate | C16H31O2 |
| 1-Naphthaleneacetic acid  2-Naphthalenecarboxylic acid, 4,4'-methylenebis | C12H10O2  C23H14Li2O6 | cis-13-Eicosenoic acid  Alpha-hydroxyisocaproic acid | C18H36O  C6H12O3 | 6-Octadecenoic, acid, methyl ester, (Z)- | C19H36O2 |
| **Fatty acids/other compounds** |  | Octadecanoic acid  Linoleic acid | C18H36O2  C18H32O2 | Palmitoleic acid | C16H30O2 |
| Tetradecanoic acid | C14H28O2 | Palmitoleic acid | C16H30O2 | 8-Octadecenoic acid | C18H34O2 |
| Pentadecanoic acid | C15H30O2 | Eicosapentaenoic acid | C20H30O2 | hexanedioic acid beta-citronellyl octyl ester | C26H46O4 |
| Cyclopentane carboxylic acid,3-tridecyl ester | C20H38O2 | Dodecanoic acid, cyclohexyl ester | C18H34O2 | Undecanoic acid | C11H22O2 |
| Formic acid,2-ethylhexyl ester | C9H18O2 | Dichloroacetic acid, heptadecyl ester | C19H36Cl2O2 |  |  |
| Cyclohexane carboxylic acid,4-pentyl-,2,3-dicyano-4 | C22H28N2O3 | Methoxyacetic acid, 4-hexadecyl ester | C19H38O3 |  |  |
| 6-Octadecenoic acid, methyl ester, (Z)- | C19H36O2 | 3-Methylcyclohexane carboxylic acid | C8H14O2 |  |  |
| Hexadecanoic acid, (3-bromoprop-2-ynyl) ester | C19H33BrO2 | Hexanedioic acid, bis(2-ethylhexyl) ester | C22H42O |  |  |
| Cyclohexane, carboxylic acid | C7H12O2 | Nonanoic acid, nonyl ester | C18H36O2 |  |  |

The table shows the list of extracellular hydrocarbons present with their molecular formula after mutagenized microalgal treatment of SCW.

**Table 3b**: Hydrocarbon compounds detected after week four and five treatment of SCW by UV exposed *S. vacuolatus*

| **Week 4** |  | **Week 5** |
| --- | --- | --- |
| **Fatty acids/other compounds** | **Formula** |  |
| Hexanoic acid | C6H12O2 | **Hydrocarbons not detected** |
| Heptanoic acid | C7H14O2 |  |
| Octanoic acid | C8H16O2 |  |
| Tridecanoic acid | C13H26O2 |  |
| Pentadecanoic acid | C15H30O2 |  |
| Acetic acid, trifluoro-, dodecyl ester | C14H25F3O2 |  |
| Tetradecanoic acid, 2,3-dihydroxypropyl ester | C17H34O4 |  |
| Hexadecanoic acid,2,3-bis(acetyloxy)propyl ester | C23H42O6 |  |
| n-Decanoic acid | C10H20O2 |  |
| Hexanedioic acid, dioctyl ester | C22H42O4 |  |
| alpha-Ketostearic acid | C18H34O3 |  |
| Octadecanoic acid,2,3-bis[(1-oxotetradecyl) oxy] propyl ester | C49H94O6 |  |
| 9-Oxononanoic acid | C9H16O3 |  |
| Dodecanoic acid | C12H24O2 |  |
| Octanedioic acid | C8H14O4 |  |
| Palmitoleic acid | C16H30O2 |  |
| Tridecanoic acid, methyl ester | C14H28O2 |  |
| Octadecanoic acid,9,10-epoxy-, isopropyl ester | C19H36O3 |  |
| Oleic acid | C18H34O2 |  |
| Cyclohexane carboxylic acid hydrazide | C7H14N2O |  |
| Palmitic acid | C16H32O2 |  |
| Decanoic acid,5,5-dimethyl-9-oxo-, methyl ester | C13H24O3 |  |

The table shows the list of extracellular hydrocarbons present with their molecular formula after mutagenized *S. vacuolatus* treatment of SCW.

**Supplementary document 12**

**GCMS chromatogram of hydrocarbons of SCW treated by the wild-type microalgae**


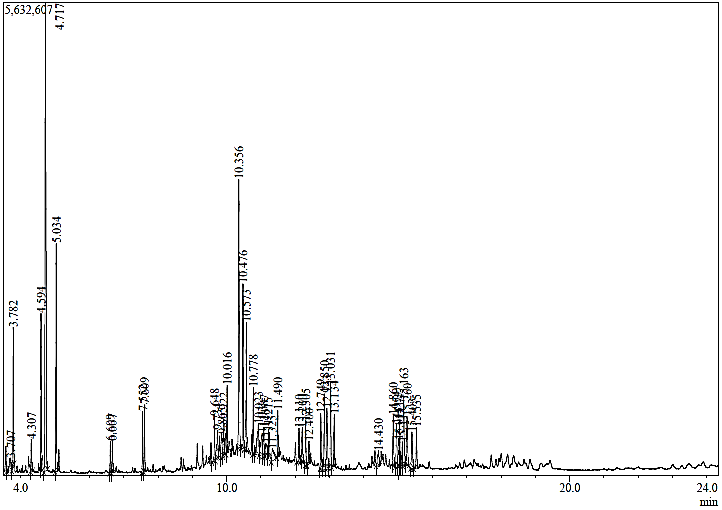

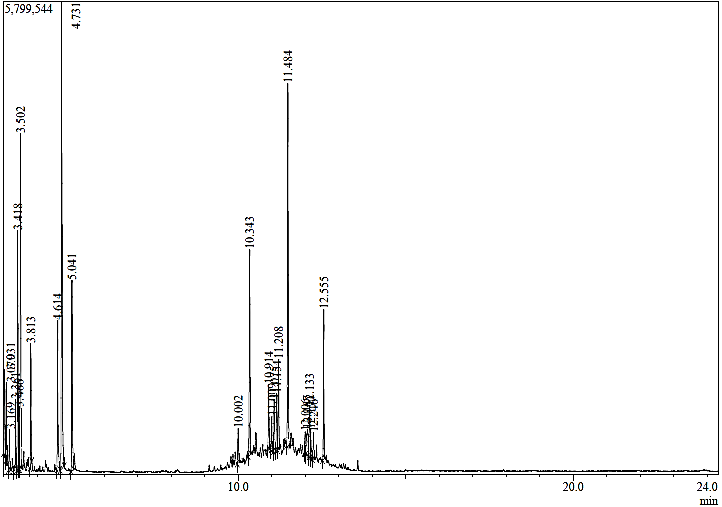

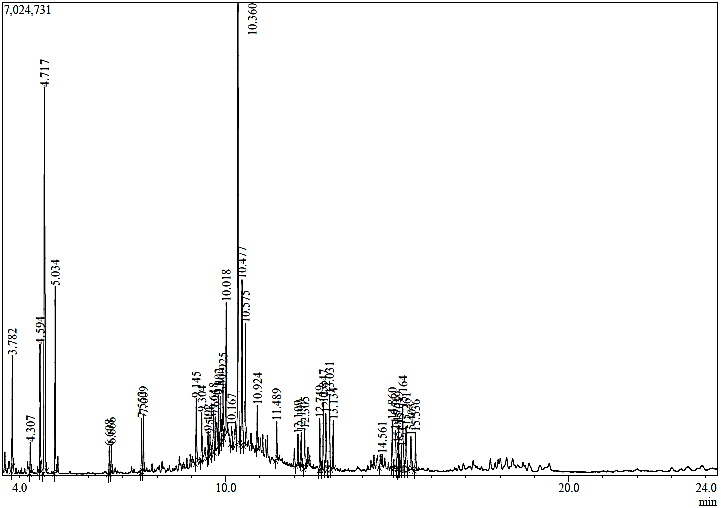


**Week 1**

**Week 3**

**Week 2**


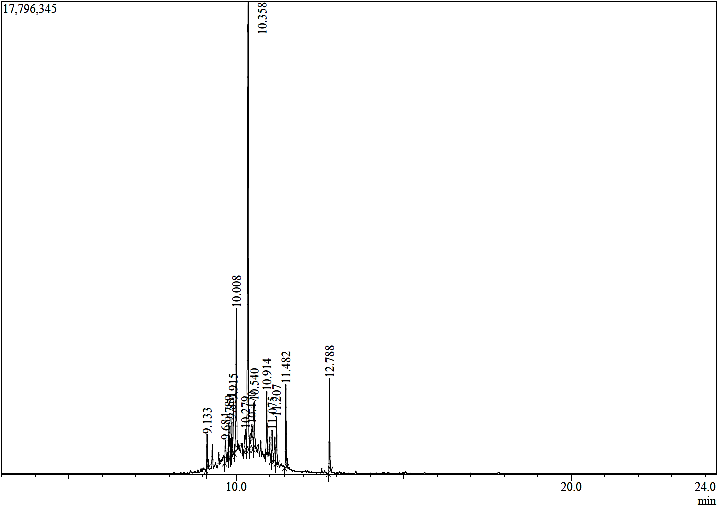

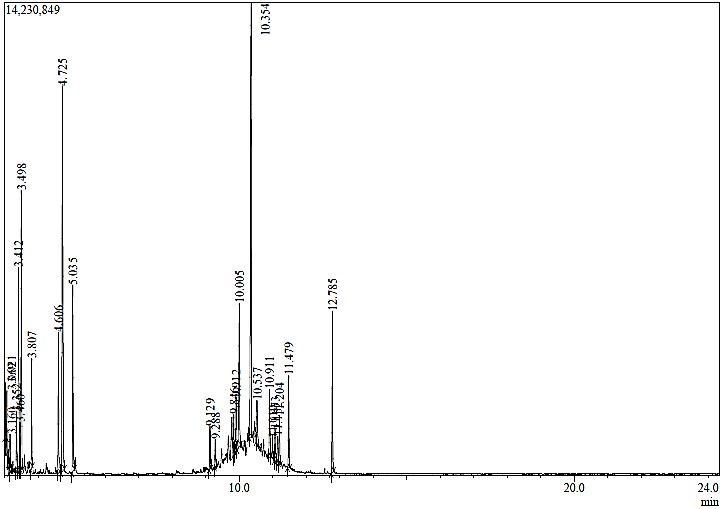


**Week 5**

**Week 4**

**Fig. S12:** The gas chromatogram of spent coolant waste at different weeks of degradation by the wild-type. The numbers on the chromatogram peaks indicates the elution time of the hydrocarbon compounds.

**Supplementary document 13**

**GCMS chromatogram of hydrocarbons of SCW treated by the UV exposed *S. vacuolatus***


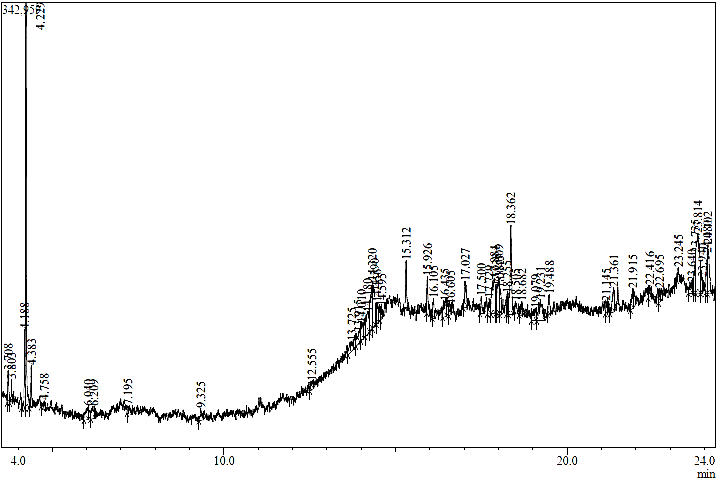

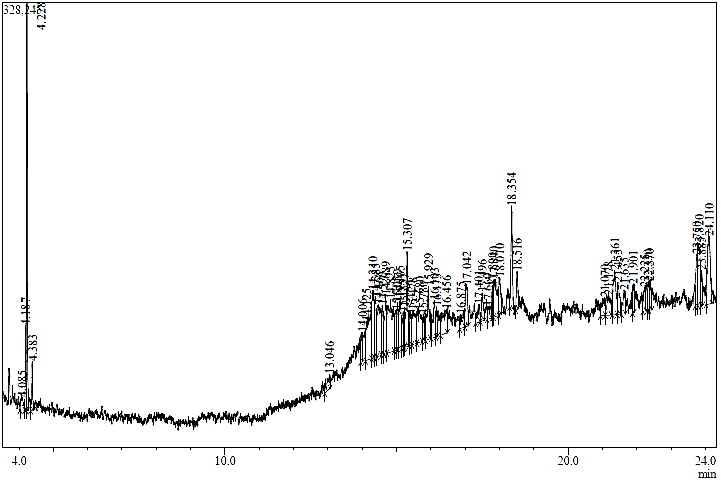

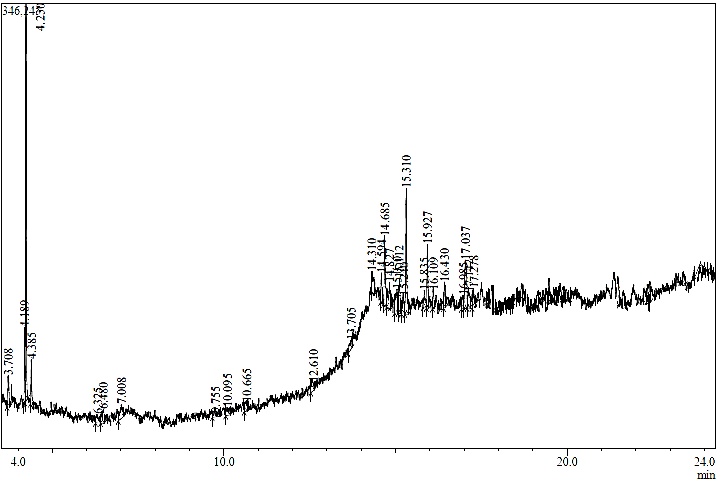


**Week 1**

**Week 3**

**Week 2**


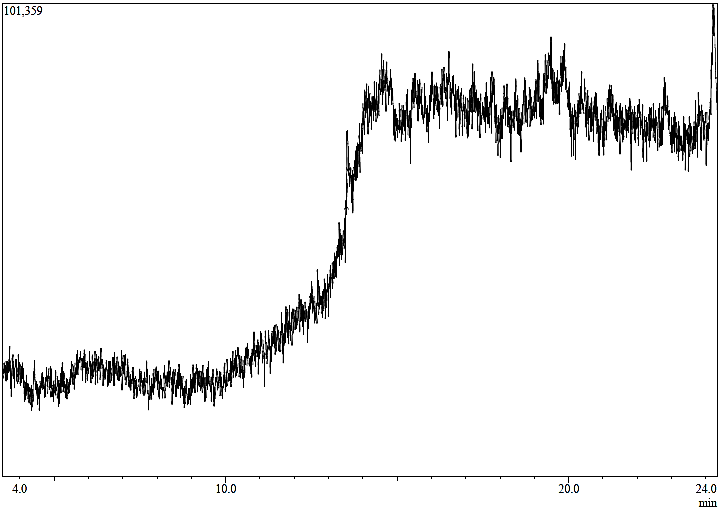

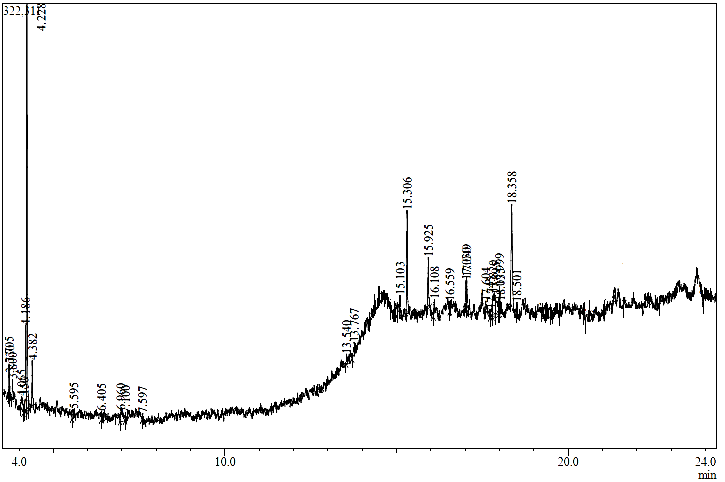


**Week 5**

**Week 4**

**Fig. S13**: The gas chromatogram of spent coolant waste at different weeks of degradation by the UV exposed *S. vacuolatus*. The numbers on the chromatogram peaks indicates the elution time of the hydrocarbon compounds.

**Supplementary document 14**

**Statistical analysis of the biomass concentration at different UV time exposure**

| **ANOVA** | | | | | | |
| --- | --- | --- | --- | --- | --- | --- |
|  | | **Sum of Squares** | **df** | **Mean Square** | **F** | **Sig.** |
| WT | Between Groups | .000 | 6 | .000 | 12.670 | <,001 |
|  | Within Groups | .000 | 14 | .000 |  |  |
|  | Total | .000 | 20 |  |  |  |
| UV_2hrs | Between Groups | .004 | 6 | .001 | 31.164 | <,001 |
|  | Within Groups | .000 | 14 | .000 |  |  |
|  | Total | .004 | 20 |  |  |  |
| UV_4hrs | Between Groups | .008 | 6 | .001 | 12.675 | <,001 |
|  | Within Groups | .002 | 14 | .000 |  |  |
|  | Total | .010 | 20 |  |  |  |
| UV_6hrs | Between Groups | .014 | 6 | .002 | 9.492 | <,001 |
|  | Within Groups | .003 | 14 | .000 |  |  |
|  | Total | .017 | 20 |  |  |  |
| UV_12hrs | Between Groups | .040 | 6 | .007 | 37.084 | <,001 |
|  | Within Groups | .003 | 14 | .000 |  |  |
|  | Total | .043 | 20 |  |  |  |
| UV_24hrs | Between Groups | .062 | 6 | .010 | 32.356 | <,001 |
|  | Within Groups | .004 | 14 | .000 |  |  |
|  | Total | .067 | 20 |  |  |  |
| UV_48hrs | Between Groups | .103 | 6 | .017 | 154.882 | <,001 |
|  | Within Groups | .002 | 14 | .000 |  |  |
|  | Total | .105 | 20 |  |  |  |

**Supplementary document 15**

**Statistical analysis of the Chlorophyll a**

| **ANOVA** | | | | | | |
| --- | --- | --- | --- | --- | --- | --- |
|  | | **Sum of Squares** | **df** | **Mean Square** | **F** | **Sig.** |
| ChLa_D0 | Between Groups | 4.376 | 1 | 4.376 | 8.734 | .042 |
|  | Within Groups | 2.004 | 4 | .501 |  |  |
|  | Total | 6.380 | 5 |  |  |  |
| Chla_D3 | Between Groups | 82.140 | 1 | 82.140 | 2386.542 | <,001 |
|  | Within Groups | .138 | 4 | .034 |  |  |
|  | Total | 82.278 | 5 |  |  |  |
| Chla_D6 | Between Groups | 6.876 | 1 | 6.876 | 55.001 | .002 |
|  | Within Groups | .500 | 4 | .125 |  |  |
|  | Total | 7.376 | 5 |  |  |  |
| Chla_D9 | Between Groups | 21.083 | 1 | 21.083 | 3484.423 | <,001 |
|  | Within Groups | .024 | 4 | .006 |  |  |
|  | Total | 21.107 | 5 |  |  |  |
| Chla_D12 | Between Groups | 14.931 | 1 | 14.931 | 2467.736 | <,001 |
|  | Within Groups | .024 | 4 | .006 |  |  |
|  | Total | 14.955 | 5 |  |  |  |
| Chla_D15 | Between Groups | 27.234 | 1 | 27.234 | 20655.428 | <,001 |
|  | Within Groups | .005 | 4 | .001 |  |  |
|  | Total | 27.239 | 5 |  |  |  |
| Chla_D18 | Between Groups | 1.763 | 1 | 1.763 | 349.027 | <,001 |
|  | Within Groups | .020 | 4 | .005 |  |  |
|  | Total | 1.783 | 5 |  |  |  |
| Chla_D21 | Between Groups | 6.242 | 1 | 6.242 | 10.206 | <,001 |
|  | Within Groups | 2.447 | 4 | .612 |  |  |
|  | Total | 8.689 | 5 |  |  |  |

**Supplementary document 16**

**Statistical analysis of the Chlorophyll b**

| **ANOVA** | | | | | | |
| --- | --- | --- | --- | --- | --- | --- |
|  | | **Sum of Squares** | **df** | **Mean Square** | **F** | **Sig.** |
| ChLb_D0 | Between Groups | 6.786 | 1 | 6.786 | 251.779 | <,001 |
|  | Within Groups | .108 | 4 | .027 |  |  |
|  | Total | 6.894 | 5 |  |  |  |
| Chlb_D3 | Between Groups | 90.863 | 1 | 90.863 | 3449.279 | <,001 |
|  | Within Groups | .105 | 4 | .026 |  |  |
|  | Total | 90.968 | 5 |  |  |  |
| Chlb_D6 | Between Groups | 2.965 | 1 | 2.965 | 143.592 | <,001 |
|  | Within Groups | .083 | 4 | .021 |  |  |
|  | Total | 3.048 | 5 |  |  |  |
| Chlb_D9 | Between Groups | 8.662 | 1 | 8.662 | 974.858 | <,001 |
|  | Within Groups | .036 | 4 | .009 |  |  |
|  | Total | 8.697 | 5 |  |  |  |
| Chlb_D12 | Between Groups | 4.887 | 1 | 4.887 | 184.943 | <,001 |
|  | Within Groups | .106 | 4 | .026 |  |  |
|  | Total | 4.993 | 5 |  |  |  |
| Chlb_D15 | Between Groups | 20.646 | 1 | 20.646 | 3215.661 | <,001 |
|  | Within Groups | .026 | 4 | .006 |  |  |
|  | Total | 20.672 | 5 |  |  |  |
| Chlb_D18 | Between Groups | .180 | 1 | .180 | 50.835 | .002 |
|  | Within Groups | .014 | 4 | .004 |  |  |
|  | Total | .194 | 5 |  |  |  |
| Chlb_D21 | Between Groups | 10.741 | 1 | 10.741 | 1290.654 | <,001 |
|  | Within Groups | .033 | 4 | .008 |  |  |
|  | Total | 10.775 | 5 |  |  |  |

**Supplementary document 17**

**Statistical analysis of the Carotenoid**

| **ANOVA** | | | | | | |
| --- | --- | --- | --- | --- | --- | --- |
|  | | **Sum of Squares** | **df** | **Mean Square** | **F** | **Sig.** |
| Carot_D0 | Between Groups | 6.786 | 1 | 6.786 | 251.779 | <,001 |
|  | Within Groups | .108 | 4 | .027 |  |  |
|  | Total | 6.894 | 5 |  |  |  |
| Carot_D3 | Between Groups | 90.863 | 1 | 90.863 | 3449.279 | <,001 |
|  | Within Groups | .105 | 4 | .026 |  |  |
|  | Total | 90.968 | 5 |  |  |  |
| Carot_D6 | Between Groups | 2.965 | 1 | 2.965 | 143.592 | <,001 |
|  | Within Groups | .083 | 4 | .021 |  |  |
|  | Total | 3.048 | 5 |  |  |  |
| Carot_D9 | Between Groups | 8.662 | 1 | 8.662 | 974.858 | <,001 |
|  | Within Groups | .036 | 4 | .009 |  |  |
|  | Total | 8.697 | 5 |  |  |  |
| Carot_D12 | Between Groups | 4.887 | 1 | 4.887 | 184.943 | <,001 |
|  | Within Groups | .106 | 4 | .026 |  |  |
|  | Total | 4.993 | 5 |  |  |  |
| Carot_D15 | Between Groups | 20.646 | 1 | 20.646 | 3215.661 | <,001 |
|  | Within Groups | .026 | 4 | .006 |  |  |
|  | Total | 20.672 | 5 |  |  |  |
| Carot_D18 | Between Groups | .180 | 1 | .180 | 50.835 | .002 |
|  | Within Groups | .014 | 4 | .004 |  |  |
|  | Total | .194 | 5 |  |  |  |
| Carot_D21 | Between Groups | 10.741 | 1 | 10.741 | 1290.654 | <,001 |
|  | Within Groups | .033 | 4 | .008 |  |  |
|  | Total | 10.775 | 5 |  |  |  |

**Supplementary document 18**

**Statistical analysis of the protein concentration**

| **ANOVA** | | | | | | |
| --- | --- | --- | --- | --- | --- | --- |
|  | | **Sum of Squares** | **df** | **Mean Square** | **F** | **Sig.** |
| PC_D0 | Between Groups | 1.585 | 1 | 1.585 | 2.439 | .193 |
|  | Within Groups | 2.599 | 4 | .650 |  |  |
|  | Total | 4.185 | 5 |  |  |  |
| PC_D3 | Between Groups | 29.384 | 1 | 29.384 | 2661.373 | <,001 |
|  | Within Groups | .044 | 4 | .011 |  |  |
|  | Total | 29.428 | 5 |  |  |  |
| PC_D6 | Between Groups | 8.067 | 1 | 8.067 | 15.632 | .017 |
|  | Within Groups | 2.064 | 4 | .516 |  |  |
|  | Total | 10.131 | 5 |  |  |  |
| PC_D9 | Between Groups | 3.042 | 1 | 3.042 | 5.252 | .084 |
|  | Within Groups | 2.317 | 4 | .579 |  |  |
|  | Total | 5.358 | 5 |  |  |  |
| PC_D12 | Between Groups | 3.878 | 1 | 3.878 | 7.198 | .055 |
|  | Within Groups | 2.155 | 4 | .539 |  |  |
|  | Total | 6.034 | 5 |  |  |  |
| PC_D15 | Between Groups | .913 | 1 | .913 | .851 | .409 |
|  | Within Groups | 4.292 | 4 | 1.073 |  |  |
|  | Total | 5.204 | 5 |  |  |  |
| PC_D18 | Between Groups | .156 | 1 | .156 | .271 | .630 |
|  | Within Groups | 2.313 | 4 | .578 |  |  |
|  | Total | 2.469 | 5 |  |  |  |
| PC_D21 | Between Groups | 76.255 | 1 | 76.255 | 166.405 | <,001 |
|  | Within Groups | 1.833 | 4 | .458 |  |  |
|  | **Total** | **78.088** | **5** |  |  |  |

**Supplementary document 19**

**Statistical analysis of the carbohydrate accumulation**

| **ANOVA** | | | | | | |
| --- | --- | --- | --- | --- | --- | --- |
|  | | **Sum of Squares** | **df** | **Mean Square** | **F** | **Sig.** |
| CHO_D0 | Between Groups | .000 | 1 | .000 | . | . |
|  | Within Groups | .000 | 4 | .000 |  |  |
|  | Total | .000 | 5 |  |  |  |
| CHO_D3 | Between Groups | 37.951 | 1 | 37.951 | 1897567.500 | <,001 |
|  | Within Groups | .000 | 4 | .000 |  |  |
|  | Total | 37.951 | 5 |  |  |  |
| CHO_D6 | Between Groups | 44.652 | 1 | 44.652 | 10625.081 | <,001 |
|  | Within Groups | .017 | 4 | .004 |  |  |
|  | Total | 44.669 | 5 |  |  |  |
| CHO_D9 | Between Groups | 134.663 | 1 | 134.663 | 71897.190 | <,001 |
|  | Within Groups | .007 | 4 | .002 |  |  |
|  | Total | 134.671 | 5 |  |  |  |
| CHO_D12 | Between Groups | 100.099 | 1 | 100.099 | 2780523.375 | <,001 |
|  | Within Groups | .000 | 4 | .000 |  |  |
|  | Total | 100.099 | 5 |  |  |  |
| CHO_D15 | Between Groups | 220.305 | 1 | 220.305 | 1608067.456 | <,001 |
|  | Within Groups | .001 | 4 | .000 |  |  |
|  | Total | 220.306 | 5 |  |  |  |
| CHO_D18 | Between Groups | 564.249 | 1 | 564.249 | 1929056.538 | <,001 |
|  | Within Groups | .001 | 4 | .000 |  |  |
|  | Total | 564.250 | 5 |  |  |  |
| CHO_D21 | Between Groups | 334.357 | 1 | 334.357 | 11529563.793 | <,001 |
|  | Within Groups | .000 | 4 | .000 |  |  |
|  | Total | 334.357 | 5 |  |  |  |

**Supplementary document 20**

**Statistical analysis of the Lipid accumulation**

| **ANOVA** | | | | | | |
| --- | --- | --- | --- | --- | --- | --- |
|  | | **Sum of Squares** | **df** | **Mean Square** | **F** | **Sig.** |
| Lip_D0 | Between Groups | .000 | 1 | .000 | 14.266 | .019 |
|  | Within Groups | .000 | 4 | .000 |  |  |
|  | Total | .000 | 5 |  |  |  |
| Lip_D3 | Between Groups | .000 | 1 | .000 | 9.936 | .034 |
|  | Within Groups | .000 | 4 | .000 |  |  |
|  | Total | .000 | 5 |  |  |  |
| Lip_D6 | Between Groups | .000 | 1 | .000 | 10.521 | .032 |
|  | Within Groups | .000 | 4 | .000 |  |  |
|  | Total | .000 | 5 |  |  |  |
| Lip_D9 | Between Groups | .000 | 1 | .000 | 63.075 | .001 |
|  | Within Groups | .000 | 4 | .000 |  |  |
|  | Total | .000 | 5 |  |  |  |
| Lip_D12 | Between Groups | .000 | 1 | .000 | 11.286 | .028 |
|  | Within Groups | .000 | 4 | .000 |  |  |
|  | Total | .000 | 5 |  |  |  |
| Lip_D15 | Between Groups | .000 | 1 | .000 | 19.898 | .011 |
|  | Within Groups | .000 | 4 | .000 |  |  |
|  | Total | .000 | 5 |  |  |  |
| Lip_D18 | Between Groups | .000 | 1 | .000 | 71.813 | .001 |
|  | Within Groups | .000 | 4 | .000 |  |  |
|  | Total | .000 | 5 |  |  |  |
| Lip_D21 | Between Groups | .000 | 1 | .000 | 261.664 | <,001 |
|  | Within Groups | .000 | 4 | .000 |  |  |
|  | Total | .000 | 5 |  |  |  |

**Supplementary document 21**

**Statistical analysis of the substrate versatility**

**Na2CO3**

| **ANOVA** | | | | | | |
| --- | --- | --- | --- | --- | --- | --- |
|  | | **Sum of Squares** | **df** | **Mean Square** | **F** | **Sig.** |
| Na2CO3_D0 | Between Groups | .778 | 1 | .778 | 2.596 | .182 |
|  | Within Groups | 1.198 | 4 | .300 |  |  |
|  | Total | 1.976 | 5 |  |  |  |
| Na2CO3_D3 | Between Groups | .019 | 1 | .019 | 361.387 | <,001 |
|  | Within Groups | .000 | 4 | .000 |  |  |
|  | Total | .019 | 5 |  |  |  |
| Na2CO3_D6 | Between Groups | .017 | 1 | .017 | 8.211 | .046 |
|  | Within Groups | .008 | 4 | .002 |  |  |
|  | Total | .025 | 5 |  |  |  |
| Na2CO3_D9 | Between Groups | .023 | 1 | .023 | 2.445 | .193 |
|  | Within Groups | .038 | 4 | .010 |  |  |
|  | Total | .062 | 5 |  |  |  |
| Na2CO3_D12 | Between Groups | .095 | 1 | .095 | 49.925 | .002 |
|  | Within Groups | .008 | 4 | .002 |  |  |
|  | Total | .103 | 5 |  |  |  |
| Na2CO3_D15 | Between Groups | .117 | 1 | .117 | 898.165 | <,001 |
|  | Within Groups | .001 | 4 | .000 |  |  |
|  | Total | .117 | 5 |  |  |  |
| Na2CO3_D18 | Between Groups | .090 | 1 | .090 | 12.484 | .024 |
|  | Within Groups | .029 | 4 | .007 |  |  |
|  | Total | .119 | 5 |  |  |  |
| Na2CO3_D21 | Between Groups | .183 | 1 | .183 | 82.002 | <,001 |
|  | Within Groups | .009 | 4 | .002 |  |  |
|  | Total | .192 | 5 |  |  |  |

**Supplementary document 22**

**Statistical analysis of the substrate versatility**

**Glucose**

| **ANOVA** | | | | | | |
| --- | --- | --- | --- | --- | --- | --- |
|  | | **Sum of Squares** | **df** | **Mean Square** | **F** | **Sig.** |
| Glucose_D0 | Between Groups | .009 | 1 | .009 | 3.512 | .134 |
|  | Within Groups | .010 | 4 | .003 |  |  |
|  | Total | .020 | 5 |  |  |  |
| Glucose_D3 | Between Groups | .010 | 1 | .010 | 20.063 | .011 |
|  | Within Groups | .002 | 4 | .000 |  |  |
|  | Total | .012 | 5 |  |  |  |
| Glucose_D6 | Between Groups | .245 | 1 | .245 | 20.875 | .010 |
|  | Within Groups | .047 | 4 | .012 |  |  |
|  | Total | .292 | 5 |  |  |  |
| Glucose_D9 | Between Groups | .002 | 1 | .002 | .234 | .654 |
|  | Within Groups | .037 | 4 | .009 |  |  |
|  | Total | .039 | 5 |  |  |  |
| Glucose_D12 | Between Groups | .003 | 1 | .003 | .684 | .455 |
|  | Within Groups | .020 | 4 | .005 |  |  |
|  | Total | .024 | 5 |  |  |  |
| Glucose_D15 | Between Groups | .033 | 1 | .033 | 99.413 | <,001 |
|  | Within Groups | .001 | 4 | .000 |  |  |
|  | Total | .034 | 5 |  |  |  |
| Glucose_D18 | Between Groups | .050 | 1 | .050 | 441.606 | <,001 |
|  | Within Groups | .000 | 4 | .000 |  |  |
|  | Total | .051 | 5 |  |  |  |
| Glucose_D21 | Between Groups | .077 | 1 | .077 | 76.216 | <,001 |
|  | Within Groups | .004 | 4 | .001 |  |  |
|  | Total | .081 | 5 |  |  |  |

**Supplementary document 23**

**Statistical analysis of the substrate versatility**

**Glycerol**

| **ANOVA** | | | | | | |
| --- | --- | --- | --- | --- | --- | --- |
|  | | **Sum of Squares** | **df** | **Mean Square** | **F** | **Sig.** |
| Gly_D0 | Between Groups | .002 | 1 | .002 | 67.328 | .001 |
|  | Within Groups | .000 | 4 | .000 |  |  |
|  | Total | .002 | 5 |  |  |  |
| Gly_D3 | Between Groups | .002 | 1 | .002 | 1464.100 | <,001 |
|  | Within Groups | .000 | 4 | .000 |  |  |
|  | Total | .002 | 5 |  |  |  |
| Gly_D6 | Between Groups | .009 | 1 | .009 | 172.969 | <,001 |
|  | Within Groups | .000 | 4 | .000 |  |  |
|  | Total | .009 | 5 |  |  |  |
| Gly_D9 | Between Groups | .126 | 1 | .126 | 10788.014 | <,001 |
|  | Within Groups | .000 | 4 | .000 |  |  |
|  | Total | .126 | 5 |  |  |  |
| Gly_D12 | Between Groups | .010 | 1 | .010 | 22.197 | .009 |
|  | Within Groups | .002 | 4 | .000 |  |  |
|  | Total | .011 | 5 |  |  |  |
| Gly_D15 | Between Groups | .012 | 1 | .012 | 1275.586 | <,001 |
|  | Within Groups | .000 | 4 | .000 |  |  |
|  | Total | .012 | 5 |  |  |  |
| Gly_D18 | Between Groups | .018 | 1 | .018 | 377.173 | <,001 |
|  | Within Groups | .000 | 4 | .000 |  |  |
|  | Total | .019 | 5 |  |  |  |
| Gly_D21 | Between Groups | .007 | 1 | .007 | 30.991 | .005 |
|  | Within Groups | .001 | 4 | .000 |  |  |
|  | Total | .008 | 5 |  |  |  |

**Supplementary document 24**

**Statistical analysis of the dehydrogenase activity (****TPF production) and TPH degradation during degradation**

**Table 4: Accumulated analysis of variance for TPF production and TPH degradation**

| **Parameters** | **Source of variation** | **df** | **Significant test of TPF production and TPH biodegradation** | | | |  |
| --- | --- | --- | --- | --- | --- | --- | --- |
|  |  |  | **Week 1** | **Week 2** | **Week 3** | **Week 4** | **Week 5** |
| **TPF production** | Replicates | 6 | 0.0001382*** | 0.0005708*** | 0.001386** | 0.00011962*** | 0.0001087*** |
|  | Treatment | 1 | 0.2940601 | 2.4186258 | 1.396720 | 0.15414007 | 0.0582435* |
|  | Error | 6 | 0.0001969*** | 0.0003701*** | 0.001108** | 0.00007190*** | 0.0001222*** |
|  |  |  |  |  |  |  |  |
| **TPH biodegradation** | Replicates | 6 | 0.0002279*** | 0.00007370*** | 0.00001615*** | 0.00011985*** | 0.00001428*** |
|  | Treatment | 1 | 0.1307964 | 0.14384606 | 0.09809640 | 0.06709633 | 2.01476579 |
|  | Error | 6 | 0.0002226*** | 0.00007928*** | 0.00002013*** | 0.00005173*** | 0.00001428*** |

Key: df: degrees of freedom; TPF: triphenyl formazan; TPH: total petroleum hydrocarbon; asterisks *, **, and ***, indicates various levels of significance at P < 0.05; P < 0.01; P < 0.001, respectively. Mean of six replicates (n=6). This was extrapolated from the statistical analysis and summarised.

**Supplementary document 25**

**Statistical analysis for dehydrogenase activity analysis of wild-type and UV exposed *S. vacuolatus***

Analysis of variance

Variate: week1

Source of variation d.f. s.s. m.s. v.r. F pr.

Replicates stratum 6 0.0008294 0.0001382 0.70

Replicates.*Units* stratum

Treatment 1 0.2940601 0.2940601 1493.41 <.001

Residual 6 0.0011814 0.0001969

Total 13 0.2960709

Tables of means

Variate: week1

Grand mean 0.4901

Treatment A B

0.3451 0.6350

Standard errors of differences of means

Table Treatment

rep. 7

d.f. 6

s.e.d. 0.00750

Analysis of variance

Variate: week2

Source of variation d.f. s.s. m.s. v.r. F pr.

Replicates stratum 6 0.0034247 0.0005708 1.54

Replicates.*Units* stratum

Treatment 1 2.4186258 2.4186258 6534.72 <.001

Residual 6 0.0022207 0.0003701

Total 13 2.4242712

Tables of means

Variate: week2

Grand mean 0.9346

Treatment A B

0.5190 1.3503

Standard errors of differences of means

Table Treatment

rep. 7

d.f. 6

s.e.d. 0.01028

Analysis of variance

Variate: week3

Source of variation d.f. s.s. m.s. v.r. F pr.

Replicates stratum 6 0.008314 0.001386 1.25

Replicates.*Units* stratum

Treatment 1 1.396720 1.396720 1260.25 <.001

Residual 6 0.006650 0.001108

Total 13 1.411684

Tables of means

Variate: week3

Grand mean 1.4549

Treatment A B

1.1390 1.7707

Standard errors of differences of means

Table Treatment

rep. 7

d.f. 6

s.e.d. 0.01779

Analysis of variance

Variate: week4

Source of variation d.f. s.s. m.s. v.r. F pr.

Replicates stratum 6 0.00071771 0.00011962 1.66

Replicates.*Units* stratum

Treatment 1 0.15414007 0.15414007 2143.67 <.001

Residual 6 0.00043143 0.00007190

Total 13 0.15528921

*Message: the following units have large residuals.*

Replicates 2 *units* 1 0.0114 s.e. 0.0056

Replicates 2 *units* 2 -0.0114 s.e. 0.0056

Tables of means

Variate: week4

Grand mean 0.3276

Treatment A B

0.2227 0.4326

Standard errors of differences of means

Table Treatment

rep. 7

d.f. 6

s.e.d. 0.00453

Analysis of variance

Variate: week5

Source of variation d.f. s.s. m.s. v.r. F pr.

Replicates stratum 6 0.0006524 0.0001087 0.89

Replicates.*Units* stratum

Treatment 1 0.0582435 0.0582435 476.75 <.001

Residual 6 0.0007330 0.0001222

Total 13 0.0596289

*Message: the following units have large residuals.*

Replicates 5 0.0146 s.e. 0.0068

Replicates 3 *units* 1 0.0155 s.e. 0.0072

Replicates 3 *units* 2 -0.0155 s.e. 0.0072

Tables of means

Variate: week5

Grand mean 0.1849

Treatment A B

0.1204 0.2494

Standard errors of differences of means

Table Treatment

rep. 7

d.f. 6

s.e.d. 0.00591

**Supplementary document 26**

**Statistical analysis for GCMS total petroleum hydrocarbon (TPH) of wildtype and UV exposed *S. vacuolatus***

Analysis of variance

Variate: week1

Source of variation d.f. s.s. m.s. v.r. F pr.

Replicates stratum 6 0.0013673 0.0002279 1.02

Replicates.*Units* stratum

Treatment 1 0.1307964 0.1307964 587.52 <.001

Residual 6 0.0013358 0.0002226

Total 13 0.1334995

Tables of means

Variate: week1

Grand mean 0.5555

Treatment A B

0.4589 0.6522

Standard errors of differences of means

Table Treatment

rep. 7

d.f. 6

s.e.d. 0.00798

Least significant differences of means (5% level)

Table Treatment

rep. 7

d.f. 6

l.s.d. 0.01952

Analysis of variance

Variate: week2

Source of variation d.f. s.s. m.s. v.r. F pr.

Replicates stratum 6 0.00044221 0.00007370 0.93

Replicates.*Units* stratum

Treatment 1 0.14384606 0.14384606 1814.34 <.001

Residual 6 0.00047570 0.00007928

Total 13 0.14476396

Tables of means

Variate: week2

Grand mean 0.7244

Treatment A B

0.6230 0.8257

Standard errors of differences of means

Table Treatment

rep. 7

d.f. 6

s.e.d. 0.00476

Least significant differences of means (5% level)

Table Treatment

rep. 7

d.f. 6

l.s.d. 0.01165

Analysis of variance

Variate: week3

Source of variation d.f. s.s. m.s. v.r. F pr.

Replicates stratum 6 0.00009689 0.00001615 0.80

Replicates.*Units* stratum

Treatment 1 0.09809640 0.09809640 4874.18 <.001

Residual 6 0.00012075 0.00002013

Total 13 0.09831404

Tables of means

Variate: week3

Grand mean 0.7996

Treatment A B

0.7159 0.8833

Standard errors of differences of means

Table Treatment

rep. 7

d.f. 6

s.e.d. 0.00240

Least significant differences of means (5% level)

Table Treatment

rep. 7

d.f. 6

l.s.d. 0.00587

Analysis of variance

Variate: week4

Source of variation d.f. s.s. m.s. v.r. F pr.

Replicates stratum 6 0.00071907 0.00011985 2.32

Replicates.*Units* stratum

Treatment 1 0.06709633 0.06709633 1297.01 <.001

Residual 6 0.00031039 0.00005173

Total 13 0.06812579

Tables of means

Variate: week4

Grand mean 0.8533

Treatment A B

0.7841 0.9226

Standard errors of differences of means

Table Treatment

rep. 7

d.f. 6

s.e.d. 0.00384

Least significant differences of means (5% level)

Table Treatment

rep. 7

d.f. 6

l.s.d. 0.00941

Analysis of variance

Variate: week5

Source of variation d.f. s.s. m.s. v.r. F pr.

Replicates stratum 6 0.00008565 0.00001428 1.00

Replicates.*Units* stratum

Treatment 1 2.01476579 2.01476579 1.411E+05 <.001

Residual 6 0.00008565 0.00001428

Total 13 2.01493709

Tables of means

Variate: week5

Grand mean 0.4794

Treatment A B

0.8587 0.1000

Standard errors of differences of means

Table Treatment

rep. 7

d.f. 6

s.e.d. 0.00202

Least significant differences of means (5% level)

Table Treatment

rep. 7

d.f. 6

l.s.d. 0.00494
